# Supplementary material for: Knowledge and beliefs regarding cervical cancer screening and HPV vaccination among urban and rural women in León, Nicaragua
Source: PeerJ. 2017 Oct 25;5:e3871. doi: 10.7717/peerj.3871 (PMC5660604; doi:10.7717/peerj.3871)
Supplement: Supplemental Information 6 [file peerj-05-3871-s006.doc]

**Accessibility**

Title: Mantica-2.docx

Descriptor Info: Age: Interview Location: Residence: Interviewer: Date of Interview:

Codes Applied: Accessibility

Excerpt Package: 1881

Excerpt End: 2069

ALEXANDRA: Ha oído hablar de la prueba de Papanicolaou alguna vez y se ha realizado alguna

MUJER: Sí, sí.

ALEXANDRA: Y se recomienda aquí los centros de salud

MUJER: Sí, yo aquí me lo hago

Title: Mantica-6.docx

Descriptor Info: Age: Interview Location: Residence: Interviewer: Date of Interview:

Codes Applied: Accessibility

Excerpt Package: 8818

Excerpt End: 8894

ALEXANDRA: Es difícil recibir una prueba de pap?

MUJER: No, no están difícil

Title: Mantica-2.docx

Descriptor Info: Age: Interview Location: Residence: Interviewer: Date of Interview:

Codes Applied: Accessibility

Excerpt Package: 242

Excerpt End: 639

MUJER: Yo siempre he venido aquí al mantica y me atiende bien los médicos, es mucho mejor aquí en el mantica que en los centros de la comunidad, por lo menos yo soy de Monte redondo

ALEXANDRA: Es un área urbana o rural?

MUJER: Es urbano, pero yo siento que es mejor aquí la atención

ALEXANDRA: Es mucha distancia que usted viaja ´para venir aquí

MUJER: Si tengo que agarrar un bus para venir aquí

Title: Mantica-6.docx

Descriptor Info: Age: Interview Location: Residence: Interviewer: Date of Interview:

Codes Applied: Accessibility

Excerpt Package: 777

Excerpt End: 1101

ALEXANDRA: Y usted también puede recibir la salud sexual, tratamiento y consejos aquí?

MUJER: Si aquí nos ayudan con la salud sexual de la mujer activa ya sea protección por medio del pap, una vez haciéndote el pap si te dan resultado y te sale algo te dan tratamiento y te siguen la secuencia hasta que sales de tu problema

Title: Mantica-5.docx

Descriptor Info: Age: Interview Location: Residence: Interviewer: Date of Interview:

Codes Applied: Accessibility

Excerpt Package: 1032

Excerpt End: 1305

HANNAH: Ha recibido una prueba de Papanicolaou alguna vez?

MUJER: Si

HANNAH: Y cuantas veces se la ha hecho?

MUJER: Infinidades de veces me lo han hecho, cada ocho meses me lo hago aquí

HANNAH: Cada ocho meses y durante sus embarazos se los hizo también?

MUJER: Si, también

Title: Los Leches-1 (1).docx

Descriptor Info: Age: Interview Location: Residence: Interviewer: Date of Interview:

Codes Applied: Accessibility Attendance

Excerpt Package: 3862

Excerpt End: 3943

Sara: After that, have you received pap tests frequently?

Woman: Yes, every year.

Title: Poneloya-2.docx

Descriptor Info: Age: Interview Location: Residence: Interviewer: Date of Interview:

Codes Applied: Accessibility Attendance Pap smear

Excerpt Package: 14

Excerpt End: 708

ALEXA: Entonces para empezar podría hablarme un poco de su salud o sobre su experiencia con el cuidado de salud en general?

MUJER: Pues por los momentos yo siempre me he cuidado con respecto al Pap, de que si me siente algo o algún malestar, yo vengo, si están los de aquí, aquí vengo porque son mis médicos sino si es un fin de semana yo voy donde medico pagado porque a veces yo padezco de unas nauseas de la gastritis cuando a mí me da eso yo me pongo fatal a mime dan mareos, vómitos y entonces yo tengo que ir donde el medico que este cerca, porque yo paso fatal solo con mis ojitos cerrados y con una almohada hasta que ya se me pasa eso, pero por lo general siempre vengo aquí al centro.

**Attendance**

Title: Los Leches-3.docx

Descriptor Info: Age: Interview Location: Residence: Interviewer: Date of Interview:

Codes Applied: Attendance

Excerpt Package: 167

Excerpt End: 555

SARA: Gracias, dígame acerca de su experiencia con el cuidado de salud, viene a este centro normalmente?

MUJER: Si, normalmente pero vengo más por la niña

SARA: Y como fue el cuidado de salud?

MUJER: La verdad es que me siento aquí porque las muchachas atiende bien la Brenda, la Lupe, yo tengo bastante tiempo de venir aquí, desde que estaba la mama de la Lupe y siento que atienden bien

Title: Santa Ana-2.docx

Descriptor Info: Age: Interview Location: Residence: Interviewer: Date of Interview:

Codes Applied: Attendance

Excerpt Package: 525

Excerpt End: 760

ALEXANDRA: Y usted viene regularmente a este puesto de Salud?

MUJER: Si, aquí siempre vengo aquí es donde me miro mis enfermedades, pero que son leves como una inflamación que es algo común pero nada grave, pero de ahí otras cosas no.

Title: Mantica-6.docx

Descriptor Info: Age: Interview Location: Residence: Interviewer: Date of Interview:

Codes Applied: Attendance

Excerpt Package: 5884

Excerpt End: 6191

ALEXANDRA: Y el papiloma es muy común entre las mujeres nicaragüenses?

MUJER: Es un virus silencioso por eso es que hay estarse chequeando porque a veces hay personas que lo tienen y no lo saben por eso es que hay que acercarse más a los centros de salud para que uno lo sepa por qué nosotros no lo sabemos

Title: Mantica-5.docx

Descriptor Info: Age: Interview Location: Residence: Interviewer: Date of Interview:

Codes Applied: Attendance

Excerpt Package: 293

Excerpt End: 528

HANNAH: Y usualmente viene a este centro de Salud?

MUJER: Sí

HANNAH: Cada año viene usted aquí para recibir algo acerca de su cuidado

MUJER: Bueno, yo vengo seguido aquí, porque tengo tres niños y todos pasan consulta aquí y yo también

Title: Mantica-6.docx

Descriptor Info: Age: Interview Location: Residence: Interviewer: Date of Interview:

Codes Applied: Attendance

Excerpt Package: 9051

Excerpt End: 9661

ALEXANDRA: Y hay algo más acerca del tema de las mujeres o la salud que quieres discutir o compartir?

MUJER: Solamente que asistamos a los centros de salud por que es donde obtenemos la primera información por que la tecnología también ayuda pero en los centros es donde la información más eficaz porque son ellos los que la están estudiando y aunque la tecnología no se queda atrás pero en los centros es donde uno aprende más con los médicos y uno se siente más tranquilo

ALEXANDRA: Y por esta razón es que es más difícil comunicarse con las mujeres del campo?

MUJER: Sí, es más difícil comunicarse con ellas

Title: Los Leches-2.docx

Descriptor Info: Age: Interview Location: Residence: Interviewer: Date of Interview:

Codes Applied: Attendance

Excerpt Package: 263

Excerpt End: 423

HANNAH: Y usualmente viene a este centro de salud cuando necesita cuidado?

MUJER: Si

HANNAH: Y usted vive cerca de aquí en el campo?

MUJER: Si, aquí en el campo

Title: Subtiava-2.docx

Descriptor Info: Age: Interview Location: Residence: Interviewer: Date of Interview:

Codes Applied: Attendance

Excerpt Package: 658

Excerpt End: 1536

HANNAH: Dígame acerca de su experiencia con el cuidado de la salud sexual y la educación sexual?

MUJER: Bueno, cada vez que me tocaba venir a planificar para no quedar embarazada, asistía al centro a que me inyectaran y habían muchachos que daban charlas sobre educación sexual sobre las enfermedades de transmisión sexual siempre me atendieron con buena manera, y recibí la mayoría de las charlas aquí en el centro y las muchachas decían que debíamos de hacernos los exámenes año a año para no tener ningún tipo de enfermedad y poder prevenir algún tipo de cáncer.

HANNAH: Ah bueno, una vez cada año usted recibe una prueba de Papanicolaou?

MUJER: Si, bueno eso nos recomiendan y al menos yo así lo hago año con año porque me dicen que es bueno y así pueda rendirle a mis hijos y por eso yo me las hago y ellas dicen es anualmente para también detectar cualquier tipo de cáncer

Title: Mantica-6.docx

Descriptor Info: Age: Interview Location: Residence: Interviewer: Date of Interview:

Codes Applied: Attendance

Excerpt Package: 3571

Excerpt End: 3969

ALEXANDRA: Ha escuchado mucho hablar o habla acerca del cáncer cervical o cáncer de cuello uterino?

MUJER: Son pocos casos los que hay porque son pocas personas las que se están constantemente en chequeo pero las que están actualmente siempre están con su tratamiento pero hay personas que no acuden a los centros de salud las rurales principalmente entonces ahí es donde hay que llevar las charlas

Title: Mantica-6.docx

Descriptor Info: Age: Interview Location: Residence: Interviewer: Date of Interview:

Codes Applied: Attendance

Excerpt Package: 5352

Excerpt End: 5883

ALEXANDRA: Y ha oído del virus de papiloma humano antes de hoy?

MUJER: cercano a mí a mis alrededores con mis familiares no, pero si por medio del internet y personas que se han relacionado con personas que se ven demasiado tarde lo he escuchado y lo he seguido demasiado cerca para saber más sobre los resultados y que te da, es donde te da temor y por eso yo asiste con más frecuencia a los centros de salud por eso es que hay que sacarlo del tabú y hablarlo más para que uno no se alarme para que se sepa de que hay que cuidarse

Title: Santa Ana-3.docx

Descriptor Info: Age: Interview Location: Residence: Interviewer: Date of Interview:

Codes Applied: Attendance

Excerpt Package: 2461

Excerpt End: 3349

HANNAH: Usted tiene una experiencia de alguien que tiene cáncer de cuello uterino, un miembro de su familia, amigos?

MUJER: Si, con alguien de mi familia, y ya falleció una de cáncer de cuello uterino por eso es que yo invito a muchas personas a que se revisen a las mujeres

HANNAH: Un miembro cercano de su familia, como tía?

MUJER: Si, era una tía.

HANNAH: Lo siento, puede hablar un poco de su experiencia o de la experiencia de su tía?

MUJER: Bueno cuando mi tía sufrió esa enfermedad del cáncer de cuello uterino, ella era tan hermosa y después se puso tan delgadita, le hicieron quimioterapias, pero ella ya se lo detectaron muy avanzado ya que ella no acostumbraba a hacerse el pap entonces se lo diagnosticaron ya muy avanzado, las quimioterapias ya no le ayudaban porque el cáncer ya estaba en un término entonces se combatió pero no se curó y son muy dolorosos los tratamientos.

Title: Mantica-5.docx

Descriptor Info: Age: Interview Location: Residence: Interviewer: Date of Interview:

Codes Applied: Attendance

Excerpt Package: 3946

Excerpt End: 4174

HANNAH: Después de lo de su amiga piensa que le afecta e influye a que se haga ms pruebas de paps?

MUJER: Ah sí, ahora lo más que tardo son ocho meses en hacerme el examen y me hago uno aquí en el Centro de Salud y otro privado

Title: Perla-2.docx

Descriptor Info: Age: Interview Location: Residence: Interviewer: Date of Interview:

Codes Applied: Attendance Time

Excerpt Package: 10

Excerpt End: 1498

ALEXANDRA: Bueno, entonces para empezar puedes hablarme un poco sobre el cuidado de salud en general?

MUJER: Aquí como lo tratan a uno? Pues la verdad mal porque primeramente yo estaba con la enfermera porque primeramente me mandan una cita y me dicen que la venga a agarrar aquí, después me dijeron que tenía que venir personalmente la muchacha porque es para mi hija y les digo de que ella está trabajando que no puede venir y me dicen que tiene que venir ella sino no me dan la cita, vengo y hablo con el Licenciado, el me dio una orden para que ella me atendiera pero ella me dijo muchas cosas, diciendo que no me podía atender por que a ella no le pertenecía esa área ya le dije que es allá, si le dije pero yo solo quería información entonces me mandaron al otro lugar y me atendieron ahora que vengo a la cita me dijeron que tenía que traer la cedula entonces le dije yo que en el papel venia la cedula, no me dice es que es un requisito nuestro que deben traer cedula, entonces yo le dije que estábamos haciendo la gran cola de la fila y volver a ir a casa y regresar otra vez a hacer de nuevo fila, no era justo y le dije que por favor me atendiera entonces me dijo de que no podía, así que fui donde el médico y el me dio un papelito y me dijo que si, que me podían atender entonces dijo ella, les voy a hacer el favor pero yo no puedo hacer esto para en otra traigan su cedula, eso es todo lo que nos debes de decir dije yo, tranquila, entonces tuve ese inconveniente con ella.

**Care**

Title: Mantica-4.docx

Descriptor Info: Age: Interview Location: Residence: Interviewer: Date of Interview:

Codes Applied: Care

Excerpt Package: 6317

Excerpt End: 6952

HANNAH: Mi última pregunta es, si pudiera cambiar algo de su tratamiento o de la información que ha recibido al descifrar su diagnóstico que sería?

MUJER: Bueno, no sé si la pregunta es si puedo cambiar el tratamiento

HANNAH: En una parte de su experiencia con el cáncer, con los centros de salud, con el hospital quiere cambiar algo o fue algo bueno su relación con los medios de salud?

MUJER: Si, fue todo muy bueno, con los centros de salud y el hospital

HANNAH: Recibe información de cada parte que necesita? Un buen apoyo?

MUJER: Si muy buena información y estoy agradecida con el hospital porque está haciendo muy bien su trabajo

Title: SantaAna-4.docx.docx

Descriptor Info: Age: Interview Location: Residence: Interviewer: Date of Interview:

Codes Applied: Care

Excerpt Package: 3834

Excerpt End: 4097

ALEXANDRA: Y porque cree usted que hay mujeres que deciden no recibir una prueba de Pap?

MUJER: Bueno no lo sé pero yo digo que es porque a las mujeres no les gusta o no sé, pero aquí en el centro nos atienden mejor pero hay mujeres que no sé porque no les gusta.

Title: Los Leches-3.docx

Descriptor Info: Age: Interview Location: Residence: Interviewer: Date of Interview:

Codes Applied: Care

Excerpt Package: 2374

Excerpt End: 2692

SARA: Quien le ha realizado la prueba de pap?

MUJER: Aquí solo me la ha hecho la enfermera que se llama Marlene aquí en el centro

SARA: Cuáles son sus preocupaciones con la prueba de pap es dolorosa, piensa o que es intima?

MUJER: Depende porque si le duele porque hay enfermeras que es suave la mano hay otras que no

Title: Mantica-6.docx

Descriptor Info: Age: Interview Location: Residence: Interviewer: Date of Interview:

Codes Applied: Care

Excerpt Package: 2492

Excerpt End: 2992

ALEXANDRA: Y como es la actitud de las enfermeras que realizan las pruebas de pap?

MUJER: Su actitud?

ALEXANDRA: Sí, su actitud hace que se sientan cómodas o incomodas?

MUJER: Cómodas porque somos mujeres, ellas atienden muy bien y hacen muy bien todo

ALEXANDRA: Entonces si fuera un enfermero o un médico varón no se sentiría cómoda

MUJER: Depende, si se hace acompañar de una mujer es mas cómodo, hacen igual el trabajo pero uno está acostumbrado que sea una mujer por que así uno se desplaza mejor

Title: Mantica-4.docx

Descriptor Info: Age: Interview Location: Residence: Interviewer: Date of Interview:

Codes Applied: Care

Excerpt Package: 6317

Excerpt End: 6952

HANNAH: Mi última pregunta es, si pudiera cambiar algo de su tratamiento o de la información que ha recibido al descifrar su diagnóstico que sería?

MUJER: Bueno, no sé si la pregunta es si puedo cambiar el tratamiento

HANNAH: En una parte de su experiencia con el cáncer, con los centros de salud, con el hospital quiere cambiar algo o fue algo bueno su relación con los medios de salud?

MUJER: Si, fue todo muy bueno, con los centros de salud y el hospital

HANNAH: Recibe información de cada parte que necesita? Un buen apoyo?

MUJER: Si muy buena información y estoy agradecida con el hospital porque está haciendo muy bien su trabajo

Title: Mantica-5.docx

Descriptor Info: Age: Interview Location: Residence: Interviewer: Date of Interview:

Codes Applied: Care

Excerpt Package: 1617

Excerpt End: 1801

HANNAH: Quince días, ok, piensa que las pruebas de pap son dolorosas o incomodas?

MUJER: No, yo nunca he sentido dolor, a mí siempre mis Pap me los ha hecho la Doctora no la enfermera.

Title: Los Leches-2.docx

Descriptor Info: Age: Interview Location: Residence: Interviewer: Date of Interview:

Codes Applied: Care

Excerpt Package: 2142

Excerpt End: 2305

HANNAH: Ha tenido una experiencia mala en el pasado?

MUJER: No, nunca he tenido mala experiencia

HANNAH: Se sentiría cómoda recibiendo una prueba de pap?

MUJER: Si

Title: Perla-4.docx

Descriptor Info: Age: Interview Location: Residence: Interviewer: Date of Interview:

Codes Applied: Care

Excerpt Package: 8581

Excerpt End: 10673

HANNAH: Una pregunta más, ha tenido una mala experiencia aquí en el centro de salud o sabe de alguien que la haya tenido con las pruebas de pap o algún chequeo?

MUJER: Pues creo que no, tal vez a veces creo que es cuestión de opinión de los médicos o no están bien orientados sobre las medicinas que dan o indican al paciente por cualquier enfermedad cuando uno tiene un hongo o cosas así, usted sabe cuando uno se hace el pap como que no dan el tratamiento que deberían

HANNAH: Hay confusión con respecto del tratamiento?

MUJER: Si, exacto, porque por ejemplo eso paso con una hija mía porque la doctora le dio un tratamiento y parece era muy pesado para ella y ella en ese tiempo no tenía marido y le mando una pastilla y mi hermana se la miro y le dijo que esa no era la pastilla indicada para ella pero pues no trascendió para nada malo simplemente se la suspendió y bebió de otra por su propia cuenta porque mi hermana se la indico pero en cuanto a otra gravedad no, lo que le quiero decir es que por ejemplo si un médico le dice que tiene una infección en la orina ese médico le receta una cosa pero si va donde otro le receta otras cosas

HANNAH: Son cosas diferentes de personas diferentes?

MUJER: Si, pero tal vez no es que sean malos pero tal vez el otro le dio mejor medicamento porque todo varia por la edad o lo que uno padece y tal vez unos no son los indicados

HANNAH: Y piensa usted que cada médico pasa suficiente tiempo con el paciente para explicar cosas acerca de su salud?

MUJER: Pues no muchos, son raros y ocasionales, por ejemplo hoy he venido dos veces la primera vez dilate dos horas nunca pude pasar porque se da el caso de que el medico atiende a las embarazadas y están dilatan más que la atiendan que la mide, la pesan y eso hace que el médico en ocasiones atienda apresuradamente pero por decir ahorita solo hay una ósea faltaron dos no sé si están de vacaciones o que y eso en parte afecta en manera general al paciente porque uno se desespera y la gente enojada si uno quiere entrar a preguntar algo y no dejan porque se preocupan ya que todos nos queremos ir.

Title: Mantica-1.docx

Descriptor Info: Age: Interview Location: Residence: Interviewer: Date of Interview:

Codes Applied: Care

Excerpt Package: 2162

Excerpt End: 2594

Sara: Ha tenido una prueba de Papanicolaou?

Mujer: Si, cuando estuve embarazada del chiquito y gracias a dios han salido bien, todo

Sara: Ha recibido cuando no ha estado embarazada

Mujer: Ah Sí, también

Sara: Y donde ha recibido?

Mujer: En el centro de salud Benjamín creo que se llama el centro de salud de aquí cerca

Sara: Y quien realizo la prueba?

Mujer: La enfermera, se llama Martita el apellido no lo sé pero así sé que llama

Title: Poneloya-2.docx

Descriptor Info: Age: Interview Location: Residence: Interviewer: Date of Interview:

Codes Applied: Care Confidence

Excerpt Package: 1492

Excerpt End: 2024

ALEXA: Y piensa usted que hay confianza con su información aquí en los centros de salud?

MUJER: Yo pienso que al menos para hacerme el pap como mujer, es con ella, con Aurorita porque me siento bien con ella pero hace un año me mandaron a hacer un estudio con respecto del pap, allá en el centro de salud pero fue con otra mujer con la que me siento en confianza con Mayrita la Licenciada, entonces si hay otros no, porque solo son esas dos personas con quienes yo me siento bien a que me hagan lo que me tienen que hacer como mujer

Title: Perla-3.docx

Descriptor Info: Age: Interview Location: Residence: Interviewer: Date of Interview:

Codes Applied: Care Fear

Excerpt Package: 3713

Excerpt End: 4431

HANNAH: Si y como piensa es la actitud en general de las mujeres acerca de las pruebas de Papanicolaou?

MUJER: A muchas les gusta porque quieren tener salud porque salud es vida pero a otras no porque les da pena, porque dicen ahí que el doctor me va a revisar y eso no me gusta.

HANNAH: Porque es una cosa privada?

MUJER: Si pero pienso yo que en salud se debe de dejar la vergüenza porque te interesa tener tu salud y tu vida sana, entonces ni modo, aunque sea el doctor el que me vaya a revisar yo me dejo aunque me de vergüenza, aunque la cara la ponga a un lado pero yo eso pienso porque me ha tocado que hay doctores que toca que me revisen y aunque me de vergüenza tengo que hacerlo porque me interesa mi salud.

Title: Perla-3.docx

Descriptor Info: Age: Interview Location: Residence: Interviewer: Date of Interview:

Codes Applied: Care Intimacy Fear

Excerpt Package: 3713

Excerpt End: 4431

HANNAH: Si y como piensa es la actitud en general de las mujeres acerca de las pruebas de Papanicolaou?

MUJER: A muchas les gusta porque quieren tener salud porque salud es vida pero a otras no porque les da pena, porque dicen ahí que el doctor me va a revisar y eso no me gusta.

HANNAH: Porque es una cosa privada?

MUJER: Si pero pienso yo que en salud se debe de dejar la vergüenza porque te interesa tener tu salud y tu vida sana, entonces ni modo, aunque sea el doctor el que me vaya a revisar yo me dejo aunque me de vergüenza, aunque la cara la ponga a un lado pero yo eso pienso porque me ha tocado que hay doctores que toca que me revisen y aunque me de vergüenza tengo que hacerlo porque me interesa mi salud.

Title: Mantica-4.docx

Descriptor Info: Age: Interview Location: Residence: Interviewer: Date of Interview:

Codes Applied: Care Pap smear

Excerpt Package: 606

Excerpt End: 828

HANNAH: Y hace cuanto supo que tiene cáncer?

MUJER: En el año 2014

HANNAH: Por medio de qué medida se dio cuenta por una prueba de Papanicolaou?

MUJER: Si Por el Papanicolaou me di cuenta que había un problema en la matriz

Title: Perla-3.docx

Descriptor Info: Age: Interview Location: Residence: Interviewer: Date of Interview:

Codes Applied: Care Pap smear

Excerpt Package: 10115

Excerpt End: 10442

HANNAH: Y antes de su diagnóstico ella había recibido pruebas de pap periódicamente o con qué frecuencia?

MUJER: Uhm, yo creo que no, antes de que le pasara eso yo creo que no se hacía, paso un tiempo en que ella no se lo hacía entonces me imagino que por eso no se daba cuenta y resulto eso cuando ella se sintió los síntomas.

**Cervical Cancer**

Title: Mantica-5.docx

Descriptor Info: Age: Interview Location: Residence: Interviewer: Date of Interview:

Codes Applied: Cervical cancer

Excerpt Package: 3627

Excerpt End: 3945

HANNAH: Piensa que el cáncer de cuello uterino es algo muy común que puede afectarle en un futuro o es algo muy raro?

MUJER: Yo digo que es algo muy grave porque no nos cuidamos, digo no nos cuidamos porque yo no estoy pendiente de mis exámenes, a veces digo hoy no me lo hare será hasta el otro mes y ahí no me cuido.

Title: Mantica-6.docx

Descriptor Info: Age: Interview Location: Residence: Interviewer: Date of Interview:

Codes Applied: Cervical cancer

Excerpt Package: 4961

Excerpt End: 5351

ALEXANDRA: Y se puede morir del cáncer cervical?

MUJER: Si no se ve a tiempo yo diría que si por que tienen que estar con tratamiento en frecuencia

ALEXANDRA: Usted ha tenido contacto directo con la enfermedad por ejemplo alguien que haya sufrido de la enfermedad, un amigo, familiar o conocido

MUJER: No, no me ha tocado vivir cercano a alguien eso Gracias a Dios, todavía no sé qué pasara

Title: Mantica-5.docx

Descriptor Info: Age: Interview Location: Residence: Interviewer: Date of Interview:

Codes Applied: Cervical cancer

Excerpt Package: 5446

Excerpt End: 5642

HANNAH: Ha recibido educación sexual acerca del cáncer de cuello uterino?

MUJER: Si, aquí

HANNAH: Aquí en el centro de Salud pero no en el colegio?

MUJER: No, hace tiempo que deje de ir al colegio

Title: Mantica-5.docx

Descriptor Info: Age: Interview Location: Residence: Interviewer: Date of Interview:

Codes Applied: Cervical cancer

Excerpt Package: 1940

Excerpt End: 3474

HANNAH: usted ha tenido una experiencia con el cáncer de cuello uterino, alguien de su familia o algún amigo que lo haya sufrido?

MUJER: Un conocido, en mi familia Gracias a Dios no, nadie lo ha tenido

HANNAH: Conocido en su familia

MUJER: No, era alguien del Reparto

HANNAH: Ha oído las experiencias de su amiga que ha sufrido de cáncer

MUJER: SI

HANNAH: Como fue su experiencia?

MUJER: Pues fue muy triste porque ella murió a causa de eso, ya que era muy tarde cuando se lo descubrieron, en la Iglesia también a una muchacha le quitaron un pecho y le sacaron y Gracias a Dios ella sobrevivió, pero la que estaba más cerca de mi casa, murió.

HANNAH: Pero ella estaba cómoda hablando acerca de su tratamiento?

MUJER: No porque cuando a ella se lo descubrieron ya no había nada que hacer, todo fue muy tarde.

HANNAH: Y porque piensa que es una cosa muy privada?

MUJER: No sé, pero yo digo que tenemos que hablar para que así estos temas no afecten a otras mujeres

HANNAH: Y su amiga es saludable hoy día o tiene el tratamiento ahora?

MUJER: Ella murió

HANNAH: Oh murió, lo siento.

MUJER: Ya no había nada que hacer cuando se lo descubrieron ya estaba más avanzado.

HANNAH: OH, más avanzado, ok, y como ella descubrió que tenía el cáncer de cuello uterino?

MUJER: Se hizo un Pap y ya no había nada que hacer.

HANNAH: Piensa que ella recibía un pap irregularmente o muchas veces al año?

MUJER: Yo digo que era muy irregular, ella no se hacia

HANNAH: Oh, irregular, lo siento mucho y cuantos años tenía?

MUJER: Tenia treinta y seis años

Title: Subtiava-2.docx

Descriptor Info: Age: Interview Location: Residence: Interviewer: Date of Interview:

Codes Applied: Cervical cancer

Excerpt Package: 3873

Excerpt End: 5676

HANNAH: Ha oído sobre el cáncer de cuello uterino alguna vez?

MUJER: Si más o menos

HANNAH: El cáncer de la matriz es el mismo, puede hablar acerca de esta enfermedad?

MUJER: Bueno de eso casi no tengo información, solo sé que por medio del pap puedo saber si tengo algún tipo de cáncer pero eso casi no lo manejo.

HANNAH: Ha tenido alguna experiencia con el cáncer, con alguien de su familia, amigos o alguien de la comunidad?

MUJER: No, ahorita no, Gracias a Dios estamos sanos y libres de cánceres todos.

HANNAH: Bueno, y piensa que es una enfermedad común en las mujeres?

MUJER: Bueno a veces he escuchado que hay mujeres que como no se hacen las pruebas del pap y se les detecta el cáncer cuando ya es demasiado tarde y a veces hasta se mueren las pobres, entonces es algo muy importante porque el Papanicolaou te detecta cualquier tipo de enfermedad que se puede combatir en el momento pero cuando está muy avanzada ya no habría nada que hacer entonces por eso creo que es algo muy importante por eso hay que visitar el centro de salud y hacerse todas las pruebas que sean necesarias para poder estar sana, aunque a veces uno cree estar bien pero por dentro no lo está y la enfermedad puede ir avanzando silenciosamente por eso es bueno chequearse

HANNAH: Como es el conocimiento de las mujeres de la comunidad acerca de esta enfermedad?

MUJER: Por lo menos cada vez que yo vengo aquí ya sea por mi o con mi hijo, ellos me preguntan si ya me hice el pap si ya me chequee que andas y asi uno se va dando cuenta poco a poco de lo que significa todo, desde los exámenes que te haces y todo lo demás

HANNAH: Cuál piensa es la actitud acerca del cáncer de cuello uterino?

MUJER: Bueno la verdad es que no se, como no lo he tenido pero creo que si uno le pide ayuda a Dios le ayudara a sanar pero no se

Title: Mantica-3.docx

Descriptor Info: Age: Interview Location: Residence: Interviewer: Date of Interview:

Codes Applied: Cervical cancer

Excerpt Package: 4006

Excerpt End: 4373

HANNAH: Y piensa que usted tiene la información suficiente sobre el cáncer de cuello uterino y cosas que puede hacer para prevenir el cáncer?

MUJER: Yo pienso que si porque nos han dado varias orientaciones y me he dado cuenta de cosa en otros lugares y también lo que dicen aquí los médicos por qué inician eso y además de las indicaciones que dan para disminuir eso

Title: Perla-2.docx

Descriptor Info: Age: Interview Location: Residence: Interviewer: Date of Interview:

Codes Applied: Cervical cancer

Excerpt Package: 4966

Excerpt End: 5440

ALEXANDRA; Es siempre una enfermedad terminal o hay un tratamiento que pueda prevenir la muerte?

MUJER: Si yo estuve con una señora que le sacaron la matriz y ella está bien ahora

ALEXANDRA: Y con esa cirugía hay muchos riesgos, hay un tabú o es una cirugía muy buena?

MUJER: No sé, porque a mi mamá no se la hicieron

ALEXANDRA: Y porque cree usted que a su mama no se la hicieron?

MUJER: Al parecer ya estaba muy avanzado y también dice que mi Papa no quiso que la operaran

Title: Perla-3.docx

Descriptor Info: Age: Interview Location: Residence: Interviewer: Date of Interview:

Codes Applied: Cervical cancer

Excerpt Package: 7277

Excerpt End: 8911

HANNAH: Y piensa usted que hay una relación entre el virus de papiloma humano y el cáncer de cuello uterino? Ha oído del cáncer de cuello uterino?

MUJER: Yo pienso que es la misma cosa, es que me imagino que el papiloma humano tiene que ir relacionado con el cáncer uterino porque es lo mismo por eso pienso no hay diferencia

HANNAH: Y ha tenido una experiencia con el cáncer de cuello uterino, sabe de una persona que ha sufrido de cáncer?

MUJER: Mi mama.

HANNAH: Su madre? Oh lo siento mucho

MUJER: Si a ella le hicieron una cono biopsia es en el cuello, y después de eso le dieron tratamiento para ver qué pasaba y ella era una ama de casa que no salía a ningún lado pero resulta que mi Papa fue el de la culpa, como el salía y le gustaba andar con muchas mujeres.

HANNAH: Y ella recibe o recibió tratamiento?

MUJER: Si, a ella después de ese examen que le hicieron recibió tratamiento y después del tratamiento le dijo el doctor que el tratamiento era caro, que le podía ayudar a disminuir pero le voy a decir algo es más recomendable que se saque la matriz porque ya no le sirve la matriz, así que decida usted si se la saca o no, de todas formas ya no le sirve para nada, y entonces ella decidió sacársela porque si ya estaba avanzando.

HANNAH: Entonces se la quería sacar en forma de una cirugía?

MUJER: Si, y ella acepto porque dijo era preferible que se la sacaran porque sabía que era algo que no le servía y le podía causar problemas entonces ella prefirió que se la sacaran, nada más que ella sigue viniendo a sus citas con el ginecólogo

HANNAH: Y qué pasó después de la operación?

MUJER: Nada, ella sigue su vida normal

Title: Santa Ana-3.docx

Descriptor Info: Age: Interview Location: Residence: Interviewer: Date of Interview:

Codes Applied: Cervical cancer

Excerpt Package: 2461

Excerpt End: 3349

HANNAH: Usted tiene una experiencia de alguien que tiene cáncer de cuello uterino, un miembro de su familia, amigos?

MUJER: Si, con alguien de mi familia, y ya falleció una de cáncer de cuello uterino por eso es que yo invito a muchas personas a que se revisen a las mujeres

HANNAH: Un miembro cercano de su familia, como tía?

MUJER: Si, era una tía.

HANNAH: Lo siento, puede hablar un poco de su experiencia o de la experiencia de su tía?

MUJER: Bueno cuando mi tía sufrió esa enfermedad del cáncer de cuello uterino, ella era tan hermosa y después se puso tan delgadita, le hicieron quimioterapias, pero ella ya se lo detectaron muy avanzado ya que ella no acostumbraba a hacerse el pap entonces se lo diagnosticaron ya muy avanzado, las quimioterapias ya no le ayudaban porque el cáncer ya estaba en un término entonces se combatió pero no se curó y son muy dolorosos los tratamientos.

Title: SantaAna-4.docx.docx

Descriptor Info: Age: Interview Location: Residence: Interviewer: Date of Interview:

Codes Applied: Cervical cancer

Excerpt Package: 4671

Excerpt End: 4988

ALEXANDRA: Pero y del cáncer de cuello uterino?

MUJER: Si, ese si lo he oído

ALEXANDRA: Es común aquí?

MUJER: Bueno, si aquí nos dicen de eso y todo, que cuando nos estemos bañando nos toquemos algunas partes y así sabremos algo porque yo digo que a mis hijos no los voy a dejar abandonados porque ellos me necesitan.

Title: Mantica-3.docx

Descriptor Info: Age: Interview Location: Residence: Interviewer: Date of Interview:

Codes Applied: Cervical cancer

Excerpt Package: 87

Excerpt End: 796

HANNAH: Y puede decirme su experiencia con el cuidado de salud

MUJER: Pues ha sido bueno, pero en los últimos años he estado un poco enferma ya que es hereditario el problema de cáncer porque mi mama murió de cáncer y ahora nosotras sus hijas parece que estamos padeciendo eso, porque actualmente mi problema es ese

HANNAH: Y de qué tipo de cáncer murió?

MUJER: Cáncer en el cuello de la matriz

HANNAH: Lo siento mucho, como afecta esto en su vida y en su cuidado?

MUJER: Pues afecta mucho ya que a veces nosotras tenemos vuestros hijos pequeños y hay que estar en tratamiento y cuidado y a veces hay cáncer bueno y hay cáncer malo, a veces uno tiene que estar tomando mucho tratamiento para vivir un poco mas

Title: Perla-4.docx

Descriptor Info: Age: Interview Location: Residence: Interviewer: Date of Interview:

Codes Applied: Cervical cancer

Excerpt Package: 1370

Excerpt End: 2103

HANNAH: Bueno, y usted sabe acerca del cáncer de cuello uterino?

MUJER: Si

HANNAH: Y que sabe acerca de este cáncer?

MUJER: No pues, a más, solamente que si ese virus se prolonga se puede convertir en cáncer y que por eso hay que estar en constante chequeo y seguir el tratamiento que indican

HANNAH: Y cual piensa que es el conocimiento y actitud de las personas acerca del cáncer de cuello uterino en la comunidad?

MUJER: Eso es transmitido, que es una enfermedad de transmisión sexual

HANNAH :El cáncer?

MUJER: Se refiere a eso la pregunta?

HANNAH: Sí, pero usted cree que muchas personas en la comunidad saben acerca de este virus y el cáncer?

MUJER: Yo diría que sí porque ahora con bastantes charlas que se dan en los centros.

Title: Los Leches-3.docx

Descriptor Info: Age: Interview Location: Residence: Interviewer: Date of Interview:

Codes Applied: Cervical cancer

Excerpt Package: 3945

Excerpt End: 4602

SARA: Ha oído de cáncer de cuello uterino sabe de alguien que tiene esa enfermedad?

MUJER: Mi prima pero es mujer

SARA: Oh y cuál fue su experiencia que ocurrió?

MUJER: Pues la verdad aquí fue la doctora la que se lo descubrió, con el examen del pap que le miraron algo raro y hasta la fecha ella se está chequeando pero ella se ha visto bien mal

SARA: Fue al hospital por tratamiento?

MUJER: Si, si fue

SARA: Y ella habla con su familia y su pareja sobre la enfermedad?

MUJER: Si

SARA: Si puede cambiar algo en la experiencia o tratamiento de ella que cambiaria?

MUJER: Claro que si porque la veo complicada y de eso le han venido muchas enfermedades ahora

Title: Mantica-3.docx

Descriptor Info: Age: Interview Location: Residence: Interviewer: Date of Interview:

Codes Applied: Cervical cancer

Excerpt Package: 2002

Excerpt End: 2274

HANNAH: Lo siento mucho es muy difícil eso, piensa que es una enfermedad que puede afectarle en el futuro?

MUJER: Pues yo creo que no, porque si uno toma el tratamiento a como lo manda el médico y sigue indicaciones, tal vez no habrá ningún problema espero en Dios que no

Title: Perla-4.docx

Descriptor Info: Age: Interview Location: Residence: Interviewer: Date of Interview:

Codes Applied: Cervical cancer

Excerpt Package: 3004

Excerpt End: 4853

HANNAH: Usted sabe de alguien cercano ya sea de su familia o amigos que hayan sufrido el cáncer de cuello uterino?

MUJER: Miembro de mi familia no, pero si conocido

HANNAH: Una amiga?

MUJER: Si

HANNAH: Y que paso con su amiga?

MUJER: Pues una de ellas ya murió, la otra tal vez no está sana pero lo supero

HANNAH: Y puede hablar un poco de la experiencia de su amiga?

MUJER: Bueno es que primero a esa amiga le resulto que le amputaron un busto y el cáncer se le fue desarrollando y se le hizo un acceso en la espalda y así le vino el cáncer de cuello uterino después en ese instante le sacaron su matriz y todo después el cáncer su terminal fue en el cuello se le vino ahí y se inflamo se hincho no se podía asolear porque se inflamaba y tenía que caminar vendada porque le daba pena y se le rego en todo su cuerpo por decirlo así

HANNAH: Y como encontró ella su cáncer? por medio de una prueba de pap o cómo?

MUJER: No, es que primero lo de ella fue aquí (busto) y después con el tiempo se fue chequeando más y resulto eso

HANNAH: Como fue el tratamiento de su amiga?

MUJER: Bueno fue muy duro porque tuvo que viajar hasta Costa Rica y boto el cabello y todo fue muy duro

HANNAH: Ella fue a Costa Rica para recibir tratamiento?

MUJER: Si, unos padres de la Iglesia Católica donde ella trabajaba, ellos le ayudaron de esa manera porque como ella no tenía muchos recursos para viajar entonces a través de una organización que creo es la Ortiz Guardián así ella pudo viajar

HANNAH: Viajar para recibir más recursos sobre medicina?

MUJER: Así es correcto, y así ella vivió muchos años con esa enfermedad porque ella tuvo alrededor de trece años desde que le inicio la enfermedad es decir que desde que le inicio el problema de busto, después la espalda, luego la matriz y por último el cuello

HANNAH: Pero ella murió a causa de su cáncer?

MUJER: Si

Title: Perla-3.docx

Descriptor Info: Age: Interview Location: Residence: Interviewer: Date of Interview:

Codes Applied: Cervical cancer

Excerpt Package: 7277

Excerpt End: 8911

HANNAH: Y piensa usted que hay una relación entre el virus de papiloma humano y el cáncer de cuello uterino? Ha oído del cáncer de cuello uterino?

MUJER: Yo pienso que es la misma cosa, es que me imagino que el papiloma humano tiene que ir relacionado con el cáncer uterino porque es lo mismo por eso pienso no hay diferencia

HANNAH: Y ha tenido una experiencia con el cáncer de cuello uterino, sabe de una persona que ha sufrido de cáncer?

MUJER: Mi mama.

HANNAH: Su madre? Oh lo siento mucho

MUJER: Si a ella le hicieron una cono biopsia es en el cuello, y después de eso le dieron tratamiento para ver qué pasaba y ella era una ama de casa que no salía a ningún lado pero resulta que mi Papa fue el de la culpa, como el salía y le gustaba andar con muchas mujeres.

HANNAH: Y ella recibe o recibió tratamiento?

MUJER: Si, a ella después de ese examen que le hicieron recibió tratamiento y después del tratamiento le dijo el doctor que el tratamiento era caro, que le podía ayudar a disminuir pero le voy a decir algo es más recomendable que se saque la matriz porque ya no le sirve la matriz, así que decida usted si se la saca o no, de todas formas ya no le sirve para nada, y entonces ella decidió sacársela porque si ya estaba avanzando.

HANNAH: Entonces se la quería sacar en forma de una cirugía?

MUJER: Si, y ella acepto porque dijo era preferible que se la sacaran porque sabía que era algo que no le servía y le podía causar problemas entonces ella prefirió que se la sacaran, nada más que ella sigue viniendo a sus citas con el ginecólogo

HANNAH: Y qué pasó después de la operación?

MUJER: Nada, ella sigue su vida normal

Title: Subtiava-1.docx

Descriptor Info: Age: Interview Location: Residence: Interviewer: Date of Interview:

Codes Applied: Cervical cancer

Excerpt Package: 9037

Excerpt End: 9900

HANNAH: Y usted ha oído sobre el cáncer de cuello uterino?

MUJER: Si he oído de ese cáncer de cuello uterino pero no sé en si porque dicen que se le hacen unas llagas por dentro a la mujer en el cuello y después se va dañando, incluso una hermana mía ha tenido un problema similar fue como en el dos mil diez o dos mil once.

HANNAH: Ha sufrido de cáncer de cuello uterino su hermana?

MUJER: Yo digo que sí, porque ella dice que la doctora le ha dicho y le ha mandado tratamiento y todo, y se le ha compuesto, incluso en estos días ella anduvo donde la doctora y le pregunto a otra doctora nueva y ella le dijo que va a vivir con eso toda la vida, además el caso es como que el útero se le ha puesto chiquito y eso debe de ser porque ya le va a venir la menopausia pero ella es menor que yo todavía entonces digo yo que no es posible porque está muy joven todavía.

Title: Perla-5.docx

Descriptor Info: Age: Interview Location: Residence: Interviewer: Date of Interview:

Codes Applied: Cervical cancer

Excerpt Package: 7810

Excerpt End: 9745

ALEXA: Ah que bien y cuando escucha la palabra cáncer de cuello uterino o cáncer cervical en que piensa? Oh las ideas que tiene con el cáncer de cuello uterino?

MUJER: Bueno, la información que tengo sobre cáncer uterino pues la verdad que en conocimiento científico especifico es poco pero así a nivel empírico puedo decir que lo primero que se me viene a la mente es que es alguna enfermedad peligrosa algo que es delicado para toda mujer, algo preocupante cuando eso viene a mi mente pienso en muerte, que tal vez tengo que hacerme un tratamiento fuerte para eso, no recuerdo como se llama eso que le hacen a las personas con cáncer.

ALEXA: Como quimioterapias?

MUJER: Si, eso o que lo operen, que le saquen la matriz y no vuelvan a tener hijos y pues eso es lo que pienso yo y mi conocimiento es que es una enfermedad bien peligrosa

ALEXA: Y ha tenido un familiar o amigo que ha padecido de ello?

MUJER: No, Gracias a Dios no, hasta donde yo conozco no.

ALEXA: Que bien y como es la actitud y el conocimiento de la comunidad acerca de esta enfermedad?

MUJER: Pues no se realmente porque habría que hacer una encuesta a las mujeres que tal vez se han hecho esto, es como lo que está haciendo usted entonces ahí obtiene ese conocimiento y tal vez pude darlo a conocer luego aquí un porcentaje de las mujeres que conocen esa enfermedad y que se puede hacer para que tengan ese conocimiento científico y no empírico, tal vez ese sería un tipo, preguntarle a cada una, a nivel personal eso opino.

ALEXA: Y con quien se sentiría cómoda hablar del cáncer de cuello uterino?

MUJER: Pues a como le digo yo no soy tan cerrada, creo que los varones pueden hacer una buena charla no solo las mujeres, es solo que a veces las mujeres tienen más confianza con las mismas mujeres porque aquí el de ginecología es un doctor, no hay mujeres sino que es un doctor y entonces el papel del doctor tienen que pasar con él aunque no se sientan cómodas.

Title: Los Leches-1 (1).docx

Descriptor Info: Age: Interview Location: Residence: Interviewer: Date of Interview:

Codes Applied: Cervical cancer

Excerpt Package: 5748

Excerpt End: 6114

Sara: Have you experience with cervical cancer? Have you heard about this cancer?

Woman: Yes.

Sara: Yes, and do you know about someone who has this cancer?

Woman: Not here, but yes in other places, like the hospital, when I had my problem I heard about in the hospital.

Sara: And how is it looks the patients?

Woman: well I do not know because I only heard about it.

Title: Santa Ana-3.docx

Descriptor Info: Age: Interview Location: Residence: Interviewer: Date of Interview:

Codes Applied: Cervical cancer

Excerpt Package: 4441

Excerpt End: 4620

HANNAH: Usted piensa que ella estaba muy preocupada acerca de esta enfermedad? Piensa que es algo común?

MUJER: Pensaban que era una enfermedad común que no le tomaban importancia

Title: Los Leches-2.docx

Descriptor Info: Age: Interview Location: Residence: Interviewer: Date of Interview:

Codes Applied: Cervical cancer

Excerpt Package: 3666

Excerpt End: 3932

HANNAH: Y como piensa que es la actitud y conocimiento de las mujeres en la comunidad acerca del cáncer de cuello uterino?

MUJER: Pues lo que yo pienso es que uno debe cuidare de esas cosas estarse chequeando donde uno pueda ya sea en el centro de salud u otro lugar

Title: Los Leches-1 (1).docx

Descriptor Info: Age: Interview Location: Residence: Interviewer: Date of Interview:

Codes Applied: Cervical cancer

Excerpt Package: 6918

Excerpt End: 7587

Sara: And about cervical cancer how is the knowledge and attitude from the community and Nicaraguan women’s about this? Have you see the cervical cancer on talks, TV or Literature?

Woman: I have not seen nearly here or on television.

Sara: How do you take decisions?...Ups, Sorry, I mean, are you going to talk about this with your sons and daughters about these health issues?

Woman: What do you mean? If I ever talk to them about it or if I will talk to them later?

Sara: If you talk about sexual diseases

Woman: No, no, no, I do not think so.

Sara: But you will talk about problems with the pressure, heart or something like that?

Woman: Yes, about that absolutely.

Title: Mantica-2.docx

Descriptor Info: Age: Interview Location: Residence: Interviewer: Date of Interview:

Codes Applied: Cervical cancer

Excerpt Package: 3676

Excerpt End: 4287

ALEXANDRA: Ha oído mucho del cáncer cervical l cáncer de cuello uterino

MUJER: No, casi no he oído de eso

ALEXANDRA: Usted piensa que hay un riesgo para todas las mujeres con esa enfermedad

MUJER: Yo digo que si

ALEXANDRA: Y usted piensa que puede afectarle en el futuro+

MUJER: Yo digo que no se solo dios porque uno se cuida tal vez no, porque a veces eso se da por las relaciones sexuales entonces uno solo tiene que cuidarse, por lo menos mi marido no es santo, cualquier momento puede fallar pero yo le digo a mi marido que si hará algo que use condón

ALEXANDRA: De esa manera se protege

MUJER: Si, así es

Title: Santa Ana-1.docx

Descriptor Info: Age: Interview Location: Residence: Interviewer: Date of Interview:

Codes Applied: Cervical cancer

Excerpt Package: 4124

Excerpt End: 4321

SARA: Tiene experiencia con el cáncer de cuello uterino? Ha oído hablar de esta enfermedad?

MUJER: Si, si la he oído bastante

SARA: Sabe de alguien que tiene cáncer de cuello uterino

MUJER: Pues no

Title: Mantica-6.docx

Descriptor Info: Age: Interview Location: Residence: Interviewer: Date of Interview:

Codes Applied: Cervical cancer

Excerpt Package: 3571

Excerpt End: 4682

ALEXANDRA: Ha escuchado mucho hablar o habla acerca del cáncer cervical o cáncer de cuello uterino?

MUJER: Son pocos casos los que hay porque son pocas personas las que se están constantemente en chequeo pero las que están actualmente siempre están con su tratamiento pero hay personas que no acuden a los centros de salud las rurales principalmente entonces ahí es donde hay que llevar las charlas

ALEXANDRA: Hay mucho riesgo para las mujeres de contraer el cáncer cervical?

MUJER: Probablemente es muy positivo que lo tengan porque no se ven a tiempo y no hacen uso del tratamiento adecuado

ALEXANDRA: Usted cree que le puede afectar en un futuro?

MUJER: Llevando mi control yo creo que no, con mi control al día lo puedo detectar a tiempo y obtengo el tratamiento adecuado

ALEXANDRA: Y si por ejemplo una mujer escucha la palabra cáncer o cáncer de cuello uterino eso qué sentido tiene como reacciona?

MUJER: Es alarmante pero yo creo que sí que si lo detecto a tiempo asistiendo a mi control yo sé que se puede medicar a tiempo pero si ya es demasiado tarde es alarmante ya que las consecuencias son grandes

Title: Los Leches-2.docx

Descriptor Info: Age: Interview Location: Residence: Interviewer: Date of Interview:

Codes Applied: Cervical cancer

Excerpt Package: 3272

Excerpt End: 3494

HANNAH: Ha oído del cáncer de cuello uterino?

MUJER: Si he oído

HANNAH: Puede hablar acerca de este cáncer? Que sabe de este cáncer?

MUJER: Lo único que yo sé es que es un cáncer de la matriz que te va comiendo por dentro

Title: Santa Ana-3.docx

Descriptor Info: Age: Interview Location: Residence: Interviewer: Date of Interview:

Codes Applied: Cervical cancer

Excerpt Package: 700

Excerpt End: 1625

HANNAH: Si, bueno, gracias, dígame un poco acerca de su experiencia con la educación sexual?

MUJER: Bueno la educación de salud sexual está en que por ejemplo que uno se debe de proteger porque hay muchas enfermedades como el virus de papiloma humano y el VIH por eso hay que protegerse y cuidarse porque además no solamente las pueden transmitir por el sexo, también se puede transmitir si una persona que está infectada con VIH se corta y hacen contacto de sangre con sangre se pueden transmitir o una aguja.

HANNAH: Y de dónde aprendió sobre estos temas?

MUJER: Bueno, en los colegios aprendemos, las charlas que va haciendo el centro de salud que las dan constantemente sobre las enfermedades virales como las podemos evitar y que debemos hacer para tener un mejor cuidado

HANNAH: Y la información sobre le vph y el cáncer de cuello uterino lo ha tenido del colegio?

MUJER: No, del centro de salud de las charlas que dan

Title: Perla-5.docx

Descriptor Info: Age: Interview Location: Residence: Interviewer: Date of Interview:

Codes Applied: Cervical cancer

Excerpt Package: 14461

Excerpt End: 14883

ALEXA: Hay algo más que quisiera compartir acerca de la salud de las mujeres?

MUJER: Lo que me pregunto hace rato acerca de cuello uterino sería bueno aprender porque a ciencia cierta no sé cuántas mujeres están informadas al respecto y sería bueno que pongan un mural de una cifra de cuantas personas y mujeres están informadas al respecto porque en realidad yo no lo sé o tal vez en su encuesta poner una gráfica de eso.

Title: Mantica-3.docx

Descriptor Info: Age: Interview Location: Residence: Interviewer: Date of Interview:

Codes Applied: Cervical cancer Attendance

Excerpt Package: 3656

Excerpt End: 4005

HANNAH: Y usted sabe de amigos o familia que también ha sufrido de cáncer de cuello uterino?

MUJER: Pues yo creo que es hereditario a como le digo porque tengo dos hermanas que tienen eso, ellas vienen aquí al Mantica a hacerse sus exámenes y a tomar su tratamiento porque aquí se lo dan, yo estoy empezando en eso, y espero en Dios no sea nada malo

Title: Santa Ana-2.docx

Descriptor Info: Age: Interview Location: Residence: Interviewer: Date of Interview:

Codes Applied: Cervical cancer Fear

Excerpt Package: 5792

Excerpt End: 6070

ALEXANDRA; Y cuando escucha sobre el cáncer de cuello uterino que piensa?

MUJER: Bueno yo digo que es una enfermedad mortal que yo no sé la deseo a nadie porque por lo menos aquí en la comunidad ya ha habido casos de personas que mueren de cáncer y no solo uno si no que varios

Title: Perla-2.docx

Descriptor Info: Age: Interview Location: Residence: Interviewer: Date of Interview:

Codes Applied: Cervical cancer HPV

Excerpt Package: 6148

Excerpt End: 6397

ALEXANDRA: De donde recibe información acerca de esas enfermedades?

MUJER: Solo escuche a mi mama y ahora pues que me dicen de mi hija

ALEXANDRA: Y por otros medios de los cuales recibe información?

MUJER: Bueno, de la tele ahí dan mucha información

Title: Mantica-4.docx

Descriptor Info: Age: Interview Location: Residence: Interviewer: Date of Interview:

Codes Applied: Cervical cancer HPV

Excerpt Package: 829

Excerpt End: 1390

HANNAH: Y que dijo su médico acerca del cáncer en ese momento?

MUJER: Bueno me atienden en el hospital OSCAR DANILO ROSALES primero me hacían las quimioterapias pero como eso no funciono, tuvieron que hacerme un cono para cortar parte de la matriz y ver si así se detiene bueno actualmente no siento nada estoy siempre con mis consultas y Papanicolaou y viendo hasta donde esta y si se ha detenido y mi problema se llama papiloma humano pero supuestamente con el cono me que hicieron verían si se detenía entonces eso es lo que estamos esperando a ver qué pasa

Title: Mantica-1.docx

Descriptor Info: Age: Interview Location: Residence: Interviewer: Date of Interview:

Codes Applied: Cervical cancer Pap smear

Excerpt Package: 6362

Excerpt End: 7329

ara: Tiene experiencia con el cáncer de cuello uterino, sabe de alguien que tiene esta enfermedad o ha oído d esta enfermedad?

Mujer: No, he visto en la televisión en las charlas pero nunca he sabido de alguien que se ha muerto

Sara: Como percibe de la televisión? Como fue la experiencia en la televisión?

Mujer: Fue por medio de las charlas que también dan, he leído revistas, en los periódicos, que es un cáncer que se dan cuenta por medio del pap, que a veces no lo encuentran a tiempo y que el cáncer se les va avanzando, lo he leído pero no he visto en persona

Sara: Que piensa cuando oye la palabra cáncer?

Mujer: Que es horrible eso

Sara: Como es el conocimiento y la actitud de la comunidad acerca del cáncer de cuello uterino?

Mujer: Nunca he escuchado de eso, pero yo miro que hay muchas mujeres que se cuidan, porque veo a los estudiantes que andan dejando los resultados de las pruebas de pap en las casas y todo eso, pero nunca he visto mujeres enfermas

Title: Poneloya-1.docx

Descriptor Info: Age: Interview Location: Residence: Interviewer: Date of Interview:

Codes Applied: Cervical cancer Private/taboo

Excerpt Package: 3118

Excerpt End: 3663

ALEXANDRA: El cáncer cervical o de cuello uterino tiene una relación con el virus de papiloma humano?

MUJER: La verdad eso no lo sé.

ALEXANDRA: El cáncer cervical es común en la comunidad?

MUJER: No sé, lo más común que yo escucho es la cresta de gallo que es una enfermedad de trasmisión sexual y el SIDA pero es lo que yo escucho que es lo más común pero no es que yo sepa que alguien lo tenga, porque a como dije eso es algo secreto entre el paciente y el doctor o enfermera eso es algo que no se puede divulgar al menos que uno mismo lo diga

Title: Perla-2.docx

Descriptor Info: Age: Interview Location: Residence: Interviewer: Date of Interview:

Codes Applied: Cervical cancer Support

Excerpt Package: 2153

Excerpt End: 2773

ALEXANDRA: Y cree usted que las chicas adolescentes y los jóvenes reciben suficiente información acerca de su salud?

MUJER: No porque al menos ahorita mi hija está preguntándome que es eso que le harán, porque es una biopsia entonces yo le digo que ni idea, que no sé, pero es para no asustarla pero mi mama murió de cáncer y yo no quiero asustarla a ella entonces yo le digo que no sé qué le harán pero es solo para no asustarla aunque yo ya más o menos se.

ALEXANDRA: Su mama de qué tipo de cáncer murió?

MUJER: De cáncer en la matriz

ALEXANDRA: Y ella le dijo a usted de que estaba sufriendo de ese cáncer?

MUJER: Si

**Confidence**

Title: Perla-2.docx

Descriptor Info: Age: Interview Location: Residence: Interviewer: Date of Interview:

Codes Applied: Confidence

Excerpt Package: 3232

Excerpt End: 3426

ALEXANDRA: Tiene confianza de que las pruebas de Pap dan información precisa sobre su salud?

MUJER: Que si es confiable?

ALEXANDRA: Si, que si dan información importante?

MUJER: Si nos explican

Title: Perla-3.docx

Descriptor Info: Age: Interview Location: Residence: Interviewer: Date of Interview:

Codes Applied: Confidence

Excerpt Package: 4942

Excerpt End: 5104

HANNAH: Y piensa usted que los resultados de su prueba son seguros en el centro? O tiene dudas sobre eso?

MUJER: Si, un noventa por ciento probables que sea buena

Title: Mantica-2.docx

Descriptor Info: Age: Interview Location: Residence: Interviewer: Date of Interview:

Codes Applied: Confidence

Excerpt Package: 7018

Excerpt End: 8017

ALEXANDRA: Y de donde prefiere recibir información acerca de su salud de los médicos o de cuales medios

MUJER: De los médicos porque son los que saben y son puestos por Dios y si alguien más sabe que de información

ALEXANDRA: Y tiene mucha confianza en lo que digan los médicos

MUJER: En algunos si porque a veces los doctores no se, por ejemplo en mi caso yo tengo cinco meses de que perdí un bebe y me confié porque el doctor me decía que estaba bien él bebe, porque yo le decía que no se movía y él me dijo que todo estaba bien pero a los ocho días otra doctora me miro y me dijo que él bebe estaba muerto entonces algunos saben y lo dicen todo pero otros no, pero eso me paso

ALEXANDRA: Y con otros exámenes ´por ejemplo la prueba de pap tiene confianza en que da información precisa acerca de su salud

MUJER: Yo dos veces me he hecho el pap y me sale lo que debe de ser, algunas dicen que en privado es hacerse mejor la prueba de pap pero yo digo que es igual porque todos pasan por el hospital

Title: Perla-5.docx

Descriptor Info: Age: Interview Location: Residence: Interviewer: Date of Interview:

Codes Applied: Confidence

Excerpt Package: 7489

Excerpt End: 7809

ALEXA: Y cuanta confianza tiene en que las pruebas de Pap dan información precisa acerca de su salud?

MUJER: Bueno, esa es una buena pregunta, yo me la he hecho dos veces y el año pasado me la hice dos veces y pues los resultados han sido los mismos entonces tal vez ahí hay algo positivo con la pregunta, es algo fiable

Title: Mantica-1.docx

Descriptor Info: Age: Interview Location: Residence: Interviewer: Date of Interview:

Codes Applied: Confidence

Excerpt Package: 3201

Excerpt End: 3553

Sara: Tiene la confianza que la prueba de pap dan información precisa sobre su salud?

Mujer: Como? Que salga sin enfermedades?

Sara: Si, si la prueba fue correcta y puede decir si tiene una enfermedad o no?

Mujer: Bueno, como, no le entiendo

Sara: Tiene confianza?

Mujer: Ah, Que salga negativo

Sara: Si, que la prueba es correcta

Mujer: Ah, bueno, si

Title: Santa Ana-1.docx

Descriptor Info: Age: Interview Location: Residence: Interviewer: Date of Interview:

Codes Applied: Confidence

Excerpt Package: 3034

Excerpt End: 3145

SARA: Tiene confianza que las pruebas de pap dan información precisa de su salud

MUJER: Sí, hay mucha confianza

Title: Santa Ana-2.docx

Descriptor Info: Age: Interview Location: Residence: Interviewer: Date of Interview:

Codes Applied: Confidence

Excerpt Package: 4869

Excerpt End: 5129

ALEXANDRA: Cuanta confianza tiene de que las pruebas de pap dan información precisa sobre su salud?

MUJER: Bueno yo ya me lo he hecho y pues Gracias a Dios nunca me ha salido nada malo y pues ahorita me daré cuenta de que tengo porque no sé pero es importante.

Title: Perla-3.docx

Descriptor Info: Age: Interview Location: Residence: Interviewer: Date of Interview:

Codes Applied: Confidence

Excerpt Package: 4942

Excerpt End: 5104

HANNAH: Y piensa usted que los resultados de su prueba son seguros en el centro? O tiene dudas sobre eso?

MUJER: Si, un noventa por ciento probables que sea buena

Title: Mantica-6.docx

Descriptor Info: Age: Interview Location: Residence: Interviewer: Date of Interview:

Codes Applied: Confidence

Excerpt Package: 7808

Excerpt End: 8155

ALEXANDRA: Y usted tiene confianza en los médicos, por ejemplo tiene confianza en sí las pruebas de pap dan información precisa sobre su salud?

MUJER: Yo diría que si porque es el único medio que acudimos y nos da una respuesta y es con la respuesta que nos quedamos pero segura al cien por ciento no estoy de que si alguien regula esos resultados

Title: SantaAna-4.docx.docx

Descriptor Info: Age: Interview Location: Residence: Interviewer: Date of Interview:

Codes Applied: Confidence

Excerpt Package: 4342

Excerpt End: 4590

ALEXANDRA: Y cuanta confianza tiene de que las pruebas de pap dan información precisa acerca de su salud?

MUJER: Bueno yo tengo bastantes años de venir a este centro desde que vivo aquí y siempre vengo con mis hijos y yo también me hago todo aquí

Title: Los Leches-3.docx

Descriptor Info: Age: Interview Location: Residence: Interviewer: Date of Interview:

Codes Applied: Confidence

Excerpt Package: 2792

Excerpt End: 2900

SARA: Tiene la confianza que las pruebas de papa dan información precisa sobre su salud?

MUJER: Si, si tengo

**Confidentiality**

Title: Los Leches-2.docx

Descriptor Info: Age: Interview Location: Residence: Interviewer: Date of Interview:

Codes Applied: Confidentiality

Excerpt Package: 2306

Excerpt End: 2443

HANNAH: Y piensa que los resultados son seguros en los puestos de salud?

MUJER: Si porque me los he hecho en oros lados y me salen igual

Title: Santa Ana-2.docx

Descriptor Info: Age: Interview Location: Residence: Interviewer: Date of Interview:

Codes Applied: Confidentiality

Excerpt Package: 1425

Excerpt End: 1783

ALEXANDRA: Y piensa que hay confianza con su información aquí en los centros de salud?

MUJER: Bueno, yo siempre que he venido aquí le he dicho cosas a la doctora y Gracias a Dios nunca ha salido a luz y si algún día sale yo diría que me enojaría y los demandaría porque es un centro de salud y todo lo que se hagan aquí debe ser privado solo entre ella y yo.

Title: Perla-5.docx

Descriptor Info: Age: Interview Location: Residence: Interviewer: Date of Interview:

Codes Applied: Confidentiality

Excerpt Package: 881

Excerpt End: 1131

ALEXA: Y cree que hay confianza con la privacidad de su información en los centros?

MUJER: Si, eso sí, sino que hay que mejorar a nivel de las salas tal vez necesitan ellos ampliar pero dada la situación que aquí es pequeño uno se tiene que acomodar.

Title: Subtiava-1.docx

Descriptor Info: Age: Interview Location: Residence: Interviewer: Date of Interview:

Codes Applied: Confidentiality Promiscuity

Excerpt Package: 13577

Excerpt End: 14404

HANNAH: Y entonces que dijeron los doctores?

MUJER: Porque cuando ellos están en las consultas le están dando el diagnostico o dando un tratamiento ellos hablan duro y se escucha afuera y todo mundo se da cuenta del problema de salud que está pasando el paciente.

HANNAH: Oh es porque los doctores hablan de los pacientes delante de otros pacientes?

MUJER: Si, así es

HANNAH: Entonces por eso usted cree que los resultados y diagnósticos de los exámenes no son muy seguros?

MUJER: Ellos andan hablando y todo y eso no está bien pienso yo.

HANNAH: Piensa que es una forma de discriminación?

MUJER: Si, así mismo

HANNAH: Y porque piensa que puede causar discriminación esta enfermedad?

MUJER: Porque todos dicen que ella es una persona vaga y que pudo haberse contagiado de eso por vagancia de ella con otro hombre, me entiende?

**Experiences With Pap**

Title: Mantica-1.docx

Descriptor Info: Age: Interview Location: Residence: Interviewer: Date of Interview:

Codes Applied: Experiences with Pap

Excerpt Package: 2162

Excerpt End: 2594

Sara: Ha tenido una prueba de Papanicolaou?

Mujer: Si, cuando estuve embarazada del chiquito y gracias a dios han salido bien, todo

Sara: Ha recibido cuando no ha estado embarazada

Mujer: Ah Sí, también

Sara: Y donde ha recibido?

Mujer: En el centro de salud Benjamín creo que se llama el centro de salud de aquí cerca

Sara: Y quien realizo la prueba?

Mujer: La enfermera, se llama Martita el apellido no lo sé pero así sé que llama

Title: Mantica-6.docx

Descriptor Info: Age: Interview Location: Residence: Interviewer: Date of Interview:

Codes Applied: Experiences with Pap

Excerpt Package: 1748

Excerpt End: 2361

ALEXANDRA: Y usted ha oído de la prueba de pap antes de hoy]?

MUJER: Sí, actualmente las mujeres debemos estar pendientes con nuestro pap mensualmente

ALEXANDRA: Entonces usted se ha realizado pruebas de pap?

MUJER: Sí, me hago mi pap

ALEXANDRA: Y son las doctoras las que se lo realizan, un doctor o las enfermeras?

MUJER: Es una enfermera la que lo hace, luego la enfermera de base te lee el resultado y si hay algún problema te dan un tratamiento

ALEXANDRA: Se tardan muchos días en darte los resultados?

MUJER: No, dependiendo, siempre me han dicho aquí que eso lo mandan al HEODRA y siempre dilata una semana

Title: Los Leches-3.docx

Descriptor Info: Age: Interview Location: Residence: Interviewer: Date of Interview:

Codes Applied: Experiences with Pap

Excerpt Package: 1879

Excerpt End: 2168

SARA: Ha tenido una prueba de Papanicolaou alguna vez, en la matriz, es un examen pélvico?

MUJER: Si

SARA: Y cuál fue el contexto estaba embarazada?

MUJER: Si, lo estaba

SARA: Cuando recibió la prueba de pap?

MUJER: A mí lo único que me sale en la prueba es que tengo inflamado los ovarios

Title: Santa Ana-1.docx

Descriptor Info: Age: Interview Location: Residence: Interviewer: Date of Interview:

Codes Applied: Experiences with Pap

Excerpt Package: 2076

Excerpt End: 2477

SARA: Ha tenido una prueba de Papanicolaou alguna vez?

MUJER: Que es eso?

SARA: Un Papanicolaou, un examen en su matriz

MUJER: Ah, sí, si lo he tenido

SARA: Y cuál ha sido el contexto de esta prueba, fue durante un embarazo o es regular de hacérselas?

MUJER: Sí, es regular me las hagan aquí

SARA: Las ha recibido aquí en el centro

MUJER: Si, si

SARA: Y quien la realizo la prueba

MUJER: Una doctora

Title: Los Leches-2.docx

Descriptor Info: Age: Interview Location: Residence: Interviewer: Date of Interview:

Codes Applied: Experiences with Pap

Excerpt Package: 1252

Excerpt End: 1448

HANNAH: Ha recibido una prueba de Papanicolaou alguna vez?

MUJER: Si

HANNAH: Y como ha sido el contexto de la prueba?

MUJER: De inflamación de humedad todo eso sale ya me los he hecho varias veces

Title: Perla-4.docx

Descriptor Info: Age: Interview Location: Residence: Interviewer: Date of Interview:

Codes Applied: Experiences with Pap

Excerpt Package: 1282

Excerpt End: 1369

HANNAH: Y con qué frecuencia usted se realiza una prueba de pap?

MUJER: Cada seis meses

Title: Perla-5.docx

Descriptor Info: Age: Interview Location: Residence: Interviewer: Date of Interview:

Codes Applied: Experiences with Pap

Excerpt Package: 6852

Excerpt End: 7089

ALEXA: Y se ha realizado una prueba de pap alguna vez?

MUJER: Si, ya.

ALEXA: Y como fue el contexto?

MUJER: Que la primera vez que me hicieron la prueba de pap? Pues estaba nerviosa yo creía que era algo diferente y pues fue algo rápido.

Title: Santa Ana-3.docx

Descriptor Info: Age: Interview Location: Residence: Interviewer: Date of Interview:

Codes Applied: Experiences with Pap

Excerpt Package: 106

Excerpt End: 699

HANNAH: Perfecto y dígame un poco sobre su experiencia con el cuidado de salud?

MUJER: Bueno mi experiencia con la salud, es que ahorita estamos combatiendo el cáncer de mama, la campaña es revísate, tócate para que no te pase, que hay que hacerse el autoexamen de mamas, que hay que estarse revisando que si no tienen pelotas, sino se les brotan las manos también, entonces ese es uno de los exámenes que yo me hago cuidadosamente, me reviso me hago mi pap a tiempo cada seis meses y si siento que tengo una media picazón me voy inmediatamente al centro de salud para que me den tratamiento.

Title: Perla-5.docx

Descriptor Info: Age: Interview Location: Residence: Interviewer: Date of Interview:

Codes Applied: Experiences with Pap

Excerpt Package: 7312

Excerpt End: 7488

ALEXA: Y cuáles son sus preocupaciones acerca de la prueba de pap?

MUJER: Bueno, yo ya me la realice y todo salió bien Gracias a Dios, entonces no hay preocupaciones sobre eso?

**Fear**

Title: Los Leches-1 (1).docx

Descriptor Info: Age: Interview Location: Residence: Interviewer: Date of Interview:

Codes Applied: Fear

Excerpt Package: 3613

Excerpt End: 3861

Sara: Were you afraid of this?

Woman: Yes, and no longer wanted to eat, heartbroken and I felt like dead but when I received the news from the doctor that I was healthy I felt relive, trough the review of pap I returned to life because I felt dead.

Title: Santa Ana-1.docx

Descriptor Info: Age: Interview Location: Residence: Interviewer: Date of Interview:

Codes Applied: Fear

Excerpt Package: 3389

Excerpt End: 3537

SARA: Cuáles son sus preocupaciones acerca de la salud sexual? Tiene alguna?

MUJER: La verdad que no, porque si estoy con mi pareja no tengo por que

Title: Perla-5.docx

Descriptor Info: Age: Interview Location: Residence: Interviewer: Date of Interview:

Codes Applied: Fear

Excerpt Package: 7810

Excerpt End: 8647

ALEXA: Ah que bien y cuando escucha la palabra cáncer de cuello uterino o cáncer cervical en que piensa? Oh las ideas que tiene con el cáncer de cuello uterino?

MUJER: Bueno, la información que tengo sobre cáncer uterino pues la verdad que en conocimiento científico especifico es poco pero así a nivel empírico puedo decir que lo primero que se me viene a la mente es que es alguna enfermedad peligrosa algo que es delicado para toda mujer, algo preocupante cuando eso viene a mi mente pienso en muerte, que tal vez tengo que hacerme un tratamiento fuerte para eso, no recuerdo como se llama eso que le hacen a las personas con cáncer.

ALEXA: Como quimioterapias?

MUJER: Si, eso o que lo operen, que le saquen la matriz y no vuelvan a tener hijos y pues eso es lo que pienso yo y mi conocimiento es que es una enfermedad bien peligrosa

Title: Mantica-2.docx

Descriptor Info: Age: Interview Location: Residence: Interviewer: Date of Interview:

Codes Applied: Fear

Excerpt Package: 4570

Excerpt End: 5889

ALEXANDRA: Usted conoce a alguien un familiar amigo o conocido que ha sufrido del vph o el cáncer de cuello uterino

MUJER: Familiar no, solo a una amiga que le dijeron que tenía esa enfermedad pero al final no, solo fue una equivocación

ALEXANDRA: Pero ella tenía mucho miedo cuando los doctores le dijeron

MUJER: Si, muchísimo miedo, ese caso yo lo vi en Estelí y ella tenía mucho miedo porque ella miro una muchacha salió llorando de donde la estaban revisando y a ella le dio mucho miedo porque no se lo que le hacen pero ella me conto que le dio mucho miedo

ALEXANDRA: Ella estaba llorando

MUJER: Si ella lloraba porque no sé qué le habían hecho pero le había dolido mucho entonces ella tenía mucho miedo pero Gracias a dios salió muy bien

ALEXANDRA: Cuanto le afectara su vida de su amiga a otras mujeres cuando ellas se dan cuenta que tienen vph

MUJER: Yo digo que eso afecta mucho, porque te deprime y no es fácil tener una enfermedad así

ALEXANDRA: Y sabe usted como es el tratamiento o el proceso después

MUJER: Realmente no se

ALEXANDRA: Cuando escuchas por el ejemplo el papiloma o el cáncer de cuello uterino que piensas de la palabra cáncer

MUJER: Me da mucho miedo solo de pensarlo, porque verdaderamente no sé si es algo que tiene cura o no y es algo que me da mucho miedo solo de escuchar la palabra

Title: Mantica-4.docx

Descriptor Info: Age: Interview Location: Residence: Interviewer: Date of Interview:

Codes Applied: Fear

Excerpt Package: 4485

Excerpt End: 4792

HANNAH: Y ahora tiene otras preocupaciones para su futuro o piensa que sabe lo suficiente para vivir una vida saludable sin cáncer?

MUJER: Pues me lleno de preocupaciones porque tengo mis hijos y no sé qué vaya a pasar con el tiempo pero siempre estoy agarrada de las manos de Dios que no me va a desamparar

Title: Mantica-1.docx

Descriptor Info: Age: Interview Location: Residence: Interviewer: Date of Interview:

Codes Applied: Fear

Excerpt Package: 2595

Excerpt End: 2841

Sara: Cuáles son sus preocupaciones con la prueba de Pap?

Mujer: Pues que me vaya a salir con una enfermedad, con el cáncer, porque ahora las mujeres sino se hacen eso y no se revisan a tiempo se pueden dar cuenta que tienen cáncer y pueden morir

Title: Los Leches-2.docx

Descriptor Info: Age: Interview Location: Residence: Interviewer: Date of Interview:

Codes Applied: Fear

Excerpt Package: 5412

Excerpt End: 5606

HANNAH: Otra pregunta acerca del cáncer, que piensa cuando escucha la palabra cáncer?

MUJER: Cuando uno escucha a alguien que tiene esta enfermedad uno piensa que le puede dar y da mucho miedo

Title: Perla-2.docx

Descriptor Info: Age: Interview Location: Residence: Interviewer: Date of Interview:

Codes Applied: Fear

Excerpt Package: 3101

Excerpt End: 3231

ALEXANDRA: Y si usted contrae el cáncer cervical como afectara esto a su vida?

MUJER: Pues desde ahorita nos tiene preocupados eso

Title: Mantica-4.docx

Descriptor Info: Age: Interview Location: Residence: Interviewer: Date of Interview:

Codes Applied: Fear

Excerpt Package: 3921

Excerpt End: 4313

HANNAH: Y piensa que antes de su diagnostico ha recibido suficiente información para hacer decisiones sobre su salud sexual?

MUJER: Si, por lo menos por mi situación me dijeron que no podía tener relaciones sin el preservativo, sin usar el método y aun así siempre aunque estuviera con el preservativo el problema no se iba a detener, entonces eso es lo que estoy viviendo una vida muy pésima

Title: Perla-4.docx

Descriptor Info: Age: Interview Location: Residence: Interviewer: Date of Interview:

Codes Applied: Fear

Excerpt Package: 7252

Excerpt End: 7641

HANNAH: Y tiene algunas preocupaciones acerca de la salud sexual?

MUJER: Bueno, claro que si porque mire, mi pareja no vive aquí, vive fuera del país y usted sabe que uno no sabe si pueden traer una enfermedad porque allá pueden vivir con alguien más y después se lo vienen a transmitir a uno

HANNAH: Y su pareja puede regresar con esa enfermedad? Y por eso tiene miedo?

MUJER: Si, así es

Title: Perla-3.docx

Descriptor Info: Age: Interview Location: Residence: Interviewer: Date of Interview:

Codes Applied: Fear

Excerpt Package: 12800

Excerpt End: 13378

HANNAH: Que tan preocupada está usted acerca del cáncer de cuello uterino?

MUJER: Pues SI preocupa porque es tu vida y además que no es solo tu vida sino que también perjudicas al núcleo familiar a las personas que tienes en tu hogar porque no solo tú te sientes preocupada sino que también se preocupa la gente que vive contigo en tu hogar, porque piensa, hay cáncer te vas a morir y así inicia uno a pensar con el cerebro mucho y se pone uno estresado al igual la familia por ese problema ósea no hay una vida en ese momento en el que una persona se da cuenta de ese problema.

Title: Mantica-3.docx

Descriptor Info: Age: Interview Location: Residence: Interviewer: Date of Interview:

Codes Applied: Fear

Excerpt Package: 1002

Excerpt End: 1562

HANNAH: Y como su madre encontró su cáncer?

MUJER: Ella fue a pasar consulta a la clínica donde estaba asegurada, se lo detectaron estuvo con tratamiento le dijeron que debía sacarse la matriz ella no quiso y producto de eso fue que falleció, ella tomo todos los medicamentos pero como no quiso sacarse la matriz entonces ahí fue donde falleció

HANNAH: Porque el tratamiento fue muy tarde cree?

MUJER: No, fue porque la última indicación del médico era que se sacara la matriz y ella se negó entonces ahí ya no pudo hacerse más nada solamente esperar su muerte

Title: Perla-3.docx

Descriptor Info: Age: Interview Location: Residence: Interviewer: Date of Interview:

Codes Applied: Fear

Excerpt Package: 2519

Excerpt End: 2779

HANNAH: Piensa que las chicas adolescentes y las mujeres hoy día tienen suficiente información acerca de la salud sexual?

MUJER: Quizás muchas, porque tienen temor de tener alguna enfermedad, de salir embarazadas pero otras no y por curiosidad les gusta andar.

Title: SantaAna-4.docx.docx

Descriptor Info: Age: Interview Location: Residence: Interviewer: Date of Interview:

Codes Applied: Fear

Excerpt Package: 6383

Excerpt End: 7077

ALEXANDRA: Cuales precauciones toma usted para prevenir esas enfermedades y para mantenerse segura con su salud?

MUJER: Bueno yo ahorita de eso no me he hecho nada para la enfermedad pero si me lo voy a hacer porque a como miro tantas mujeres padeciendo eso y es mejor verlo antes porque si pasa tiempo con esa enfermedad ya no tiene cura, porque si uno tiene esas cosas así uno le dan el tratamiento para curarse, pero debe ser con disposición porque si digo que hasta más adelante entonces eso no funciona sí, porque tengo que pensar en mis hijos y no dejarlos porque todavía están pequeños porque me daría tristeza que ellos estén llorándome ahí en una cama y yo primero pienso en mis hijos.

Title: Perla-3.docx

Descriptor Info: Age: Interview Location: Residence: Interviewer: Date of Interview:

Codes Applied: Fear

Excerpt Package: 12800

Excerpt End: 13378

HANNAH: Que tan preocupada está usted acerca del cáncer de cuello uterino?

MUJER: Pues SI preocupa porque es tu vida y además que no es solo tu vida sino que también perjudicas al núcleo familiar a las personas que tienes en tu hogar porque no solo tú te sientes preocupada sino que también se preocupa la gente que vive contigo en tu hogar, porque piensa, hay cáncer te vas a morir y así inicia uno a pensar con el cerebro mucho y se pone uno estresado al igual la familia por ese problema ósea no hay una vida en ese momento en el que una persona se da cuenta de ese problema.

Title: Mantica-1.docx

Descriptor Info: Age: Interview Location: Residence: Interviewer: Date of Interview:

Codes Applied: Fear

Excerpt Package: 7535

Excerpt End: 7720

Sara: Y va a hablar con sus hijos cuando sean grandes acerca de estas enfermedades, porque?

Mujer: Si porque uno sufre, cae en cama y lo van matando poco a poco, todas esas enfermedades

Title: Perla-2.docx

Descriptor Info: Age: Interview Location: Residence: Interviewer: Date of Interview:

Codes Applied: Fear

Excerpt Package: 6770

Excerpt End: 6997

ALEXANDRA: Y tiene algunas preocupaciones del pap o cree que es seguro el examen?

MUJER: A veces quisiera que los resultados se confundieran cuando salen cosas malas, yo deseara se hubiesen confundido o que no hayan visto bien.

Title: Perla-5.docx

Descriptor Info: Age: Interview Location: Residence: Interviewer: Date of Interview:

Codes Applied: Fear

Excerpt Package: 13269

Excerpt End: 13688

ALEXA: Cuales son sus preocupaciones más grandes acerca de su salud y salud sexual?

MUJER: Bueno, siempre hay que tener cuidado, preocupaciones siempre van a haber porque no sabemos a lo largo del tiempo y mañana no se sabe, entonces considero que siempre debo estarme chequeando porque uno nunca sabe que un quiste y puede ser un cáncer, entonces uno nunca sabe y por eso siempre debe estar pendiente con la situación.

Title: Los Leches-3.docx

Descriptor Info: Age: Interview Location: Residence: Interviewer: Date of Interview:

Codes Applied: Fear

Excerpt Package: 4603

Excerpt End: 4869

SARA: Como es el conocimiento y actitud de la población acerca del cáncer de cuello uterino?

MUJER: Bueno la verdad es que eso no se lo deseo a nadie porque es una enfermedad que lo va comiendo por dentro a uno pero si eso es lo que Dios nos manda hay que aceptarlo

Title: Mantica-6.docx

Descriptor Info: Age: Interview Location: Residence: Interviewer: Date of Interview:

Codes Applied: Fear

Excerpt Package: 5352

Excerpt End: 5883

ALEXANDRA: Y ha oído del virus de papiloma humano antes de hoy?

MUJER: cercano a mí a mis alrededores con mis familiares no, pero si por medio del internet y personas que se han relacionado con personas que se ven demasiado tarde lo he escuchado y lo he seguido demasiado cerca para saber más sobre los resultados y que te da, es donde te da temor y por eso yo asiste con más frecuencia a los centros de salud por eso es que hay que sacarlo del tabú y hablarlo más para que uno no se alarme para que se sepa de que hay que cuidarse

Title: Mantica-5.docx

Descriptor Info: Age: Interview Location: Residence: Interviewer: Date of Interview:

Codes Applied: Fear

Excerpt Package: 4792

Excerpt End: 4941

HANNAH: Bueno, y que piensa usted cuando escucha la palabra cáncer?

MUJER: Pues la verdad no me gusta oírlo porque me parece que eso anda en el aire.

Title: Los Leches-2.docx

Descriptor Info: Age: Interview Location: Residence: Interviewer: Date of Interview:

Codes Applied: Fear

Excerpt Package: 4290

Excerpt End: 4741

HANNAH: Tiene algunas preocupaciones acerca de su salud sexual?

MUJER: No

HANNAH: Como percibe el riesgo del virus de papiloma humano?

MUJER: Como se percibe?

HANNAH: Si piensa que es un virus muy común que puede afectarle en el futuro?

MUJER: Si porque si mi marido anda con muchas mujeres podría ser que me pase

HANNAH: Y con el cáncer de cuello uterino piensa que puede afectarle en el futuro?

MUJER: Si usted sabe que eso sale en cualquier momento

Title: Perla-3.docx

Descriptor Info: Age: Interview Location: Residence: Interviewer: Date of Interview:

Codes Applied: Fear Cervical cancer

Excerpt Package: 10443

Excerpt End: 10669

HANNAH: Y cómo afecta la enfermedad en su vida?

MUJER: Pues ella se sintió emocionalmente triste porque ella pensó que su vida sexual ya no iba a continuar pero le dieron terapias psicológicas y le dijeron que todo era normal.

Title: Perla-3.docx

Descriptor Info: Age: Interview Location: Residence: Interviewer: Date of Interview:

Codes Applied: Fear Cervical cancer

Excerpt Package: 10443

Excerpt End: 10669

HANNAH: Y cómo afecta la enfermedad en su vida?

MUJER: Pues ella se sintió emocionalmente triste porque ella pensó que su vida sexual ya no iba a continuar pero le dieron terapias psicológicas y le dijeron que todo era normal.

Title: Perla-3.docx

Descriptor Info: Age: Interview Location: Residence: Interviewer: Date of Interview:

Codes Applied: Fear Cervical cancer

Excerpt Package: 10670

Excerpt End: 11038

HANNAH: Y ella recibió quimioterapia?

MUJER: No

HANNAH: Y radioterapia?

MUJER: No, nada de eso pero si le dieron terapia psicológica, me entiende, para así decirle a ella que no se preocupara y que podía tener su vida normal con cualquier otra pareja que ella decidiera y que no había ningún problema aunque le hayan sacado la matriz su vida sexual siempre es normal.

Title: Perla-3.docx

Descriptor Info: Age: Interview Location: Residence: Interviewer: Date of Interview:

Codes Applied: Fear Cervical cancer Support

Excerpt Package: 10670

Excerpt End: 11038

HANNAH: Y ella recibió quimioterapia?

MUJER: No

HANNAH: Y radioterapia?

MUJER: No, nada de eso pero si le dieron terapia psicológica, me entiende, para así decirle a ella que no se preocupara y que podía tener su vida normal con cualquier otra pareja que ella decidiera y que no había ningún problema aunque le hayan sacado la matriz su vida sexual siempre es normal.

Title: Los Leches-1 (1).docx

Descriptor Info: Age: Interview Location: Residence: Interviewer: Date of Interview:

Codes Applied: Fear HPV

Excerpt Package: 4424

Excerpt End: 4784

Sara: Do you know of someone who has the virus?

Woman: Yes, I’ve heard from people in my hamlet. (caserio)

Sara: And, how was the experience of them?

Woman: I’ve heard only that were in treatment that is expensive and thank God has not gave me, because I do not know what would have done, because is a virus I think, but thank God I have not had that problem.

Title: Los Leches-1 (1).docx

Descriptor Info: Age: Interview Location: Residence: Interviewer: Date of Interview:

Codes Applied: Fear Intimacy

Excerpt Package: 6654

Excerpt End: 6917

Sara: Do you feel more comfortable with doctors and nurses who knows?

Woman: No, I feel more comfortable with persons that only came once and doesn’t comes back, that are not permanent I feel better like that because I am ashamed with the ones that are from here.

Title: Los Leches-1 (1).docx

Descriptor Info: Age: Interview Location: Residence: Interviewer: Date of Interview:

Codes Applied: Fear Intimacy Confidentiality

Excerpt Package: 6115

Excerpt End: 6653

Sara: I get it, and what do you think when heard the word cancer?

Woman: Well, I think that sometimes that happens because we don’t go to the health post and we don’t want that the doctors or nurses check us because we are ashamed, at first I did not want to come here because here everybody knows me and in other place don’t, so that was the main reason that I went to Mantica at the beginning, but now what I do is that I only take consult with persons that are not permanent in the center and to that person I say that make me the pap.

Title: Perla-4.docx

Descriptor Info: Age: Interview Location: Residence: Interviewer: Date of Interview:

Codes Applied: Fear Pap smear

Excerpt Package: 6032

Excerpt End: 6445

HANNAH: Como le afecta a su vida?

MUJER: La verdad que a veces yo no quiero porque me van a decir algo y tal vez me van a acelerar el poco tiempo que tengo de vida cuando uno se da cuenta de una enfermedad como que se aflige mas

HANNAH: Ahora usted viene cada seis meses?

MUJER: Si tengo que estar viniendo porque el doctor dice que aún no me puede dar de baja que debo de seguir en chequeos así que aquí estamos.

Title: Santa Ana-1.docx

Descriptor Info: Age: Interview Location: Residence: Interviewer: Date of Interview:

Codes Applied: Fear Promiscuity

Excerpt Package: 5919

Excerpt End: 6021

SARA: Y que tan preocupada está usted ahora

MUJER: Pues no lo estoy porque tengo a mi pareja mi marido

Title: Santa Ana-2.docx

Descriptor Info: Age: Interview Location: Residence: Interviewer: Date of Interview:

Codes Applied: Fear Support

Excerpt Package: 4497

Excerpt End: 4868

ALEXANDRA: Y cuáles son sus preocupaciones con respecto a la prueba de pap?

MUJER: Bueno por lo menos que tenga cáncer o alguna enfermedad venérea porque usted sabe si le sale un cáncer ya uno mejor dicho está listo porque ahí es solo de esperar porque ya vienen las quimioterapias y que se yo que más cosas y ya solo esperar hasta que se lo levante el señor o un milagro

**HPV**

Title: Perla-5.docx

Descriptor Info: Age: Interview Location: Residence: Interviewer: Date of Interview:

Codes Applied: HPV

Excerpt Package: 12850

Excerpt End: 13268

ALEXA: Y hay una relación entre el papiloma y el cáncer de cuello uterino?

MUJER: Pues yo creo que si hay una relación pero así algo científico no le podría decir, pero siento de que si porque es algo íntimo de nosotras y tanto si nuestra pareja o nosotras no sabemos lo que tenemos nos estamos haciendo daño y aumentando enfermedades si andamos en malos pasos y malos caminos más si tenemos relaciones sin protección.

Title: Santa Ana-3.docx

Descriptor Info: Age: Interview Location: Residence: Interviewer: Date of Interview:

Codes Applied: HPV

Excerpt Package: 700

Excerpt End: 1625

HANNAH: Si, bueno, gracias, dígame un poco acerca de su experiencia con la educación sexual?

MUJER: Bueno la educación de salud sexual está en que por ejemplo que uno se debe de proteger porque hay muchas enfermedades como el virus de papiloma humano y el VIH por eso hay que protegerse y cuidarse porque además no solamente las pueden transmitir por el sexo, también se puede transmitir si una persona que está infectada con VIH se corta y hacen contacto de sangre con sangre se pueden transmitir o una aguja.

HANNAH: Y de dónde aprendió sobre estos temas?

MUJER: Bueno, en los colegios aprendemos, las charlas que va haciendo el centro de salud que las dan constantemente sobre las enfermedades virales como las podemos evitar y que debemos hacer para tener un mejor cuidado

HANNAH: Y la información sobre le vph y el cáncer de cuello uterino lo ha tenido del colegio?

MUJER: No, del centro de salud de las charlas que dan

Title: Mantica-4.docx

Descriptor Info: Age: Interview Location: Residence: Interviewer: Date of Interview:

Codes Applied: HPV

Excerpt Package: 5072

Excerpt End: 5699

HANNAH: Y usted piensa que hay una relación entre el virus de papiloma humano y el cáncer de cuello uterino, ha recibido información sobre el virus de papiloma humano antes de su diagnostico?

MUJER: Por ejemplo, que si es transmitido por relaciones sexuales, bueno lo que yo sé hasta el momento es que el virus es trasmitido por una relación sexual

HANNAH: Y piensa que hay una relación entre el virus y el cáncer?

MUJER: Como así?

HANNAH: El virus de papiloma humano puede causar cáncer de cuello uterino?

MUJER: Si

HANNAH: Hay una relación muy fuerte?

MUJER: Sí, yo digo que sí, esa es la información que me ha dado mi medico

Title: Poneloya-1.docx

Descriptor Info: Age: Interview Location: Residence: Interviewer: Date of Interview:

Codes Applied: HPV

Excerpt Package: 2232

Excerpt End: 2530

ALEXANDRA: Si, está perfecto, ha escuchado del virus de papiloma humano antes de hoy?

MUJER: Si, pero no me acuerdo los síntomas, nos informan bastante al respecto del VPH, el herpes, el SIDA y el cáncer y otras enfermedades de transmisión sexual que además se transmiten por jeringa y otras cosas.

Title: Mantica-2.docx

Descriptor Info: Age: Interview Location: Residence: Interviewer: Date of Interview:

Codes Applied: HPV

Excerpt Package: 5316

Excerpt End: 5609

ALEXANDRA: Cuanto le afectara su vida de su amiga a otras mujeres cuando ellas se dan cuenta que tienen vph

MUJER: Yo digo que eso afecta mucho, porque te deprime y no es fácil tener una enfermedad así

ALEXANDRA: Y sabe usted como es el tratamiento o el proceso después

MUJER: Realmente no se

Title: Mantica-1.docx

Descriptor Info: Age: Interview Location: Residence: Interviewer: Date of Interview:

Codes Applied: HPV

Excerpt Package: 5208

Excerpt End: 6360

Sara: En que enfermedades esta pensando, en cáncer, cáncer de cuello uterino y también hay otra enfermedad como el virus de papiloma humano, ha escuchado hablar de estas como percibe el riesgo del virus papiloma humano

Mujer: Bueno yo he visto en la televisión, que el hombre se lo pasa a la mujer pero no se si al hombre no le avanza o qué, pero no sé si a la mujer le salen unas como arrugas, casi solo en la televisión y en los centros de salud he escuchado pero como se me olvida todo, no recuerdo mucho de eso, pero que ese virus si he escuchado que el hombre se lo transmite a la mujer

Sara: Si un hombre a la mujer. Quiere más información sobre el vph?

Mujer: Que si hay más enfermedades?

Sara: Que si quiere más información sobre esta enfermedad?

Mujer: Si por que casi no se, se me ha olvidado pero me han dicho bastante

Sara: Como es el conocimiento y actitud de la comunidad en general sobre el virus de papiloma humano, es algo que puede hablar con amigos familia es muy común de hablar?

Mujer: Si cuando voy al centro de salud dan charlas, pero como le digo se me olvida pero si dan bastantes charlas de todas esas enfermedades malignas

Title: Subtiava-2.docx

Descriptor Info: Age: Interview Location: Residence: Interviewer: Date of Interview:

Codes Applied: HPV

Excerpt Package: 3297

Excerpt End: 3872

HANNAH: Y ha oído del virus de papiloma humano alguna vez?

MUJER: Bueno si, pero lo único que entiendo es que dicen que se transmite por medio de relaciones sexuales

HANNAH: Y puede detectarse por medio de la prueba de Pap?

MUJER: Si, si se puede detectar.

HANNAH: Bueno, donde aprendió acerca de ese virus?

MUJER: Aquí, en el centro siempre están capacitando y te están diciendo, y ponen también murales informativos y así uno puede educarse por medio de la vista, al menos yo si los leo y así uno está leyendo además las doctoras y enfermeras te dicen y así te das cuenta

Title: Mantica-2.docx

Descriptor Info: Age: Interview Location: Residence: Interviewer: Date of Interview:

Codes Applied: HPV

Excerpt Package: 4288

Excerpt End: 4569

ALEXANDRA: Ha oído hablar del papiloma

MUJER: Sí, más o menos

ALEXANDRA: Y usted piensa que hay un riesgo de esto para las mujeres

MUJER: Yo digo que sí, porque vi en internet que esto se pasa por las relaciones sexuales entonces digo yo que si hay un riesgo para todas las mujeres

Title: Perla-3.docx

Descriptor Info: Age: Interview Location: Residence: Interviewer: Date of Interview:

Codes Applied: HPV

Excerpt Package: 5648

Excerpt End: 6818

HANNAH: Piensa que es un virus muy común?

MUJER: Común porque en la mayoría de las mujeres existe eso, pero es porque no se cuidan, independientemente que tengamos a la pareja, la pareja también tiene que cuidarse y tener conciencia de que si el agarra un virus o bacteria las afectadas somos nosotras, yo por lo menos yo le digo a mi pareja el día que yo tenga una enfermedad usted me va a pagar todos mis costos aunque sea incurable pero le va a tener que costar para que aprenda a respetar y tenga conciencia.

HANNAH: Y cómo piensa que es la actitud y el conocimiento de la comunidad acerca de este virus?

MUJER: Mucha gente está informada sobre eso, porque en los centros de salud hay rótulos y hay que leerlos mientras toca la cita en la consulta pero muchas personas no le prestan importancia pero es muy importante porque ya ha habido muchas muertes sobre eso y hay muertes de mujeres que tienen la enfermedad del papiloma humano por lo mismo

HANNAH: Muerte por el virus o de otra cosa?

MUJER: No, de eso por el virus porque yo por ejemplo yo tengo una tía y mi tía tiene eso y ella no se recuperaba, esta delgada porque eso te enferma psicológicamente también.

Title: SantaAna-4.docx.docx

Descriptor Info: Age: Interview Location: Residence: Interviewer: Date of Interview:

Codes Applied: HPV

Excerpt Package: 4591

Excerpt End: 4747

ALEXANDRA: Y ha oído del virus de papiloma humano?

MUJER: No, eso no lo he oído

ALEXANDRA: Pero y del cáncer de cuello uterino?

MUJER: Si, ese si lo he oído

Title: Mantica-4.docx

Descriptor Info: Age: Interview Location: Residence: Interviewer: Date of Interview:

Codes Applied: HPV

Excerpt Package: 5072

Excerpt End: 5699

HANNAH: Y usted piensa que hay una relación entre el virus de papiloma humano y el cáncer de cuello uterino, ha recibido información sobre el virus de papiloma humano antes de su diagnostico?

MUJER: Por ejemplo, que si es transmitido por relaciones sexuales, bueno lo que yo sé hasta el momento es que el virus es trasmitido por una relación sexual

HANNAH: Y piensa que hay una relación entre el virus y el cáncer?

MUJER: Como así?

HANNAH: El virus de papiloma humano puede causar cáncer de cuello uterino?

MUJER: Si

HANNAH: Hay una relación muy fuerte?

MUJER: Sí, yo digo que sí, esa es la información que me ha dado mi medico

Title: Perla-2.docx

Descriptor Info: Age: Interview Location: Residence: Interviewer: Date of Interview:

Codes Applied: HPV

Excerpt Package: 4430

Excerpt End: 4965

ALEXANDRA: Y ha oído del virus de papiloma humano?

MUJER: Dicen que es eso, es decir que es el mismo cáncer.

ALEXANDRA: Y como es la relación entre el papiloma y el cáncer?

MUJER: No sé, dicen que eso se contagia

ALEXANDRA: Y si tuviera un diagnostico positivo del papiloma humano quiere decir que tiene cáncer de cuello uterino?

MUJER: Aja, yo digo que sí.

ALEXANDRA: Cree que hay un método eficaz que haga reducir significativamente el riesgo de esta enfermedad?

MUJER: Estoy pidiéndole a Dios que sí que haya algo que la pueda curar

Title: Santa Ana-2.docx

Descriptor Info: Age: Interview Location: Residence: Interviewer: Date of Interview:

Codes Applied: HPV

Excerpt Package: 5268

Excerpt End: 5497

ALEXANDRA: Y ha oído del virus de papiloma humano?

MUJER: Yo he oído hablar de eso fíjese y tuvimos una charla también.

ALEXANDRA: Es muy común aquí?

MUJER: Si porque fíjese que casi no se sabe esa enfermedad aquí, Gracias a Dios

Title: Los Leches-1 (1).docx

Descriptor Info: Age: Interview Location: Residence: Interviewer: Date of Interview:

Codes Applied: HPV

Excerpt Package: 4137

Excerpt End: 4423

Sara: Yes, you are healthy in your sexual health, have you heard of human papillomavirus? How do you perceived risk of this?

Woman: At least I do not know about that, like has not given me I hear people say that’s ugly, feels like a horrible burn, but I do not know because I never had.

Title: Perla-4.docx

Descriptor Info: Age: Interview Location: Residence: Interviewer: Date of Interview:

Codes Applied: HPV

Excerpt Package: 535

Excerpt End: 1055

HANNAH: Y usted me dijo que tiene del virus de papiloma humano?

MUJER: Si

HANNAH: Cuando supo y porque usted tenía ese virus?

MUJER: Hace doce años supe

HANNAH: Y lo supo de una prueba de pap?

MUJER: Si con una colcospia

HANNAH: Y que sabe acerca de este virus?

MUJER: Bueno que hay que prevenir y que hay que usar condón en las relaciones pues hasta el momento lo mío está controlado pero yo siempre sigo en mi control con el ginecólogo porque dice que ese virus adormece y puede volver a aparecer en cualquier momento

Title: Mantica-6.docx

Descriptor Info: Age: Interview Location: Residence: Interviewer: Date of Interview:

Codes Applied: HPV

Excerpt Package: 5352

Excerpt End: 6435

ALEXANDRA: Y ha oído del virus de papiloma humano antes de hoy?

MUJER: cercano a mí a mis alrededores con mis familiares no, pero si por medio del internet y personas que se han relacionado con personas que se ven demasiado tarde lo he escuchado y lo he seguido demasiado cerca para saber más sobre los resultados y que te da, es donde te da temor y por eso yo asiste con más frecuencia a los centros de salud por eso es que hay que sacarlo del tabú y hablarlo más para que uno no se alarme para que se sepa de que hay que cuidarse

ALEXANDRA: Y el papiloma es muy común entre las mujeres nicaragüenses?

MUJER: Es un virus silencioso por eso es que hay estarse chequeando porque a veces hay personas que lo tienen y no lo saben por eso es que hay que acercarse más a los centros de salud para que uno lo sepa por qué nosotros no lo sabemos

ALEXANDRA: Y cree que una parte de responsabilidad de esta enfermedad es en los hombres o es solo algo de las mujeres?

MUJER: es algo de los dos pero yo como mujer tengo que estar pendiente para verme y sentirme bien es parte más de uno propio

Title: Los Leches-3.docx

Descriptor Info: Age: Interview Location: Residence: Interviewer: Date of Interview:

Codes Applied: HPV

Excerpt Package: 3394

Excerpt End: 3707

SARA: Ha oído hablar de virus de papiloma humano?

MUJER: Si, ese es el más peligroso

SARA: Como percibe el riesgo de esto y sabe de alguien que tiene esta enfermedad?

MUJER: Si lo he escuchado en la televisión y aquí a veces que da charlas

SARA: Y en la televisión que dijeron?

MUJER: Que ese virus se da por sexo

Title: Mantica-5.docx

Descriptor Info: Age: Interview Location: Residence: Interviewer: Date of Interview:

Codes Applied: HPV

Excerpt Package: 4255

Excerpt End: 4791

HANNAH: Y sabe sobre el virus de papiloma humano?

MUJER: Si

HANNAH: Y que sabe sobre este virus?

MUJER: Bueno lo he visto que es una enfermedad igual al cáncer y tengo una hermana que sufrio de esa enfermedad, es algo doloroso el tratamiento porque la quemaban y cosas así

HANNAH: Piensa que hay una relación entre el virus de papiloma humano y el cáncer de cuello uterino o son cosas independientes?

MUJER: Son cosas independientes pero a la vez vienen siendo igual porque si el virus avanza se hace cáncer y ya no hay nada que hacer.

Title: Los Leches-1 (1).docx

Descriptor Info: Age: Interview Location: Residence: Interviewer: Date of Interview:

Codes Applied: HPV

Excerpt Package: 4785

Excerpt End: 5238

Sara: How is the knowledge and attitude of the community in general about HPV?

Woman: Is that SIDA

Sara: No, that’s other, HPV is papillomavirus.

Woman: Well. I heard about some persons who had SIDA in my community but I don’t know enough because like in my house we don’t have that problem, thank God in that we are very healthy with these, we just have regular disease pressure, hearth and nerves, we are really nervous, but not that kind of problems.

Title: Perla-3.docx

Descriptor Info: Age: Interview Location: Residence: Interviewer: Date of Interview:

Codes Applied: HPV

Excerpt Package: 7277

Excerpt End: 7602

HANNAH: Y piensa usted que hay una relación entre el virus de papiloma humano y el cáncer de cuello uterino? Ha oído del cáncer de cuello uterino?

MUJER: Yo pienso que es la misma cosa, es que me imagino que el papiloma humano tiene que ir relacionado con el cáncer uterino porque es lo mismo por eso pienso no hay diferencia

Title: Perla-5.docx

Descriptor Info: Age: Interview Location: Residence: Interviewer: Date of Interview:

Codes Applied: HPV

Excerpt Package: 11863

Excerpt End: 13268

ALEXA: El virus de papiloma humano es muy común aquí en Nicaragua?

MUJER: Si, he escuchado bastante por malas practicantes de relaciones sexuales en varias parejas.

ALEXA: Solo nos faltan unas preguntitas acerca del papiloma, como piensa que es la actitud de la comunidad acerca de esta enfermedad?

MUJER: Bueno eso le sucede más a los adolescentes bastante pero a como le digo es porque hay varones que andan con varias mujeres y talvez le transmiten esa enfermedad a su esposa o compañera y no le gusta que vaya a un centro de salud a tratar la enfermedad porque son personas machistas pero le estoy hablando de lugares talvez todavía del campo o comarcas y que todavía se dan esas situaciones porque es bastante tabú y aquí en la ciudad se da en los adolescentes que comienzan a tener relaciones sexuales y tal vez por pena no quieren ir a los centros de salud tal vez las muchachas y también los varones porque para ellos no es común es más común ver a las mujeres ir a ginecología.

ALEXA: Y hay una relación entre el papiloma y el cáncer de cuello uterino?

MUJER: Pues yo creo que si hay una relación pero así algo científico no le podría decir, pero siento de que si porque es algo íntimo de nosotras y tanto si nuestra pareja o nosotras no sabemos lo que tenemos nos estamos haciendo daño y aumentando enfermedades si andamos en malos pasos y malos caminos más si tenemos relaciones sin protección.

Title: Subtiava-1.docx

Descriptor Info: Age: Interview Location: Residence: Interviewer: Date of Interview:

Codes Applied: HPV

Excerpt Package: 5772

Excerpt End: 9036

HANNAH: Usted ha oído del virus de papiloma humano alguna vez? Puede hablar acerca de este virus un poco?

MUJER: Por lo menos ese que le pasaron a mi marido era uno de ellos porque en el centro me habían dado un folletito y ahí salía uno de eso y ahí salía uno de esos y yo lo lleve donde la doctora para saber cuál es el que él tiene y me dijo que el virus se llama cresta de gallo porque se le ponía como toda una flor arrugada y es horrible pero después se compuso.

Title: Perla-3.docx

Descriptor Info: Age: Interview Location: Residence: Interviewer: Date of Interview:

Codes Applied: HPV

Excerpt Package: 5648

Excerpt End: 6818

HANNAH: Piensa que es un virus muy común?

MUJER: Común porque en la mayoría de las mujeres existe eso, pero es porque no se cuidan, independientemente que tengamos a la pareja, la pareja también tiene que cuidarse y tener conciencia de que si el agarra un virus o bacteria las afectadas somos nosotras, yo por lo menos yo le digo a mi pareja el día que yo tenga una enfermedad usted me va a pagar todos mis costos aunque sea incurable pero le va a tener que costar para que aprenda a respetar y tenga conciencia.

HANNAH: Y cómo piensa que es la actitud y el conocimiento de la comunidad acerca de este virus?

MUJER: Mucha gente está informada sobre eso, porque en los centros de salud hay rótulos y hay que leerlos mientras toca la cita en la consulta pero muchas personas no le prestan importancia pero es muy importante porque ya ha habido muchas muertes sobre eso y hay muertes de mujeres que tienen la enfermedad del papiloma humano por lo mismo

HANNAH: Muerte por el virus o de otra cosa?

MUJER: No, de eso por el virus porque yo por ejemplo yo tengo una tía y mi tía tiene eso y ella no se recuperaba, esta delgada porque eso te enferma psicológicamente también.

Title: Poneloya-2.docx

Descriptor Info: Age: Interview Location: Residence: Interviewer: Date of Interview:

Codes Applied: HPV

Excerpt Package: 8292

Excerpt End: 9456

ALEXA: Y el virus papiloma es muy común en la comunidad?

MUJER: Si, eso es muy común.

ALEXA: Y como es la actitud de la comunidad acerca del papiloma?

MUJER: Algunos ni mente le ponen, no se la verdad será que debe de haber más cultura, hablarles más pero vuelvo y repito aquí les estás hablando y ellos están en otra cosa no les prestan atención a lo que uno les está diciendo y eso es algo importante más a los jóvenes, porque si eso lo tiene un joven y si yo tengo relaciones con ese que lo tiene, ese se lo transmite a otro, entonces a veces no todos nos gusta que nos den esas charlas, algunos lo toman como que es vulgaridad y ahora en las redes sociales que no ven ahí en ese internet, a veces publican cosas que no van al caso.

ALEXA: Y el papiloma tiene relación con el cáncer cervical el de cuello uterino?

MUJER: Digo yo que viene siendo igual, porque si no me cuido y eso va avanzando, se me va acumulando y no tomo nada de medicamento ni tengo tratamiento entonces eso se me va regando para dentro porque no se queda solo superficialmente sino que va para dentro y eso llega hasta tener llagas de eso y eso ya es más y si no te curas jamás se lograra.

Title: Santa Ana-1.docx

Descriptor Info: Age: Interview Location: Residence: Interviewer: Date of Interview:

Codes Applied: HPV Cervical cancer

Excerpt Package: 4743

Excerpt End: 5208

SARA: Si por eso es muy promovido, dijo que hay una diferencia, cual es la diferencia entre virus de papiloma y cáncer de cuello uterino, entre el conocimiento y la actitud de la comunidad

MUJER: Pues no podría decirlo, bueno que la gente habla que si es cáncer se trata de que si no se detecta a tiempo habría que limpiarle en todas las partes donde se da mientras que el papiloma se trata como de verrugas que se pueden aliviar con tratamiento y se pueden quitar.

Title: Mantica-4.docx

Descriptor Info: Age: Interview Location: Residence: Interviewer: Date of Interview:

Codes Applied: HPV Cervical cancer

Excerpt Package: 673

Excerpt End: 1390

HANNAH: Por medio de qué medida se dio cuenta por una prueba de Papanicolaou?

MUJER: Si Por el Papanicolaou me di cuenta que había un problema en la matriz

HANNAH: Y que dijo su médico acerca del cáncer en ese momento?

MUJER: Bueno me atienden en el hospital OSCAR DANILO ROSALES primero me hacían las quimioterapias pero como eso no funciono, tuvieron que hacerme un cono para cortar parte de la matriz y ver si así se detiene bueno actualmente no siento nada estoy siempre con mis consultas y Papanicolaou y viendo hasta donde esta y si se ha detenido y mi problema se llama papiloma humano pero supuestamente con el cono me que hicieron verían si se detenía entonces eso es lo que estamos esperando a ver qué pasa

Title: Santa Ana-2.docx

Descriptor Info: Age: Interview Location: Residence: Interviewer: Date of Interview:

Codes Applied: HPV Cervical cancer

Excerpt Package: 7331

Excerpt End: 7429

ALEXANDRA: Y cree que el cáncer tiene una relación con el virus de papiloma?

MUJER: Yo digo que si

Title: Mantica-3.docx

Descriptor Info: Age: Interview Location: Residence: Interviewer: Date of Interview:

Codes Applied: HPV Cervical cancer Promiscuity

Excerpt Package: 4374

Excerpt End: 4803

HANNAH: Que piensa es la relación entre el virus de papiloma humano y el cáncer de cuello uterino?

MUJER: Pues yo creo que tiene relación porque ese virus viene cuando las personas tienen muchas relaciones con diferentes personas y ese virus tengo entendido da cáncer y si no te lo ves a tiempo puede ser que fallezcas porque es un virus que no tiene virus o eso creo y que debes estar en tratamiento pero no te aseguran una cura

Title: Santa Ana-1.docx

Descriptor Info: Age: Interview Location: Residence: Interviewer: Date of Interview:

Codes Applied: HPV Private/taboo

Excerpt Package: 3538

Excerpt End: 3955

SARA: Ha oído hablar del virus de papiloma humano

MUJER: Si

SARA: Y como percibe el riesgo de esto?

MUJER: Pues la verdad es que aquí en Nicaragua se han dado muchos casos y hasta hay reportajes en la televisión todas esas cosas las anuncian para que las personas tengamos mucho más cuidado y usamos protección al tener relaciones

SARA: Sabe de alguien que tiene o ha tenido esta enfermedad

MUJER: Pues conocidos no

Title: Santa Ana-3.docx

Descriptor Info: Age: Interview Location: Residence: Interviewer: Date of Interview:

Codes Applied: HPV Sexual education

Excerpt Package: 6129

Excerpt End: 7570

HANNAH: Y puede hablar un poco acerca del virus de papiloma humano? Que sabe sobre este virus?

MUJER: Bueno el papiloma humano hasta hoy día se dice que se transmite por relaciones sexuales hoy en día dicen que se puede tratar igual que el cáncer pero el virus antes no tenía cura pero hoy en día se puede tratar y los síntomas del papiloma humano te salen llagas en la espalda, mucha fiebre, alucinaciones, te corta la vista

HANNAH: Y cual piensa es la actitud y el conocimiento del virus de papiloma humano en la comunidad?

MUJER: Bueno, el virus de papiloma humano es todo principalmente chequeo, ir al médico que las niñas vayan a la ginecóloga porque no es malo, aunque ellas aun no hayan tenido su primera experiencia sexual porque es bueno que ellas hablen y sepan más sobre la educadora sexual

HANNAH: Y como podría mejorar el conocimiento sobre el cáncer de cuello uterino y el vph en las mujeres adolescentes?

MUJER: Bueno yo digo mejorando, se tienen charlas en los centros educativos en los centros de salud y hay brigadas o asociaciones que vienen de fuera y les dan charlas a las jóvenes, las charlas siempre se están dando y educan a las profesores y son educado por los grupos de jóvenes por fuera que están asociados con personas con VIH-SIDA entonces el conocimiento esta, pero que no tienen prevención es otra cosa, porque el VIH no se pasa, porque me des la mano o por un beso en la mejía, entonces el conocimiento esta.

**Intimacy**

Title: Perla-3.docx

Descriptor Info: Age: Interview Location: Residence: Interviewer: Date of Interview:

Codes Applied: Intimacy

Excerpt Package: 4432

Excerpt End: 4941

HANNAH: Se siente cómoda recibiendo una prueba de pap de un médico masculino?

MUJER: Si claro, que incomoda porque no es lo normal.

HANNAH: Y piensa que tiene el poder para pedir que prefiere una enfermera o medica femenina para realizar la prueba o no hay eso?

MUJER: Pues si necesito que me hagan el examen tengo que aceptar quien me lo haga, lo que importa es que me hagan la prueba para ver si estoy bien o si necesito medicamentos para eso no importa quién sea que me lo haga lo que importa es el examen.

Title: Perla-5.docx

Descriptor Info: Age: Interview Location: Residence: Interviewer: Date of Interview:

Codes Applied: Intimacy

Excerpt Package: 350

Excerpt End: 1131

ALEXA: Aquí como es su experiencia en los centros de salud?

MUJER: Bueno las pocas veces que he venido la atención ha sido un poco lenta porque a veces los doctores tienen que hacer otras cosas antes de atender a los pacientes pero si me he sentido atendida con ellos solo que a veces necesitamos ampliar mas las salas porque en el momento en que los pacientes están con los médicos generales tal vez ellos necesitan su sala de ginecología algo más cerrado.

ALEXA: Entonces cree que falta un poco de privacidad?

MUJER: Si un poco.

ALEXA: Y cree que hay confianza con la privacidad de su información en los centros?

MUJER: Si, eso sí, sino que hay que mejorar a nivel de las salas tal vez necesitan ellos ampliar pero dada la situación que aquí es pequeño uno se tiene que acomodar.

Title: Mantica-2.docx

Descriptor Info: Age: Interview Location: Residence: Interviewer: Date of Interview:

Codes Applied: Intimacy

Excerpt Package: 8619

Excerpt End: 9030

ALEXANDRA: Usted piensa que las pruebas de pap son muy íntimas y el examen también, intimas emocional y físicamente

MUJER: Yo digo que sí porque uno se consciente como esta, es algo como no sé cómo explicarlo porque no tengo una idea como decírselo porque uno piensa cuando viene que si está bien o mal que si está enferma y cuando a uno le dan los resultados ahí es donde uno se da cuenta si está enfermo o no

Title: Perla-3.docx

Descriptor Info: Age: Interview Location: Residence: Interviewer: Date of Interview:

Codes Applied: Intimacy

Excerpt Package: 3713

Excerpt End: 4941

HANNAH: Si y como piensa es la actitud en general de las mujeres acerca de las pruebas de Papanicolaou?

MUJER: A muchas les gusta porque quieren tener salud porque salud es vida pero a otras no porque les da pena, porque dicen ahí que el doctor me va a revisar y eso no me gusta.

HANNAH: Porque es una cosa privada?

MUJER: Si pero pienso yo que en salud se debe de dejar la vergüenza porque te interesa tener tu salud y tu vida sana, entonces ni modo, aunque sea el doctor el que me vaya a revisar yo me dejo aunque me de vergüenza, aunque la cara la ponga a un lado pero yo eso pienso porque me ha tocado que hay doctores que toca que me revisen y aunque me de vergüenza tengo que hacerlo porque me interesa mi salud.

HANNAH: Se siente cómoda recibiendo una prueba de pap de un médico masculino?

MUJER: Si claro, que incomoda porque no es lo normal.

HANNAH: Y piensa que tiene el poder para pedir que prefiere una enfermera o medica femenina para realizar la prueba o no hay eso?

MUJER: Pues si necesito que me hagan el examen tengo que aceptar quien me lo haga, lo que importa es que me hagan la prueba para ver si estoy bien o si necesito medicamentos para eso no importa quién sea que me lo haga lo que importa es el examen.

Title: Mantica-3.docx

Descriptor Info: Age: Interview Location: Residence: Interviewer: Date of Interview:

Codes Applied: Intimacy

Excerpt Package: 5573

Excerpt End: 5691

HANNAH: No le importa si es mujer o varón?

MUJER: Eso ya no importa, uno pierde la vergüenza cuando tiene a sus hijos

Title: Perla-3.docx

Descriptor Info: Age: Interview Location: Residence: Interviewer: Date of Interview:

Codes Applied: Intimacy

Excerpt Package: 4432

Excerpt End: 4941

HANNAH: Se siente cómoda recibiendo una prueba de pap de un médico masculino?

MUJER: Si claro, que incomoda porque no es lo normal.

HANNAH: Y piensa que tiene el poder para pedir que prefiere una enfermera o medica femenina para realizar la prueba o no hay eso?

MUJER: Pues si necesito que me hagan el examen tengo que aceptar quien me lo haga, lo que importa es que me hagan la prueba para ver si estoy bien o si necesito medicamentos para eso no importa quién sea que me lo haga lo que importa es el examen.

Title: Poneloya-2.docx

Descriptor Info: Age: Interview Location: Residence: Interviewer: Date of Interview:

Codes Applied: Intimacy

Excerpt Package: 1492

Excerpt End: 2894

ALEXA: Y piensa usted que hay confianza con su información aquí en los centros de salud?

MUJER: Yo pienso que al menos para hacerme el pap como mujer, es con ella, con Aurorita porque me siento bien con ella pero hace un año me mandaron a hacer un estudio con respecto del pap, allá en el centro de salud pero fue con otra mujer con la que me siento en confianza con Mayrita la Licenciada, entonces si hay otros no, porque solo son esas dos personas con quienes yo me siento bien a que me hagan lo que me tienen que hacer como mujer

ALEXA: Entonces no se sentiría bien de recibir una prueba de pap de un médico varón?

MUJER: No, no sé cómo me iba a sentir porque quizás iba a ser primera vez aunque cuando iba a parir fue un varón el que me atendió aunque usted sabe que en el hospital no es igual que a un centro de salud porque como en los hospitales no están siempre los mismos, hoy está un grupo de varones después uno de mujeres nunca aquello es igual, mientras que en los centros de salud no es igual porque ahí siempre están las enfermeras y a la vez solo son mujeres las que mandan a los centros de salud por lo menos para mí es mejor, pero si no ni modo si hay un médico entonces si es varón aunque sea y me toca hacérmelo ni modo lo hago tengo que adaptarme porque no me voy a dejar morir solo porque es varón porque supongo yo que desde que él es médico tiene que saber y además ser privado.

Title: Poneloya-2.docx

Descriptor Info: Age: Interview Location: Residence: Interviewer: Date of Interview:

Codes Applied: Intimacy

Excerpt Package: 5135

Excerpt End: 5794

ALEXA: Y las mujeres que no se realizan las pruebas de pap porque cree que sucede?

MUJER: Algunas es por temor a tener vergüenza supongamos que yo ahorita tu hubieras entrado hubiese sido raro, a menos que Aurorita me hubiera dicho va a entrar ella es un alivio, pero si de pronto ibas a entrar entonces yo me iba a sentir como con pena pero me hubiera dado igual porque hoy la veo y mañana no y como si sos medico estas en todo el derecho pero siempre y cuando se pida el permiso si el paciente quiere que haya otra persona por lo menos a mí me tiene sin cuidado porque quizás aquella persona quiere ver algo que quiere saber, entonces es algo bueno para mí.

Title: Santa Ana-1.docx

Descriptor Info: Age: Interview Location: Residence: Interviewer: Date of Interview:

Codes Applied: Intimacy

Excerpt Package: 2815

Excerpt End: 3033

SARA: Se sentiría cómoda recibiendo una prueba de pap de un médico varón?

MUJER: Pues la verdad preferiría una doctora

SARA: Y tiene la opción de elegir quien le hace la prueba

MUJER: Pues si sería bueno de elegir aquí

Title: Mantica-2.docx

Descriptor Info: Age: Interview Location: Residence: Interviewer: Date of Interview:

Codes Applied: Intimacy

Excerpt Package: 3153

Excerpt End: 3675

ALEXANDRA: Y quien le realizo la prueba de pap, una doctora o doctor o quién?

MUJER: Una doctora

ALEXANDRA: Y su actitud hizo que se sintiera cómoda

MUJER: A mí me gusta que me atienda una doctora porque con una mujer uno se siente más cómodo

ALEXANDRA: Y fuera un médico varón no se sentiría cómoda

MUJER: No, a menos que fuera a dar a luz no tengo otra opción

ALEXANDRA: Se sentiría cómoda de hablar del cáncer cervical o algo como eso con un médico varón

MUJER: No tanto, sería mejor con una doctora, familia y mi madre

Title: Mantica-6.docx

Descriptor Info: Age: Interview Location: Residence: Interviewer: Date of Interview:

Codes Applied: Intimacy

Excerpt Package: 2492

Excerpt End: 3570

ALEXANDRA: Y como es la actitud de las enfermeras que realizan las pruebas de pap?

MUJER: Su actitud?

ALEXANDRA: Sí, su actitud hace que se sientan cómodas o incomodas?

MUJER: Cómodas porque somos mujeres, ellas atienden muy bien y hacen muy bien todo

ALEXANDRA: Entonces si fuera un enfermero o un médico varón no se sentiría cómoda

MUJER: Depende, si se hace acompañar de una mujer es mas cómodo, hacen igual el trabajo pero uno está acostumbrado que sea una mujer por que así uno se desplaza mejor

ALEXANDRA: Es muy íntimo emocionalmente o físicamente una prueba de pap

MUJER: Emocionalmente es mejor con una mujer porque un varón es más intimidante y uno no se relaja lo suficiente, mientras que una mujer ya sabemos cómo son nuestras partes pero el hombre solo conoce sus partes y lo que ha estudiado sobre nosotras

ALEXANDRA: Y se siente cómoda hablando de los paps o del cáncer cervical con un médico varón o solamente con las mujeres?

MUJER: Si me siento cómoda, hablar, pero ya de revisarse es otra cosa pero abiertamente hablar de nuestro cuerpo con un varón es normal

Title: Los Leches-3.docx

Descriptor Info: Age: Interview Location: Residence: Interviewer: Date of Interview:

Codes Applied: Intimacy

Excerpt Package: 2693

Excerpt End: 2791

SARA: Y se sentiría cómoda recibiendo una prueba de pap de un médico masculino?

MUJER: No, no y no

Title: Subtiava-2.docx

Descriptor Info: Age: Interview Location: Residence: Interviewer: Date of Interview:

Codes Applied: Intimacy

Excerpt Package: 1906

Excerpt End: 2157

HANNAH: Bueno, se sentiría cómoda recibiendo una prueba de Pap con un médico masculino?

MUJER: Por lo general a mí siempre me han atendido del sexo femenino y en verdad prefiero siga siendo así y creo que las mujeres atienden más y uno se siente mejor

Title: Perla-4.docx

Descriptor Info: Age: Interview Location: Residence: Interviewer: Date of Interview:

Codes Applied: Intimacy

Excerpt Package: 6446

Excerpt End: 7251

HANNAH: Y cómo piensa es la actitud de las mujeres en la comunidad acerca del cáncer de cuello uterino?

MUJER: Bueno lo que pasa es que siempre hay mujeres que les da pena andar en el medico y eso se da cuando los maridos andan detrás de las mujeres porque no quieren que ni los médicos las miren siempre se da eso, yo tengo una amiga que apenas fue al médico y recién se dio cuenta que tiene el virus y su marido se molestó con ella porque se dejó ver de un hombre, entonces yo le dije que le dijera que le pague para que vaya donde una doctora

HANNAH: Ella no quiere recibir una prueba de pap de un médico masculino y por eso no quiere venir?

MUJER: Exactamente pero ella ya se lo hizo y ella se lo comento a su marido y parece que se molestó, que locura le dije yo, pero bueno cada quien con sus ideas

Title: Los Leches-2.docx

Descriptor Info: Age: Interview Location: Residence: Interviewer: Date of Interview:

Codes Applied: Intimacy

Excerpt Package: 1991

Excerpt End: 2141

HANNAH: Se sentiría cómoda recibiendo una prueba de pap de un médico masculino o prefiere de una mujer?

MUJER: De una femenina se siente más tranquila

Title: Mantica-1.docx

Descriptor Info: Age: Interview Location: Residence: Interviewer: Date of Interview:

Codes Applied: Intimacy

Excerpt Package: 2936

Excerpt End: 3200

Sara: Y se sentiría cómoda recibiendo una prueba de pap de un médico masculino?

Mujer: A mí me gusta que me atienda una mujer, cuando estaba embarazada en el hospital en emergencia ahí habían un montón de doctores y todos me hicieron el pap, no podía pedir gusto.

Title: Perla-5.docx

Descriptor Info: Age: Interview Location: Residence: Interviewer: Date of Interview:

Codes Applied: Intimacy

Excerpt Package: 9746

Excerpt End: 9978

ALEXA: Se sentiría cómoda recibiendo una prueba de pap de un médico varón?

MUJER: Bueno yo ya me lo he hecho con un varón y pues todo ha sido bastante profesional en ese aspecto, nada de morbo a como dicen y todo con ética, ya listo

**Pap Smear**

Title: Mantica-4.docx

Descriptor Info: Age: Interview Location: Residence: Interviewer: Date of Interview:

Codes Applied: Pap smear

Excerpt Package: 3086

Excerpt End: 3203

HANNAH: Y antes de su diagnóstico cuantas veces al año recibía las pruebas de Papanicolaou?

MUJER: Dos veces cada año

Title: SantaAna-4.docx.docx

Descriptor Info: Age: Interview Location: Residence: Interviewer: Date of Interview:

Codes Applied: Pap smear

Excerpt Package: 3053

Excerpt End: 3833

ALEXANDRA: Y usted se ha realizado alguna prueba de Pap?

MUJER: Papanicolaou? Si, como tres me he hecho, hoy me lo iba a hacer pero ando un poquito rápido porque mi marido va para la pesca y no me puedo dilatar, Dios quiera y esta semana que venga voy a hacérmelo para ver como estoy, me gustaría hacerlo.

ALEXANDRA: Por cuales razones decide usted hacerse el Pap?

MUJER: Bueno hay que hacerse el pap porque usted sabe que más adelante uno viene sintiéndose los síntomas de algo y por eso mejor antes de eso hay que hacerse lo primero porque más adelante uno no sabe y yo digo que hay personas que hasta que miran la enfermedad avanzada buscan y uno debe buscar solución antes de que la enfermedad se introduzca en el cuerpo de uno por eso es que me gusta a mi hacerme el pap aquí

Title: Santa Ana-3.docx

Descriptor Info: Age: Interview Location: Residence: Interviewer: Date of Interview:

Codes Applied: Pap smear

Excerpt Package: 1626

Excerpt End: 2460

HANNAH: Y usted me dijo que recibe una prueba de pap cada seis meses?

MUJER: Si

HANNAH: Cuando empezó a recibir las pruebas de Pap?

MUJER: Bueno empecé que mi primer pap fue a los quince años

HANNAH: Bueno, y usted sabe las razones de porque una mujer debe recibir una prueba de pap? Usted hablo un poco de eso antes.

MUJER: Si

HANNAH: Cual piensa es la actitud en general de las mujeres acerca de las pruebas de pap?

MUJER: Bueno, yo siempre las invito a que se hagan la prueba de pap para ver si tenemos el cáncer de cuello uterino ya que muchas mujeres estamos padeciendo de esto, a veces hay algunas mujeres que piensan que el pap es para esas mujeres que han tenido muchas parejas entonces decimos que no, que el pap es para toditas para tener un mejor cuido tanto para las jóvenes como para la mujer ya adulta, es muy importante

Title: Santa Ana-3.docx

Descriptor Info: Age: Interview Location: Residence: Interviewer: Date of Interview:

Codes Applied: Pap smear

Excerpt Package: 106

Excerpt End: 699

HANNAH: Perfecto y dígame un poco sobre su experiencia con el cuidado de salud?

MUJER: Bueno mi experiencia con la salud, es que ahorita estamos combatiendo el cáncer de mama, la campaña es revísate, tócate para que no te pase, que hay que hacerse el autoexamen de mamas, que hay que estarse revisando que si no tienen pelotas, sino se les brotan las manos también, entonces ese es uno de los exámenes que yo me hago cuidadosamente, me reviso me hago mi pap a tiempo cada seis meses y si siento que tengo una media picazón me voy inmediatamente al centro de salud para que me den tratamiento.

Title: Subtiava-2.docx

Descriptor Info: Age: Interview Location: Residence: Interviewer: Date of Interview:

Codes Applied: Pap smear

Excerpt Package: 1224

Excerpt End: 1905

HANNAH: Ah bueno, una vez cada año usted recibe una prueba de Papanicolaou?

MUJER: Si, bueno eso nos recomiendan y al menos yo así lo hago año con año porque me dicen que es bueno y así pueda rendirle a mis hijos y por eso yo me las hago y ellas dicen es anualmente para también detectar cualquier tipo de cáncer

HANNAH: Una vez cada año, y puede hablar acerca de las razones del porque quiere recibir una prueba de Papanicolaou cada año?

MUJER: Las razones bueno a mí me han dicho que ahí sale plasmado que tiene de enfermedad se tiene y pienso yo que si se está revisando constantemente así si tienes algo te lo puedes tratar a tiempo, así yo creo que son las principales razones

Title: Mantica-4.docx

Descriptor Info: Age: Interview Location: Residence: Interviewer: Date of Interview:

Codes Applied: Pap smear

Excerpt Package: 1391

Excerpt End: 1875

HANNAH; Como fue su tratamiento después del diagnóstico, recibe mucho?

MUJER: Pues no, medicamentos no recibo, solo son los exámenes que el doctor me hace, por ejemplo los quemados que me hacía y después del cono no me ha hecho nada no me han revisado desde la fecha que me lo hice y eso fue el cuatro de noviembre del dos mil quince, hasta ahora me revisaran porque me hare un Papanicolaou de nuevo

HANNAH: Y ahora cuantas veces al año recibe un Papanicolaou?

MUJER: Cada seis meses

Title: Santa Ana-1.docx

Descriptor Info: Age: Interview Location: Residence: Interviewer: Date of Interview:

Codes Applied: Pap smear

Excerpt Package: 2478

Excerpt End: 2591

SARA: Y usted sabe el objetivo de las prueba de pap

MUJER: Se trata de detectar algún tipo de enfermedad o cáncer

Title: Los Leches-3.docx

Descriptor Info: Age: Interview Location: Residence: Interviewer: Date of Interview:

Codes Applied: Pap smear

Excerpt Package: 1879

Excerpt End: 2373

SARA: Ha tenido una prueba de Papanicolaou alguna vez, en la matriz, es un examen pélvico?

MUJER: Si

SARA: Y cuál fue el contexto estaba embarazada?

MUJER: Si, lo estaba

SARA: Cuando recibió la prueba de pap?

MUJER: A mí lo único que me sale en la prueba es que tengo inflamado los ovarios

SARA: Porqué recibió una prueba de pap?

MUJER: Porque me la he hecho vengo y me lo hago porque me duele mucho los ovarios

SARA: En su matriz?

MUJER: Si y no sé porque, ya que solo me sale inflamación leve

Title: Mantica-5.docx

Descriptor Info: Age: Interview Location: Residence: Interviewer: Date of Interview:

Codes Applied: Pap smear

Excerpt Package: 1802

Excerpt End: 1939

HANNAH: Y usted sabe el objetivo o las razones de porque hacerse una prueba de pap?

MUJER: Si, por las enfermedades, el cáncer y todo eso

Title: Mantica-2.docx

Descriptor Info: Age: Interview Location: Residence: Interviewer: Date of Interview:

Codes Applied: Pap smear

Excerpt Package: 1881

Excerpt End: 2476

ALEXANDRA: Ha oído hablar de la prueba de Papanicolaou alguna vez y se ha realizado alguna

MUJER: Sí, sí.

ALEXANDRA: Y se recomienda aquí los centros de salud

MUJER: Sí, yo aquí me lo hago

ALEXANDRA: Y por cuales razones usted se realiza un pap

MUJER: Porque eso es algo que la mujer se debe hacer cada seis meses es parte de ver como estar uno con su salud para ver si uno no tiene problemas

ALEXANDRA: Y cree que todas las chicas adolescentes deben realizarse un pap si tienen relaciones sexuales

MUJER: Yo digo que sí porque ahora a como están las enfermedades uno debe de tener mucho cuidado

Title: Perla-3.docx

Descriptor Info: Age: Interview Location: Residence: Interviewer: Date of Interview:

Codes Applied: Pap smear

Excerpt Package: 10115

Excerpt End: 10442

HANNAH: Y antes de su diagnóstico ella había recibido pruebas de pap periódicamente o con qué frecuencia?

MUJER: Uhm, yo creo que no, antes de que le pasara eso yo creo que no se hacía, paso un tiempo en que ella no se lo hacía entonces me imagino que por eso no se daba cuenta y resulto eso cuando ella se sintió los síntomas.

Title: Mantica-5.docx

Descriptor Info: Age: Interview Location: Residence: Interviewer: Date of Interview:

Codes Applied: Pap smear

Excerpt Package: 3627

Excerpt End: 4254

HANNAH: Piensa que el cáncer de cuello uterino es algo muy común que puede afectarle en un futuro o es algo muy raro?

MUJER: Yo digo que es algo muy grave porque no nos cuidamos, digo no nos cuidamos porque yo no estoy pendiente de mis exámenes, a veces digo hoy no me lo hare será hasta el otro mes y ahí no me cuido.

HANNAH: Después de lo de su amiga piensa que le afecta e influye a que se haga ms pruebas de paps?

MUJER: Ah sí, ahora lo más que tardo son ocho meses en hacerme el examen y me hago uno aquí en el Centro de Salud y otro privado

HANNAH: Y lo hace porque sabe que es una cosa muy importante?

MUJER: Si, así es

Title: Mantica-4.docx

Descriptor Info: Age: Interview Location: Residence: Interviewer: Date of Interview:

Codes Applied: Pap smear

Excerpt Package: 1791

Excerpt End: 1875

HANNAH: Y ahora cuantas veces al año recibe un Papanicolaou?

MUJER: Cada seis meses

Title: Los Leches-2.docx

Descriptor Info: Age: Interview Location: Residence: Interviewer: Date of Interview:

Codes Applied: Pap smear

Excerpt Package: 1449

Excerpt End: 1768

HANNAH: Con que frecuencia recibe las pruebas de pap?

MUJER: Cada seis meses, si porque es bueno estarse chequeando a ver cómo va uno en su salud

HANNAH: Puede hablar un poco acerca de las razones porque quiere recibir una prueba cada seis meses?

MUJER: Por las infecciones por todas las cosas que ahora la mujer recibe

Title: Mantica-4.docx

Descriptor Info: Age: Interview Location: Residence: Interviewer: Date of Interview:

Codes Applied: Pap smear

Excerpt Package: 3086

Excerpt End: 3203

HANNAH: Y antes de su diagnóstico cuantas veces al año recibía las pruebas de Papanicolaou?

MUJER: Dos veces cada año

Title: Mantica-6.docx

Descriptor Info: Age: Interview Location: Residence: Interviewer: Date of Interview:

Codes Applied: Pap smear

Excerpt Package: 0

Excerpt End: 1101

ALEXANDRA: Entonces, primeramente dígame acerca de su experiencia con el cuidado de salud, en los centros de salud y los médicos?

MUJER: El centro de salud siempre está promoviendo que uno esté pendiente de su higiene principalmente a las mujeres, que esté al tanto con sus paps, sus controles de embarazo que a sus allegados que estén enfermos los traigan están al pendiente que siempre puedan atender a las personas que están enfermas y nos brindan un servicio del que nosotros hacemos uso, siempre nos atienden amablemente y estamos contentos de que siempre estén pendientes de atender a las personas siempre que hay problemas, y pendientes de los problemas que tengamos las mujeres aun ya obteniendo los resultados de sus paps controles de embarazo y la salud de los niños

ALEXANDRA: Y usted también puede recibir la salud sexual, tratamiento y consejos aquí?

MUJER: Si aquí nos ayudan con la salud sexual de la mujer activa ya sea protección por medio del pap, una vez haciéndote el pap si te dan resultado y te sale algo te dan tratamiento y te siguen la secuencia hasta que sales de tu problema

Title: Subtiava-2.docx

Descriptor Info: Age: Interview Location: Residence: Interviewer: Date of Interview:

Codes Applied: Pap smear

Excerpt Package: 2788

Excerpt End: 3296

HANNAH: Y cómo piensa que es la actitud y conocimiento de las mujeres con respecto a las pruebas de pap?

MUJER: Bueno al menos yo le presto mucha importancia porque sé que es para mi salud y si yo no cuido mi cuerpo nadie más lo hará, tengo que hacerlo por mis hijos y siempre se recomienda cada vez que vengo me preguntan si me hice la prueba que recuerde hacerla anualmente que así si tengo una enfermedad se detecta a tiempo y se puede combatir, así que pienso es importante para tener salud en su cuerpo.

Title: Mantica-6.docx

Descriptor Info: Age: Interview Location: Residence: Interviewer: Date of Interview:

Codes Applied: Pap smear

Excerpt Package: 1748

Excerpt End: 1982

ALEXANDRA: Y usted ha oído de la prueba de pap antes de hoy]?

MUJER: Sí, actualmente las mujeres debemos estar pendientes con nuestro pap mensualmente

ALEXANDRA: Entonces usted se ha realizado pruebas de pap?

MUJER: Sí, me hago mi pap

Title: Mantica-3.docx

Descriptor Info: Age: Interview Location: Residence: Interviewer: Date of Interview:

Codes Applied: Pap smear

Excerpt Package: 2275

Excerpt End: 2475

HANNAH: Y cuántas veces al año se realiza una prueba de Papanicolaou?

MUJER: Bueno, yo vengo cada dos o tres meses a hacérmelas le podría decir que vengo para saber cómo estoy y ver qué resultados hay

Title: Perla-2.docx

Descriptor Info: Age: Interview Location: Residence: Interviewer: Date of Interview:

Codes Applied: Pap smear

Excerpt Package: 2774

Excerpt End: 3100

ALEXANDRA: Y como se dio cuenta de que tenía ese cáncer?

MUJER: Haciéndose el Pap

ALEXANDRA: Entonces usted se hace regularmente el Pap?

MUJER: Si así es

ALEXANDRA: Cuales son sus preocupaciones acerca del Pap?

MUJER: Contraer el cáncer

ALEXANDRA: Y por cuales razones se hace el pap?

MUJER: Para detectar el cáncer a tiempo

Title: Subtiava-1.docx

Descriptor Info: Age: Interview Location: Residence: Interviewer: Date of Interview:

Codes Applied: Pap smear

Excerpt Package: 4808

Excerpt End: 5771

HANNAH: Pruebas de Papanicolaou?

MUJER: Bueno, dos veces así, pero eso se debe de hacer cada seis meses o al año.

HANNAH: Solo dos veces cada año, usted lo hace?

MUJER: Aja pero la verdad es que yo solo dos veces me lo he hecho y la última vez que ni he ido a ver el resultado

HANNAH: Oh usted, solo dos veces en su vida ha recibido una prueba de pap?

MUJER: Si

HANNAH: Y piensa que no necesita más porque?

MUJER: No, si es necesario pero no he tenido tiempo por el trabajo pero si voy a dedicar un dia para ir donde la doctora ver los resultados pero yo pienso y siento que estoy bien.

HANNAH: Pero si se sintiera mal?

MUJER: Yo fuera rápidamente de una vez.

HANNAH: Pero porque usted siente que está bien ahora piensa que no necesita una prueba de pap?

MUJER: Si pero voy a ir.

HANNAH: Oh que bueno, y porque es que usted decidió realizarse la prueba de pap en el pasado?

MUJER: Para chequear mi salud que este todo bien y normal y sentirme segura de mi misma.

Title: Mantica-5.docx

Descriptor Info: Age: Interview Location: Residence: Interviewer: Date of Interview:

Codes Applied: Pap smear

Excerpt Package: 1032

Excerpt End: 1305

HANNAH: Ha recibido una prueba de Papanicolaou alguna vez?

MUJER: Si

HANNAH: Y cuantas veces se la ha hecho?

MUJER: Infinidades de veces me lo han hecho, cada ocho meses me lo hago aquí

HANNAH: Cada ocho meses y durante sus embarazos se los hizo también?

MUJER: Si, también

Title: Mantica-6.docx

Descriptor Info: Age: Interview Location: Residence: Interviewer: Date of Interview:

Codes Applied: Pap smear

Excerpt Package: 6436

Excerpt End: 7026

ALEXANDRA: Y que tan preocupada está usted de las enfermedades de transmisión sexual?

MUJER: La preocupación hoy en día ya es con responsabilidad y con protección sobre todo

ALEXANDRA: Y cuales precauciones toma usted para mantenerse su salud y seguridad?

MUJER: En mi caso con el pap pero las personas que lo hacen irresponsablemente creo que deberían de usar el preservativo porque no tienen un control de asistir a un centro de salud más que todo en la juventud en el sexo prematuro y en mi caso asisto al centro de salud y estoy en mi chequeo y así me doy cuenta si tengo o no problemas

Title: Mantica-6.docx

Descriptor Info: Age: Interview Location: Residence: Interviewer: Date of Interview:

Codes Applied: Pap smear

Excerpt Package: 8818

Excerpt End: 9050

ALEXANDRA: Es difícil recibir una prueba de pap?

MUJER: No, no están difícil

ALEXANDRA: Por cuales razones se haría una prueba de pap?

MUJER: Por salud, para saber si tengo alguna enfermedad, para mirarme a tiempo y tratarme también

Title: Perla-5.docx

Descriptor Info: Age: Interview Location: Residence: Interviewer: Date of Interview:

Codes Applied: Pap smear

Excerpt Package: 7090

Excerpt End: 7311

ALEXA: Y cuáles fueron sus razones para realizarse la prueba?

MUJER: Porque yo tengo veinte seis años y como tengo relaciones, entonces era necesario realizarme la prueba por cualquier cosa, por alguna infección o cáncer.

Title: Mantica-1.docx

Descriptor Info: Age: Interview Location: Residence: Interviewer: Date of Interview:

Codes Applied: Pap smear

Excerpt Package: 8237

Excerpt End: 8684

Sara: Que hace para mantener la salud y seguridad contra enfermedades de transmisión sexual?

Mujer: Que hago yo? Pues voy al centro de salud y me hago el pap, pero como actualmente no tengo pareja y así estoy mejor y a como son los hombres ahora con quien estuvieron o no y yo tengo como que miedo, y todo eso es lo único que se, y como ya no visito ese proyecto al que iba ya solo se lo que dicen en el centro de salud y veo los carteles que hay

Title: Los Leches-1 (1).docx

Descriptor Info: Age: Interview Location: Residence: Interviewer: Date of Interview:

Codes Applied: Pap smear

Excerpt Package: 2281

Excerpt End: 2518

Sara: Have you ever have a Pap test? How was it? Why did you received it? Was here?

Woman: Yes, I have it, was here, (I don’t understand), I think that is important because trough that we can prevent some disease, and I don’t know more.

Title: Poneloya-1.docx

Descriptor Info: Age: Interview Location: Residence: Interviewer: Date of Interview:

Codes Applied: Pap smear

Excerpt Package: 2081

Excerpt End: 2231

ALEXANDRA: Y ha realizado una prueba de pap?

MUJER: No, es que yo no tengo marido, yo por eso le dije a ella que participaba ´pero que no tengo marido

Title: Los Leches-3.docx

Descriptor Info: Age: Interview Location: Residence: Interviewer: Date of Interview:

Codes Applied: Pap smear

Excerpt Package: 920

Excerpt End: 1353

SARA: Dígame su experiencia acerca de la salud sexual y educación sexual? Ha recibido educación sexual de los centros o de su familia?

MUJER: En qué forma dice usted

SARA: Sobre las infecciones sexuales, sobre el sexo, sobre la pubertad?

MUJER: Bueno lo que yo he padecido mucho es humedad y el pap siempre me ha salido bien aquí y como yo trabajo en Yazaki he ido a la clínica y ahí también me lo han hecho y me sale igual que aquí

Title: Perla-4.docx

Descriptor Info: Age: Interview Location: Residence: Interviewer: Date of Interview:

Codes Applied: Pap smear

Excerpt Package: 96

Excerpt End: 433

HANNAH: Bueno y para empezar dígame su experiencia con los cuidados de salud?

MUJER: Con el cuidado de salud, bueno yo siempre vengo a hacer mis controles del pap porque tengo mis antecedentes de un virus y siempre me están controlando, el ginecólogo siempre, constantemente y el control de la presión porque ya es algo crónico nada más

Title: Santa Ana-2.docx

Descriptor Info: Age: Interview Location: Residence: Interviewer: Date of Interview:

Codes Applied: Pap smear

Excerpt Package: 3249

Excerpt End: 3990

ALEXANDRA: Alguna vez se ha realizado una prueba de Papanicolaou?

MUJER: Si, ahorita aquí lo ando.

ALEXANDRA: Oh que bien, y por cuales razones decide hacerse una prueba de pap?

MUJER: Bueno me gustaría porque uno aquí sale si tiene algún problema como cáncer o inflamación o algo para uno tratárselo porque importante, porque por ejemplo si a alguien le sale una inflamación en un ovario ellos tienen que tener los tratamientos para cada uno al igual si fuese una enfermedad venérea por lo mismo para saber, a veces digo yo y me pongo que no quiero, porque pienso en si me saldrá alguna enfermedad, mejor si la tengo ahí que este y me voy a morir sin saber pero no, en realidad es muy importante porque la salud de uno es lo más importante

Title: Perla-2.docx

Descriptor Info: Age: Interview Location: Residence: Interviewer: Date of Interview:

Codes Applied: Pap smear

Excerpt Package: 6545

Excerpt End: 6769

MUJER: Que se muera uno de cualquier enfermedad

ALEXANDRA: Y qué hace usted para mantener su salud y prevenir esas enfermedades?

MUJER: Estarme chequeando de vez en cuando. Cada seis meses que hay que estarse haciendo el pap

Title: Poneloya-2.docx

Descriptor Info: Age: Interview Location: Residence: Interviewer: Date of Interview:

Codes Applied: Pap smear

Excerpt Package: 9457

Excerpt End: 10091

ALEXA: Y cuando piensa en cáncer de cuello uterino en que piensa usted?

MUJER: Lo mismo que vuelvo y repito que somos nosotros los que debemos cuidarnos y detectarnos eso que tenemos porque si yo ya tengo un poco que me va a salir entonces tendría posibilidades para que el cáncer no siga por eso es que ahí se inicia detectar con el pap, es que el pap es lo más esencial para nosotras las mujeres porque ahí donde nos detectan las enfermedades que tenemos, yo puedo tener cuatro o cinco años de no tener relaciones sexuales pero yo siempre tengo que hacerme el pap porque es algo que uno anda dentro oculto si tuve alguna inflamación

Title: Mantica-4.docx

Descriptor Info: Age: Interview Location: Residence: Interviewer: Date of Interview:

Codes Applied: Pap smear Care

Excerpt Package: 606

Excerpt End: 828

HANNAH: Y hace cuanto supo que tiene cáncer?

MUJER: En el año 2014

HANNAH: Por medio de qué medida se dio cuenta por una prueba de Papanicolaou?

MUJER: Si Por el Papanicolaou me di cuenta que había un problema en la matriz

Title: Santa Ana-1.docx

Descriptor Info: Age: Interview Location: Residence: Interviewer: Date of Interview:

Codes Applied: Pap smear Cervical cancer

Excerpt Package: 4322

Excerpt End: 4742

SARA: Que piensa cuando escucha la palabra cáncer

MUJER: Bueno, que es una enfermedad que por eso hay que hacerse el pap para detectar si hay algún cáncer para atenderlo a tiempo

SARA; Como es el conocimiento y actitud de la comunidad con el cáncer de cuello uterino? Es igual que con el virus de papiloma humano

MUJER: Sí, es diferente pero verdad que por eso mucha gente se hace los paps para detectarse enfermedades

Title: Santa Ana-2.docx

Descriptor Info: Age: Interview Location: Residence: Interviewer: Date of Interview:

Codes Applied: Pap smear Fear

Excerpt Package: 3991

Excerpt End: 4496

ALEXANDRA: Y las mujeres que deciden no realizarse una prueba de pap, porque razones cree usted que lo hacen?

MUJER: Yo digo o no sé, que tal vez es por pena de ellas mismas o tal vez porque no quieran darse cuenta del cual es su enfermedad que ellas tengan porque yo por lo menos al principio así era, yo decía “no me lo hago” dilate cinco años que no me lo hice y las enfermeras me decían hágaselo, pero en realidad es muy importante porque hay miles de enfermedades y uno a través de esto se da cuenta.

Title: Perla-3.docx

Descriptor Info: Age: Interview Location: Residence: Interviewer: Date of Interview:

Codes Applied: Pap smear HPV

Excerpt Package: 3138

Excerpt End: 3712

HANNAH: Usted se ha realizado una prueba de Papanicolaou alguna vez?

MUJER: Si, siempre me lo hago, cada año.

HANNAH: Una vez cada año?

MUJER: Si, cada año

HANNAH: Bueno y porque decide usted hacerse una prueba de Pap?

MUJER: Bueno, porque así por lo menos me doy cuenta si no tengo alguna enfermedad que tal vez no sienta y entonces ese examen es para hacerle una prueba a la mujer para ver si no tiene una enfermedad de transmisión sexual, la enfermedad del papiloma humano, ya me entiende muchas enfermedades vaginales por la transmisión sexual o por relaciones sexuales.

Title: Perla-3.docx

Descriptor Info: Age: Interview Location: Residence: Interviewer: Date of Interview:

Codes Applied: Pap smear HPV

Excerpt Package: 3138

Excerpt End: 3712

HANNAH: Usted se ha realizado una prueba de Papanicolaou alguna vez?

MUJER: Si, siempre me lo hago, cada año.

HANNAH: Una vez cada año?

MUJER: Si, cada año

HANNAH: Bueno y porque decide usted hacerse una prueba de Pap?

MUJER: Bueno, porque así por lo menos me doy cuenta si no tengo alguna enfermedad que tal vez no sienta y entonces ese examen es para hacerle una prueba a la mujer para ver si no tiene una enfermedad de transmisión sexual, la enfermedad del papiloma humano, ya me entiende muchas enfermedades vaginales por la transmisión sexual o por relaciones sexuales.

Title: Los Leches-2.docx

Descriptor Info: Age: Interview Location: Residence: Interviewer: Date of Interview:

Codes Applied: Pap smear HPV Cervical cancer

Excerpt Package: 1595

Excerpt End: 1990

HANNAH: Puede hablar un poco acerca de las razones porque quiere recibir una prueba cada seis meses?

MUJER: Por las infecciones por todas las cosas que ahora la mujer recibe

HANNAH: Y puede hablar acerca de las infecciones, sabe los nombres?

MUJER: Uhm yo no he tenido ninguna infección grave solo la inflamación en los ovaros y matriz humedad pero de todas esas infecciones no me he dado cuenta

Title: Poneloya-2.docx

Descriptor Info: Age: Interview Location: Residence: Interviewer: Date of Interview:

Codes Applied: Pap smear Promiscuity

Excerpt Package: 4385

Excerpt End: 5134

ALEXA: Y por cuales razones recibiría usted una prueba de pap?

MUJER: Porque en el pap ahí se encuentra muchas enfermedades que podemos tener las mujeres, ahí nos detectan si tenemos cáncer, si tenemos infección transmitidas por las relaciones o porque me puse algo o más de algún parasito que uno recibe para mi es excelente que uno se haga el pap mas en nosotras las mujeres de edad avanzada porque es más donde nos da el cáncer en las mujeres adultas porque como nosotras decimos que no tenemos pareja para que vamos a ir pero no, aunque no las tengamos, ni tengamos relaciones sexuales uno tiene infecciones que a uno el cuerpo le da, siempre el pap es importante aquí y donde sea porque ahí es donde se detecta el cáncer uterino de las mujeres.

Title: Subtiava-2.docx

Descriptor Info: Age: Interview Location: Residence: Interviewer: Date of Interview:

Codes Applied: Pap smear Support

Excerpt Package: 5127

Excerpt End: 5474

HANNAH: Como es el conocimiento de las mujeres de la comunidad acerca de esta enfermedad?

MUJER: Por lo menos cada vez que yo vengo aquí ya sea por mi o con mi hijo, ellos me preguntan si ya me hice el pap si ya me chequee que andas y asi uno se va dando cuenta poco a poco de lo que significa todo, desde los exámenes que te haces y todo lo demás

**Physical Comfort**

Title: Mantica-2.docx

Descriptor Info: Age: Interview Location: Residence: Interviewer: Date of Interview:

Codes Applied: Physical comfort

Excerpt Package: 2477

Excerpt End: 2744

ALEXANDRA: Y es doloroso la prueba de pap

MUJER: Bueno en mí caso, le podría decir que solo me ha dolido una vez porque tenía una infección pero las otras veces no porque los doctores te dicen que si tienes una infección te duele sino no entonces a mí no me ha dolido

Title: Perla-2.docx

Descriptor Info: Age: Interview Location: Residence: Interviewer: Date of Interview:

Codes Applied: Physical comfort

Excerpt Package: 3543

Excerpt End: 3642

ALEXANDRA: Y ella está preocupada acerca de la prueba? Porque?

MUJER: Porque ella no sabe si duele

Title: Los Leches-1 (1).docx

Descriptor Info: Age: Interview Location: Residence: Interviewer: Date of Interview:

Codes Applied: Physical comfort

Excerpt Package: 3944

Excerpt End: 4042

Sara: Would you feel comfortable emotionally and physically if you receive a pap test?

Woman: Yes.

Title: Los Leches-2.docx

Descriptor Info: Age: Interview Location: Residence: Interviewer: Date of Interview:

Codes Applied: Physical comfort

Excerpt Package: 2239

Excerpt End: 2305

HANNAH: Se sentiría cómoda recibiendo una prueba de pap?

MUJER: Si

Title: Mantica-5.docx

Descriptor Info: Age: Interview Location: Residence: Interviewer: Date of Interview:

Codes Applied: Physical comfort

Excerpt Package: 1617

Excerpt End: 1801

HANNAH: Quince días, ok, piensa que las pruebas de pap son dolorosas o incomodas?

MUJER: No, yo nunca he sentido dolor, a mí siempre mis Pap me los ha hecho la Doctora no la enfermera.

Title: Mantica-1.docx

Descriptor Info: Age: Interview Location: Residence: Interviewer: Date of Interview:

Codes Applied: Physical comfort

Excerpt Package: 2842

Excerpt End: 2935

Sara: Se sentiría cómoda recibiendo una prueba de pap físicamente y emocionalmente?

Mujer: Si

Title: Los Leches-3.docx

Descriptor Info: Age: Interview Location: Residence: Interviewer: Date of Interview:

Codes Applied: Physical comfort

Excerpt Package: 2374

Excerpt End: 2692

SARA: Quien le ha realizado la prueba de pap?

MUJER: Aquí solo me la ha hecho la enfermera que se llama Marlene aquí en el centro

SARA: Cuáles son sus preocupaciones con la prueba de pap es dolorosa, piensa o que es intima?

MUJER: Depende porque si le duele porque hay enfermeras que es suave la mano hay otras que no

Title: Santa Ana-1.docx

Descriptor Info: Age: Interview Location: Residence: Interviewer: Date of Interview:

Codes Applied: Physical comfort

Excerpt Package: 2592

Excerpt End: 2814

SARA: Tiene preocupaciones con las pruebas de pap, cree que causan molestias a corto plazo o algo así

MUJER: No para nada

SARA: Se sentiría cómoda emocional y físicamente recibiendo una prueba de pap

MUJER: Si claro que si

Title: Subtiava-2.docx

Descriptor Info: Age: Interview Location: Residence: Interviewer: Date of Interview:

Codes Applied: Physical comfort Pap smear Time Intimacy

Excerpt Package: 6117

Excerpt End: 7120

HANNAH: Y piensa que las mujeres saben los beneficios de las pruebas de pap y quieren recibirlas?

MUJER: Bueno, no todas se vienen a hacer el pap porque piensan que no es importante porque no tienen el conocimiento necesario para poder saber si al hacérselo pueden prevenir una enfermedad importante, solo saben decir “hay no ir a chequearme, no me gusta peor que me estén revisando mis partes” pero es algo que solo es un momento y aunque duele un poquito porque te arrancan un pedacito pero es mejor eso que después tener algo que no se podrá curar, pero a pesar hay mujeres que no les gusta venir a revisarse informarse o solo vienen y no leen los murales y se aburren entonces dicen “es que es aburrido y paso todo el día” pero no, yo pienso que es algo importante porque invertir un poco de tu tiempo es bueno en vez de estar todo el tiempo enfermo y no poderte curar es algo muy triste por eso para mí es algo muy importante porque es por la salud y el quererse uno mismo que es lo más importante.

**Private Care**

Title: Perla-5.docx

Descriptor Info: Age: Interview Location: Residence: Interviewer: Date of Interview:

Codes Applied: Private care

Excerpt Package: 1132

Excerpt End: 3588

ALEXA: Y ha tenido alguna mala experiencia aquí en el centro?

MUJER: No, todavía no, Gracias a Dios no me han atendido mal porque hay algunos médicos que no les gusta atender porque no andan de buen humor y a veces no cumplen con el requisito de su vocación o su ética.

ALEXA: Ha escuchado historia de amigas acerca de sus experiencias en los centros de salud?

MUJER: Si, mi mama casualmente ella vino aquí, va a arrancarse una muela porque todavía no se la ha arrancado entonces ella vino a Odontología porque ella paso consulta en la clínica privada pero le dijeron como es más cómodo en el centro de Salud vaya.

ALEXA: Es mas cómodo aquí que en las clínicas privadas?

MUJER: Si porque es un poco más caro en el privado, entonces la doctora le recomendó vaya para que no haga un gasto extra para arrancar la muela porque le costaba cien dólares

ALEXA: Le costaría cien dólares en las clínicas privadas?

MUJER: Si y aquí no, es un poco más cómodo es barato, porque como es para el pueblo entonces es gratuito entonces ella le dijo, trajo la placa y le dijo a la doctora mire que necesito que me extraiga esta muela porque ya me está dando bastante problemas, entonces la doctora le dijo y le contesto de mala manera pero si usted se está atendiendo en una clínica privada vaya usted a una clínica privada, entonces ella por no contestarle porque mi mama quería contestarle, ya que mi mama es una ex trabajadora del estado, fue enfermera más de cuarenta y cinco años ella empezó joven entonces no quiso contestarle porque si uno contesta mal pero lo atienden entonces solo va a esperar que la atienda para decirle que esa no es manera para tratar a un paciente, porque ella viene porque se supone que aquí es gratuito y para eso viene para que la atiendan de buena manera entonces no y es joven la muchacha y no es manera para tratar a una persona de edad porque mi mama ya tiene sesenta y además que ella es ex trabajadora de la salud.

ALEXA: Cree que hay una competencia entre las clínicas privadas y los centros públicos, o como es la relación?

MUJER: Pues la verdad es que yo considero que es el paciente el que escoge de la manera en que quiere ser tratado más profesionalmente porque tal vez ellos siente que son más profesionales en una clínica privada y dado a los recursos también que algunos tienen pero no es que aquí no sepan, porque si saben porque la mayoría que están en una clínica privada pasan por un centro de salud haciendo su pasantía.

Title: Perla-3.docx

Descriptor Info: Age: Interview Location: Residence: Interviewer: Date of Interview:

Codes Applied: Private care

Excerpt Package: 373

Excerpt End: 558

HANNAH: Y usualmente viene a este centro de salud?

MUJER: Si, en los centros de salud porque uno tiene horario para trabajar pero no siempre se tiene para pagar y entonces se viene aquí

Title: SantaAna-4.docx.docx

Descriptor Info: Age: Interview Location: Residence: Interviewer: Date of Interview:

Codes Applied: Private care

Excerpt Package: 802

Excerpt End: 1532

ALEXANDRA: O mejor, alguna vez ha pensado en ir a una clínica privada?

MUJER: Ah, en clínica privada es caro y a veces no tenemos ni para hacernos un examen porque son caros y entonces venimos aquí y nos dan traslado al hospital y es gratis

ALEXANDRA: Y piensa que hay confianza con su información aquí?

MUJER: Si, claro que sí, con mis hijos vengo también y hay mucha confianza

ALEXANDRA: Y nunca ha tenido una mala experiencia con alguien aquí?

MUJER: No, para nada, desde que estaba la Dra. Téllez ella fue una linda persona conmigo porque cuando iba tener a mi hija y ella me dio treinta córdobas para que me fuera al hospital y un amigo se ofreció para irme a dejar hasta allá y no tengo queja de nadie, son lindas personas.

Title: Poneloya-1.docx

Descriptor Info: Age: Interview Location: Residence: Interviewer: Date of Interview:

Codes Applied: Private care

Excerpt Package: 396

Excerpt End: 766

ALEXANDRA: Alguna vez ha pensado en ir a una clínica privada?

MUJER: Si, la verdad es que cuando es algo que pienso no pueden atender me voy a otro lado porque es más rápido

ALEXANDRA: Alguna vez ha tenido una mala experiencia en el centro de salud?

MUJER: No, para nada, ellas son muy buenas en eso, lo único es que hay cosas que es mejor ir directamente a una clínica

Title: Subtiava-1.docx

Descriptor Info: Age: Interview Location: Residence: Interviewer: Date of Interview:

Codes Applied: Private care

Excerpt Package: 15581

Excerpt End: 16690

HANNAH: Y su hermana recibió de una clínica privada el cuido para su enfermedad o de centros de salud?

MUJER: Del centro de Salud.

HANNAH: Y porque decidió el cuidado de un centro de salud y no de una clínica?

MUJER: Primero porque no tiene mucho dinero porque una clínica es muy caro el tratamiento y la consulta entonces ella mejor recurría al centro de salud que es para nosotros los pobres.

HANNAH: Pero usted cree que ella preferiría una clínica privada si ella tuviera él dinero para pagar una clínica o no hay mucha diferencia?

MUJER: No, no hay mucha diferencia pero tal vez si ella veía que el tratamiento no le ayudaba podría ser posible que sí pudo ir a otro médico porque hubiese sido más cómodo pagar un poco y que le dieran tratamiento.

HANNAH: Y el tratamiento de su amiga en Managua afecta mucho por el costo?

MUJER: Si es muy caro, el transporte, solo el transporte es caro más ahora la clínica.

HANNAH: Ella está viviendo en León y viaja a Managua para sus tratamientos?

MUJER: Vive en Poneloya.

HANNAH: Oh es difícil.

MUJER: Es muy caro solo el viaje ahora más la clínica, los tratamientos.

Title: Subtiava-1.docx

Descriptor Info: Age: Interview Location: Residence: Interviewer: Date of Interview:

Codes Applied: Private care

Excerpt Package: 12577

Excerpt End: 13188

HANNAH: Y porque piensa que las clínicas privadas son mejores para el cuidado del cáncer?

MUJER: Ella para tener mejor seguridad, mejores médicos según ellos mejores especialistas para tratar, mejor cuidado y eso es lo que yo pienso pero claro yo no podría hacer eso porque no tengo con costo lo que gano para mí, yo por eso busco lugares como este e incluso yo no busco médicos sino es necesario que por una tos, yo me hago un cocimiento compro pastillas y busco yo misma pero si miro que ya no puedo entonces yo busco el medico porque ya vi que ya no puedo yo sola entonces recurro al médico como última hora.

Title: Perla-2.docx

Descriptor Info: Age: Interview Location: Residence: Interviewer: Date of Interview:

Codes Applied: Private care

Excerpt Package: 10

Excerpt End: 1692

ALEXANDRA: Bueno, entonces para empezar puedes hablarme un poco sobre el cuidado de salud en general?

MUJER: Aquí como lo tratan a uno? Pues la verdad mal porque primeramente yo estaba con la enfermera porque primeramente me mandan una cita y me dicen que la venga a agarrar aquí, después me dijeron que tenía que venir personalmente la muchacha porque es para mi hija y les digo de que ella está trabajando que no puede venir y me dicen que tiene que venir ella sino no me dan la cita, vengo y hablo con el Licenciado, el me dio una orden para que ella me atendiera pero ella me dijo muchas cosas, diciendo que no me podía atender por que a ella no le pertenecía esa área ya le dije que es allá, si le dije pero yo solo quería información entonces me mandaron al otro lugar y me atendieron ahora que vengo a la cita me dijeron que tenía que traer la cedula entonces le dije yo que en el papel venia la cedula, no me dice es que es un requisito nuestro que deben traer cedula, entonces yo le dije que estábamos haciendo la gran cola de la fila y volver a ir a casa y regresar otra vez a hacer de nuevo fila, no era justo y le dije que por favor me atendiera entonces me dijo de que no podía, así que fui donde el médico y el me dio un papelito y me dijo que si, que me podían atender entonces dijo ella, les voy a hacer el favor pero yo no puedo hacer esto para en otra traigan su cedula, eso es todo lo que nos debes de decir dije yo, tranquila, entonces tuve ese inconveniente con ella.

ALEXANDRA: Y cree que su experiencia podría ser mejor si va a otro lugar o a una clínica privada?

MUJER: Si en una clínica privada es mejor pero como uno no tiene el dinero tiene que aguantarse

Title: Mantica-5.docx

Descriptor Info: Age: Interview Location: Residence: Interviewer: Date of Interview:

Codes Applied: Private care

Excerpt Package: 3627

Excerpt End: 4173

HANNAH: Piensa que el cáncer de cuello uterino es algo muy común que puede afectarle en un futuro o es algo muy raro?

MUJER: Yo digo que es algo muy grave porque no nos cuidamos, digo no nos cuidamos porque yo no estoy pendiente de mis exámenes, a veces digo hoy no me lo hare será hasta el otro mes y ahí no me cuido.

HANNAH: Después de lo de su amiga piensa que le afecta e influye a que se haga ms pruebas de paps?

MUJER: Ah sí, ahora lo más que tardo son ocho meses en hacerme el examen y me hago uno aquí en el Centro de Salud y otro privado

Title: Santa Ana-2.docx

Descriptor Info: Age: Interview Location: Residence: Interviewer: Date of Interview:

Codes Applied: Private care

Excerpt Package: 909

Excerpt End: 1118

ALEXANDRA: Y alguna vez ha pensado en visitar una clínica privada?

MUJER: Claro que si, por lo menos a mí me gustaría pero somos de escasos recursos y uno no tiene para las clínicas privadas y están son caras.

**Private/taboo**

Title: Los Leches-3.docx

Descriptor Info: Age: Interview Location: Residence: Interviewer: Date of Interview:

Codes Applied: Private/taboo

Excerpt Package: 4870

Excerpt End: 5187

SARA: Con quien se sentiría cómodo hablando de cáncer de cuello uterino u otras enfermedades como papiloma, con su familia con su hija, con amigos?

MUJER: Tal vez con mi mama porque mi hija no me va atender porque esta pequeña

SARA: Pero en un futuro hablara de estas enfermedades con su hija?

MUJER: Sí, claro que si

Title: Los Leches-3.docx

Descriptor Info: Age: Interview Location: Residence: Interviewer: Date of Interview:

Codes Applied: Private/taboo

Excerpt Package: 3708

Excerpt End: 3944

SARA: Y como es el conocimiento y actitud en general de la comunidad acerca del virus de papiloma humano hablan mucho sobre esto o es un tabú?

MUJER: La verdad es que lo oigo más de la televisión porque a la gente casi no se le escucha

Title: Mantica-5.docx

Descriptor Info: Age: Interview Location: Residence: Interviewer: Date of Interview:

Codes Applied: Private/taboo

Excerpt Package: 3475

Excerpt End: 3626

HANNAH: Y después de su muerte hay personas en la comunidad que hablan de cáncer de cuello uterino o nadie habla de eso??

MUJER: No, nadie habla de eso

Title: Santa Ana-1.docx

Descriptor Info: Age: Interview Location: Residence: Interviewer: Date of Interview:

Codes Applied: Private/taboo

Excerpt Package: 5209

Excerpt End: 5490

SARA: Con quien se sentiría cómoda de hablar de estas enfermedades?

MUJER: La verdad con los médicos

SARA: Y porque no con su familia?

MUJER: No se

SARA: Y con su marido

MUJER: Si

SARA: Y va a hablar con sus hijos e hijas cuando estén más grandes acerca de esto?

MUJER: Creo que si

Title: Perla-4.docx

Descriptor Info: Age: Interview Location: Residence: Interviewer: Date of Interview:

Codes Applied: Private/taboo

Excerpt Package: 1057

Excerpt End: 1280

HANNAH: Y se sentía cómoda hablando de ese virus con otras personas? Si puedes hablar con otras personas o es algo muy privado para usted?

MUJER: No, yo pienso que no lo hablo normal porque hay otras personas que lo padecen

Title: Perla-5.docx

Descriptor Info: Age: Interview Location: Residence: Interviewer: Date of Interview:

Codes Applied: Private/taboo

Excerpt Package: 3925

Excerpt End: 5229

ALEXA: Usted piensa que las jóvenes reciben suficiente información?

MUJER: Todavía hay tabú sobre eso, bastante tabú, usted sabe que aquí en Nicaragua todavía es una sociedad que es bastante tradicional, bastante cultural, entonces hay bastante tabú sobre eso todavía, sobre la información acerca de la sexualidad tal vez en lugares más rurales, como en comarcas tal vez ellos son más cerrados, hay bastante machismo entonces se considera que aunque estamos en un siglo XXI es bastante cerrado considero yo sobre eso, porque ahorita se ha aumentado la tasa de mortalidad sobre las mujeres, se escucha en las noticias entonces tal vez se necesita bastante educación porque es eso tanto en la casa que es donde uno comienza, entonces ahí ya se va pasando de generación en generación de que las mujeres tienen que atender al esposo, el marido es todo, entonces así vamos y ya el niño y la niña también vienen adoptando esa postura de que no somos iguales, de que él o ella puede hacer una cosa mientras el otro hace otra, que si yo estoy enferma un día, talvez tú me atiendes, algo así reciproco, entonces falta esa educación, yo creo que todavía nos falta como un cincuenta por ciento porque a como le digo hay bastante tabú nuestra sociedad es bastante tradicional y también la tecnología afecta bastante.

Title: Perla-5.docx

Descriptor Info: Age: Interview Location: Residence: Interviewer: Date of Interview:

Codes Applied: Private/taboo

Excerpt Package: 9307

Excerpt End: 9745

ALEXA: Y con quien se sentiría cómoda hablar del cáncer de cuello uterino?

MUJER: Pues a como le digo yo no soy tan cerrada, creo que los varones pueden hacer una buena charla no solo las mujeres, es solo que a veces las mujeres tienen más confianza con las mismas mujeres porque aquí el de ginecología es un doctor, no hay mujeres sino que es un doctor y entonces el papel del doctor tienen que pasar con él aunque no se sientan cómodas.

Title: Mantica-6.docx

Descriptor Info: Age: Interview Location: Residence: Interviewer: Date of Interview:

Codes Applied: Private/taboo

Excerpt Package: 5352

Excerpt End: 5883

ALEXANDRA: Y ha oído del virus de papiloma humano antes de hoy?

MUJER: cercano a mí a mis alrededores con mis familiares no, pero si por medio del internet y personas que se han relacionado con personas que se ven demasiado tarde lo he escuchado y lo he seguido demasiado cerca para saber más sobre los resultados y que te da, es donde te da temor y por eso yo asiste con más frecuencia a los centros de salud por eso es que hay que sacarlo del tabú y hablarlo más para que uno no se alarme para que se sepa de que hay que cuidarse

Title: Los Leches-2.docx

Descriptor Info: Age: Interview Location: Residence: Interviewer: Date of Interview:

Codes Applied: Private/taboo

Excerpt Package: 2766

Excerpt End: 3271

HANNAH: Como piensa es el conocimiento y actitud acerca de este virus? Las personas hablan sobre este virus de papiloma humano o es una cosa muy privada?

MUJER: Hay personas que no les gusta hablar de eso, como yo nunca he tenido eso no le podría decir mucho al respecto

HANNAH: Y porque piensa es una cosa privada en su comunidad?

MUJER: Porque si alguien se le detecta eso no lo va a andar hablando conmigo o cualquiera se lo reserva para sí mismo me imagino o solo que se le diga a alguien de confianza

Title: Mantica-2.docx

Descriptor Info: Age: Interview Location: Residence: Interviewer: Date of Interview:

Codes Applied: Private/taboo

Excerpt Package: 3515

Excerpt End: 3675

ALEXANDRA: Se sentiría cómoda de hablar del cáncer cervical o algo como eso con un médico varón

MUJER: No tanto, sería mejor con una doctora, familia y mi madre

Title: Subtiava-2.docx

Descriptor Info: Age: Interview Location: Residence: Interviewer: Date of Interview:

Codes Applied: Private/taboo

Excerpt Package: 5677

Excerpt End: 6116

HANNAH: Piensa que hay muchas personas que hablan de cáncer de cuello uterino en la ciudad?

MUJER: Bueno yo creo que es más que todo en los centros de salud.

HANNAH: Piensa que hay una forma de discriminación a las personas con virus de papiloma humano o cáncer?

MUJER: Bueno, no sé, lo que yo sé es que una enfermedad que se puede tratar haciéndose los chequeos y pruebas además que se puede evitar, pero discriminación en sí, no, no creo

Title: Subtiava-2.docx

Descriptor Info: Age: Interview Location: Residence: Interviewer: Date of Interview:

Codes Applied: Private/taboo

Excerpt Package: 8292

Excerpt End: 8439

HANNAH: Con quien se sentiría cómoda hablando del vph y cáncer de cuello uterino?

MUJER: Pues es un tema que puedo platicar con cualquier persona.

Title: Santa Ana-1.docx

Descriptor Info: Age: Interview Location: Residence: Interviewer: Date of Interview:

Codes Applied: Private/taboo

Excerpt Package: 3956

Excerpt End: 4123

SARA: Bueno, y como es la actitud y conocimiento en general de la comunidad acerca de esta enfermedad hablan mucho sobre esto o es un tabú?

MUJER: Si se habla bastante

Title: Perla-5.docx

Descriptor Info: Age: Interview Location: Residence: Interviewer: Date of Interview:

Codes Applied: Private/taboo

Excerpt Package: 12028

Excerpt End: 12849

ALEXA: Solo nos faltan unas preguntitas acerca del papiloma, como piensa que es la actitud de la comunidad acerca de esta enfermedad?

MUJER: Bueno eso le sucede más a los adolescentes bastante pero a como le digo es porque hay varones que andan con varias mujeres y talvez le transmiten esa enfermedad a su esposa o compañera y no le gusta que vaya a un centro de salud a tratar la enfermedad porque son personas machistas pero le estoy hablando de lugares talvez todavía del campo o comarcas y que todavía se dan esas situaciones porque es bastante tabú y aquí en la ciudad se da en los adolescentes que comienzan a tener relaciones sexuales y tal vez por pena no quieren ir a los centros de salud tal vez las muchachas y también los varones porque para ellos no es común es más común ver a las mujeres ir a ginecología.

Title: Santa Ana-3.docx

Descriptor Info: Age: Interview Location: Residence: Interviewer: Date of Interview:

Codes Applied: Private/taboo

Excerpt Package: 3666

Excerpt End: 4958

HANNAH: Después de su muerte hablan más acerca de esta enfermedad con su familia? Como le afecta esto a su familia?

MUJER: Bueno, nos afecta mucho porque nunca pensamos que alguna de esas enfermedades existía hasta que ya nos pasó y sufrimos esa experiencia fue que supimos que si era cierto lo de esa enfermedad del cáncer de cuello uterino

HANNAH: Durante el tratamiento de su tía piensa de que ella hablo con miembros de su familia o amigos para apoyo?

MUJER: Bueno, nosotros la familia nos dimos cuenta cinco días antes de que ella muriera

HANNAH: Disculpe, ustedes se dieron cuenta hasta cinco días antes?

MUJER: Si, cinco días antes de fallecer supimos que era cáncer

HANNAH: Porque ella no quería hablar?

MUJER: Si, no quería hablar, la única que sabía era mi mama

HANNAH: Usted piensa que ella estaba muy preocupada acerca de esta enfermedad? Piensa que es algo común?

MUJER: Pensaban que era una enfermedad común que no le tomaban importancia

HANNAH: Pero piensa que es común de no hablar de la enfermedad?

MUJER: Bueno, por ejemplo que hizo mi tía de no hablar fue muy malo porque somos inocentes de lo que ella estaba sufriendo por eso es que es bueno hablar e irse a revisar yo digo que deben revisarse y hacerse el pap para detectar cualquier enfermedad o infección a tiempo.

Title: Los Leches-1 (1).docx

Descriptor Info: Age: Interview Location: Residence: Interviewer: Date of Interview:

Codes Applied: Private/taboo

Excerpt Package: 5459

Excerpt End: 5747

Sara: I mean if is acceptable to talk about sexual diseases, with friends, neighbors or family?

Woman: I do not know, I do not like to talk about it with others.

Sara: Why?

Woman: I do not know, I have never liked to talk about it, I always thought that is something that should be quiet.

Title: Mantica-5.docx

Descriptor Info: Age: Interview Location: Residence: Interviewer: Date of Interview:

Codes Applied: Private/taboo

Excerpt Package: 6004

Excerpt End: 6144

ANNAH: Piensa usted que en cualquier situación se sentiría cómoda hablando con su pareja acerca del VPH y cáncer de cuello uterino?

MUJER: S

Title: Mantica-1.docx

Descriptor Info: Age: Interview Location: Residence: Interviewer: Date of Interview:

Codes Applied: Private/taboo

Excerpt Package: 7330

Excerpt End: 7720

Sara: Con quien se sentiría cómoda de hablar de cáncer de cuello uterino?

Mujer: Con cualquier persona, con tal que sepa sobre la enfermedad y me enseñe las cosas de las charlas con cualquiera me da igual

Sara: Y va a hablar con sus hijos cuando sean grandes acerca de estas enfermedades, porque?

Mujer: Si porque uno sufre, cae en cama y lo van matando poco a poco, todas esas enfermedades

Title: Santa Ana-3.docx

Descriptor Info: Age: Interview Location: Residence: Interviewer: Date of Interview:

Codes Applied: Private/taboo

Excerpt Package: 5548

Excerpt End: 6128

HANNAH: Y cómo piensa usted que es la actitud y conocimiento de las mujeres en la comunidad acerca de esta enfermedad?

MUJER: Bueno hoy en día veo que son más dóciles porque ya se están yendo a hacer más las revisiones sobre el cáncer de mama, uterino y otras enfermedades más, ahora es más abierto porque ponen unos rótulos y eso es muy bueno porque así leen y así ellas están prestando atención cuando están las charlas o cuando están en consulta la doctora les pregunto o ellas a la doctora y eso es lo bueno, aunque ellas no anden eso pero la doctora siempre está insistiendo.

Title: SantaAna-4.docx.docx

Descriptor Info: Age: Interview Location: Residence: Interviewer: Date of Interview:

Codes Applied: Private/taboo

Excerpt Package: 7078

Excerpt End: 7446

ALEXANDRA: Si usted contrae el cáncer de cuello uterino por ejemplo con quien se sentiría cómoda hablar acerca de su enfermedad?

MUJER: Bueno yo hablaría y tendría que someterme a la cosa de Dios porque solamente él puede curar de cualquier enfermedad

ALEXANDRA: Entonces le informaría algo a sus hijos?

MUJER: Si se lo diría a mis hijos, a mi marido y a mi familia

Title: Poneloya-2.docx

Descriptor Info: Age: Interview Location: Residence: Interviewer: Date of Interview:

Codes Applied: Private/taboo

Excerpt Package: 11663

Excerpt End: 11907

ALEXA: Y ella le dijo a sus hijos que tenía cáncer?

MUJER: Si, ella le dijo a sus niños y a su esposo, ya ahora sus niños son adolescentes y ella ahí está pero ella ya sabe lo que tiene y yo se lo decía a ella que tuviera cuidado por eso mismo.

Title: Mantica-5.docx

Descriptor Info: Age: Interview Location: Residence: Interviewer: Date of Interview:

Codes Applied: Private/taboo

Excerpt Package: 1940

Excerpt End: 2903

HANNAH: usted ha tenido una experiencia con el cáncer de cuello uterino, alguien de su familia o algún amigo que lo haya sufrido?

MUJER: Un conocido, en mi familia Gracias a Dios no, nadie lo ha tenido

HANNAH: Conocido en su familia

MUJER: No, era alguien del Reparto

HANNAH: Ha oído las experiencias de su amiga que ha sufrido de cáncer

MUJER: SI

HANNAH: Como fue su experiencia?

MUJER: Pues fue muy triste porque ella murió a causa de eso, ya que era muy tarde cuando se lo descubrieron, en la Iglesia también a una muchacha le quitaron un pecho y le sacaron y Gracias a Dios ella sobrevivió, pero la que estaba más cerca de mi casa, murió.

HANNAH: Pero ella estaba cómoda hablando acerca de su tratamiento?

MUJER: No porque cuando a ella se lo descubrieron ya no había nada que hacer, todo fue muy tarde.

HANNAH: Y porque piensa que es una cosa muy privada?

MUJER: No sé, pero yo digo que tenemos que hablar para que así estos temas no afecten a otras mujeres

Title: Santa Ana-2.docx

Descriptor Info: Age: Interview Location: Residence: Interviewer: Date of Interview:

Codes Applied: Private/taboo

Excerpt Package: 5604

Excerpt End: 5791

ALEXANDRA: Si se habla mucho de la enfermedad o si es un tema más tabú?

MUJER: Que si se habla mucho de la enfermedad del cáncer? Si se habla por todos lados de esta enfermedad del cáncer

Title: Subtiava-1.docx

Descriptor Info: Age: Interview Location: Residence: Interviewer: Date of Interview:

Codes Applied: Private/taboo

Excerpt Package: 13189

Excerpt End: 13576

HANNAH: Durante la experiencia de su hermana con cáncer de cuello uterino ella hablo con otras personas acerca de su experiencia?

MUJER: No casi no, con nadie solamente con mi mama y conmigo.

HANNAH: Y porque cree que ella no quería hablar con otras personas?

MUJER: Porque le da pena y además que allá en el dispensario de Poneloya las doctoras hablan muy público y todo mundo escucha.

Title: Perla-2.docx

Descriptor Info: Age: Interview Location: Residence: Interviewer: Date of Interview:

Codes Applied: Private/taboo

Excerpt Package: 3644

Excerpt End: 4429

ALEXANDRA: Y cual piensa usted que es la actitud de la comunidad acerca del cáncer de cuello uterino?

MUJER: No sé, nosotros no queremos que se den cuenta porque comienzan a criticar a uno

ALEXANDRA: Critican a las mujeres que tienen cáncer?

MUJER: Si

ALEXANDRA: Entonces, como es la experiencia para una mujer que tiene el cáncer de cuello uterino?

MUJER: Uhm es como vivirlo solo

ALEXANDRA: Y con quien se sentiría cómoda de hablar de cáncer de cuello uterino? Por ejemplo si usted lo contrajera?

MUJER: Con mi hermana, porque mi mama y papa ya murieron y mi hermana es la más cercana a mi

ALEXANDRA: Y su hija o su pareja?

MUJER: No

ALEXANDRA: Como es el conocimiento de la comunidad sobre esta enfermedad?

MUJER: Yo digo que saben pero como siempre uno siempre se queda callado

Title: Perla-4.docx

Descriptor Info: Age: Interview Location: Residence: Interviewer: Date of Interview:

Codes Applied: Private/taboo

Excerpt Package: 4798

Excerpt End: 5436

HANNAH: Pero ella murió a causa de su cáncer?

MUJER: Si

HANNAH: Y ella hablo mucho con usted acerca de su enfermedad?

MUJER: Sí, es que ella era casi vecina como a dos cuadras

HANNAH: Y piensa que ella hablo con muchas personas en su vida acerca del cáncer?

MUJER: Yo diría que sí, porque ella era muy ágil y no decayó por mucho tiempo.

HANNAH: Y piensa usted que usualmente las personas con cáncer se sentirían cómodas hablando de su enfermedad con los demás o depende de las personas?

MUJER: Uhm, ella que si hablaba de esa manera? Pues si yo creo que si lo acepto con terapias que les dan en algunos lugares para aceptar la enfermedad

Title: Mantica-6.docx

Descriptor Info: Age: Interview Location: Residence: Interviewer: Date of Interview:

Codes Applied: Private/taboo

Excerpt Package: 4683

Excerpt End: 4960

ALEXANDRA: Cree que hay un estigma o un tabú con respecto al cáncer de cuello uterino?

MUJER: Hoy en día creo que ya no, la tecnología está avanzada y uno se informa con frecuencia rápidamente si sea en lo rural o urbano yo creo que estamos muy pendiente sobre todo las mujeres

Title: Perla-3.docx

Descriptor Info: Age: Interview Location: Residence: Interviewer: Date of Interview:

Codes Applied: Private/taboo Fear

Excerpt Package: 11039

Excerpt End: 12603

HANNAH: Y con quien hablo acerca de su enfermedad? Es decir ella hablo mucho con su familia o con amigos?

MUJER: No, solo con nosotros sus hijas

HANNAH: Solo sus hijas?

MUJER: Si solamente sus hijas con nadie más, después de eso entonces se sintió en el deber y derecho de comentárselo a una hermana de ella que trabajaba en el hospital.

HANNAH: Y por qué piensa usted que es una cosa muy privada para su madre?

MUJER: Sabe porque, porque muchas personas lo toman como que cuando una mujer tiene esa enfermedad dicen que es por andar de p.. Y dicen un sin número de cosas, y es una discriminación fea por eso es que muchas mujeres cuando tienen esa enfermedad mejor se quedan calladas y no dicen nada.

HANNAH: Que es una enfermedad fea?

MUJER: Si una enfermedad fea y además de que es fea es una enfermedad de la cual las personas la toman para discriminar a los que la padecen, para hablar de la persona que tiene ese problema, pero la realidad es que ese problema todas lo podemos adquirir porque por más que una mujer este en una casa protegiéndose y cuidándose, no sabe si el marido anda con una y otra en las calles entonces por eso muchas se quedan calladas porque empiezan a discriminar pero si hay otras personas que tienen la misma enfermedad y se sienten en confianza de platicar conmigo y yo digo que pobres porque la verdad es que ellas no desean tener ese problema, porque una muchacha que también lo tiene me dijo que ella lo tiene en la sangre es decir en etapa A y que el médico le dijo que le pueden dar el tratamiento para ver si este evoluciona.

Title: Perla-2.docx

Descriptor Info: Age: Interview Location: Residence: Interviewer: Date of Interview:

Codes Applied: Private/taboo Fear

Excerpt Package: 8020

Excerpt End: 8215

ALEXANDRA: Muchas gracias, disculpa tengo una pregunta más que ella me sugirió, porque cree que las personas les esconden las cosas a sus hijos más sobre estos temas?

MUJER: Es para no asustarlos

Title: Mantica-3.docx

Descriptor Info: Age: Interview Location: Residence: Interviewer: Date of Interview:

Codes Applied: Private/taboo HPV Cervical cancer

Excerpt Package: 6940

Excerpt End: 7494

HANNAH: Usted es de una región urbana o rural es decir más del campo

MUJER: Pues rural, ms del campo creo yo

HANNAH: Piensa que hay menos información o se habla menos de estas enfermedades en su comunidad?

MUJER: Pues se habla mucho, porque a pesar que vivamos en el campo ahora se sabe más de estas enfermedades de las mujeres porque hoy en día se puede ver hasta en la televisión sobre las enfermedades y eso porque ahora a como esta tan actualizado nuestro mundo entonces nos damos cuenta de todo, antes no sabíamos nada ahora si estamos actualizados.

Title: Perla-3.docx

Descriptor Info: Age: Interview Location: Residence: Interviewer: Date of Interview:

Codes Applied: Private/taboo Promiscuity

Excerpt Package: 11039

Excerpt End: 12603

HANNAH: Y con quien hablo acerca de su enfermedad? Es decir ella hablo mucho con su familia o con amigos?

MUJER: No, solo con nosotros sus hijas

HANNAH: Solo sus hijas?

MUJER: Si solamente sus hijas con nadie más, después de eso entonces se sintió en el deber y derecho de comentárselo a una hermana de ella que trabajaba en el hospital.

HANNAH: Y por qué piensa usted que es una cosa muy privada para su madre?

MUJER: Sabe porque, porque muchas personas lo toman como que cuando una mujer tiene esa enfermedad dicen que es por andar de p.. Y dicen un sin número de cosas, y es una discriminación fea por eso es que muchas mujeres cuando tienen esa enfermedad mejor se quedan calladas y no dicen nada.

HANNAH: Que es una enfermedad fea?

MUJER: Si una enfermedad fea y además de que es fea es una enfermedad de la cual las personas la toman para discriminar a los que la padecen, para hablar de la persona que tiene ese problema, pero la realidad es que ese problema todas lo podemos adquirir porque por más que una mujer este en una casa protegiéndose y cuidándose, no sabe si el marido anda con una y otra en las calles entonces por eso muchas se quedan calladas porque empiezan a discriminar pero si hay otras personas que tienen la misma enfermedad y se sienten en confianza de platicar conmigo y yo digo que pobres porque la verdad es que ellas no desean tener ese problema, porque una muchacha que también lo tiene me dijo que ella lo tiene en la sangre es decir en etapa A y que el médico le dijo que le pueden dar el tratamiento para ver si este evoluciona.

Title: Mantica-4.docx

Descriptor Info: Age: Interview Location: Residence: Interviewer: Date of Interview:

Codes Applied: Private/taboo Sexual education

Excerpt Package: 4793

Excerpt End: 5071

HANNAH: Piensa que en un futuro va a hablar con sus hijos sobre su experiencia y sobre la salud sexual?

MUJER: Si, por lo menos para que ellos sepan lo que en el futuro les espera y así tener confianza con los hijos y poder platicar abiertamente con ellos para que sepan de todo

Title: Perla-3.docx

Descriptor Info: Age: Interview Location: Residence: Interviewer: Date of Interview:

Codes Applied: Private/taboo Support

Excerpt Package: 9158

Excerpt End: 10114

MUJER: Pues realmente le voy a decir cuando nosotros nos separamos de ella porque cada quien decidió hacer su vida, ella quedo sola en la casa pero ella muchas veces reservo muchas cosas que nosotros no nos comentaba, pero al verse en ese caso nos comentó a nosotros de lo que pasaba, y yo en una plática en confianza le pregunte a ella sobre eso, porque ella sabe lo que se siente lo que se vive y yo no sé entonces así para ser orientada y darme cuenta cuales son los síntomas que es lo que se siente entonces le pregunté, tome el abuso de preguntarle me dio pena pero ni modo fue una plática que se dio y tenía que darse, entonces yo le pregunte a ella y me dijo que a ella lo que le daba era humedad, ardor y picazón al comienzo y ella se trataba para eso, pero después miro que era mucho que no tenía solución entonces tomo la decisión de pasar consulta y pedir cita con el ginecólogo y ahí fue donde comenzó a darle seguimiento y eso fue lo que paso.

Title: Poneloya-1.docx

Descriptor Info: Age: Interview Location: Residence: Interviewer: Date of Interview:

Codes Applied: Private/taboo Support

Excerpt Package: 2531

Excerpt End: 3117

ALEXANDRA: Wow y el papiloma es muy común aquí en la comunidad?

MUJER: Pues que yo sepa no, es que creo que eso es algo reservado entre el doctor y la paciente.

ALEXANDRA: Oh si, entonces la gente no habla sobre ese tema?

MUJER: NO

ALEXANDRA: Con quien se sentiría cómoda hablando del virus de papiloma humano?

MUJER: Como? Si yo lo tuviera? Creo que en ese caso, lo primero ir a un centro de salud o la clínica y ellos le informaran lo que tienen y pues hablar con el médico.

ALEXANDRA: También con su familia?

MUJER: Si, puede ser pero yo ahorita puedo decir que si en ese momento no

Title: Santa Ana-2.docx

Descriptor Info: Age: Interview Location: Residence: Interviewer: Date of Interview:

Codes Applied: Private/taboo Support

Excerpt Package: 6922

Excerpt End: 7043

ALEXANDRA: Y ella le dijo a sus hijos y su pareja que tenia el cáncer de cuello uterino?

MUJER: Si todos se dieron cuenta

Title: Los Leches-2.docx

Descriptor Info: Age: Interview Location: Residence: Interviewer: Date of Interview:

Codes Applied: Private/taboo Support

Excerpt Package: 3933

Excerpt End: 4289

HANNAH: Piensa que las personas están cómodas hablando con sus amigos y familia acerca del cáncer?

MUJER: Con la familia tal vez sí, pero eso no se le puede decir a cualquiera, porque siendo así mejor se va a un centro de salud para saber que dice un medico

HANNAH: Así que usted prefiere hablar de estos temas con la familia y doctores?

MUJER: Si, así es

Title: Mantica-4.docx

Descriptor Info: Age: Interview Location: Residence: Interviewer: Date of Interview:

Codes Applied: Private/taboo Support

Excerpt Package: 2060

Excerpt End: 3085

HANNAH: Y durante este proceso se sentía cómoda hablando de su enfermedad con su familia, amigos o es algo más privado para usted?

MUJER: Si es algo más privado

HANNAH: Con quien se sentiría más cómoda hablando sobre el cáncer?

MUJER: Disculpe no le entendí bien

HANNAH: Bueno, me refiero que con quien puede hablar sobre su enfermedad?

MUJER: Bueno, solamente con mi medico

HANNAH: Tiene una pareja?

MUJER: Bueno, si, a él también tengo que decirle porque no puedo ocultarle anda de esto

HANNAH: Pero su pareja sabe de su enfermedad que ha tenido cáncer y su tratamiento?

MUJER: Si, si lo sabe

HANNAH: Pero prefiere hablar con un médico o una enfermera sobre su enfermedad más que con otros?

MUJER: Si

HANNAH: Y sus padres saben también?

MUJER: Bueno mi papa que es el único que está vivo, si está al tanto de mis problemas

HANNAH: Y puede hablar de eso con sus amigos o ellos no saben?

MUJER: No, solamente primero Dios que es el único que nos protege, mi médico que lo descubrió, mi pareja y mi papa saben de mis problemas

Title: Perla-3.docx

Descriptor Info: Age: Interview Location: Residence: Interviewer: Date of Interview:

Codes Applied: Private/taboo Support

Excerpt Package: 6600

Excerpt End: 7276

HANNAH: Muerte por el virus o de otra cosa?

MUJER: No, de eso por el virus porque yo por ejemplo yo tengo una tía y mi tía tiene eso y ella no se recuperaba, esta delgada porque eso te enferma psicológicamente también.

HANNAH: Usted hablo un poquito de su tía, no sé si recuerda.

MUJER: Si, es decir ella no vive conmigo pero vive cerca de mi casa, pero si ella padece eso y ella es promiscua y entonces que le resulto, que le haya pasado eso porque no se cuida.

HANNAH: Y habla con usted acerca de su virus o es una cosa privada que ella no le gusta hablar?

MUJER: Uhm, pues no, no le gusta hablar pero parece que ella se lo comento a otra tía mía y ella nos dijo a nosotros.

Title: Perla-3.docx

Descriptor Info: Age: Interview Location: Residence: Interviewer: Date of Interview:

Codes Applied: Private/taboo Support

Excerpt Package: 9118

Excerpt End: 10114

HANNAH: Y como encontró ella su cáncer?

MUJER: Pues realmente le voy a decir cuando nosotros nos separamos de ella porque cada quien decidió hacer su vida, ella quedo sola en la casa pero ella muchas veces reservo muchas cosas que nosotros no nos comentaba, pero al verse en ese caso nos comentó a nosotros de lo que pasaba, y yo en una plática en confianza le pregunte a ella sobre eso, porque ella sabe lo que se siente lo que se vive y yo no sé entonces así para ser orientada y darme cuenta cuales son los síntomas que es lo que se siente entonces le pregunté, tome el abuso de preguntarle me dio pena pero ni modo fue una plática que se dio y tenía que darse, entonces yo le pregunte a ella y me dijo que a ella lo que le daba era humedad, ardor y picazón al comienzo y ella se trataba para eso, pero después miro que era mucho que no tenía solución entonces tomo la decisión de pasar consulta y pedir cita con el ginecólogo y ahí fue donde comenzó a darle seguimiento y eso fue lo que paso.

Title: Mantica-3.docx

Descriptor Info: Age: Interview Location: Residence: Interviewer: Date of Interview:

Codes Applied: Private/taboo Support

Excerpt Package: 1563

Excerpt End: 1900

HANNAH: Porque cree usted que su madre no quiso sacarse la matriz?

MUJER: No sé porque no quiso, no le sabría decir realmente solo nos dimos cuenta cuando la llevaron al hospital Lenin Fonseca la operaron y le diagnosticaron el cáncer terminal y nos dijeron que no le daban ni meses ni años de vida, en cualquier momento ella podía morir

Title: Los Leches-1 (1).docx

Descriptor Info: Age: Interview Location: Residence: Interviewer: Date of Interview:

Codes Applied: Private/taboo Support

Excerpt Package: 2857

Excerpt End: 3456

Sara: What type of disease said you have at first?

Woman: I did it in Mantica and they told me I had malignant cells and then took me out the womb, after that I start to be healthy.

Sara: What kind of treatment did they use with you? Was like radiotherapy?

Woman: No. Was something like in the vagina.

Sara: Have you talked with your family about this treatment and problem?

Woman: Sometimes, but little is just that I am ashamed, sometimes I talk to them about it and what happened to me and say it was a grace from God because they said I had it and after I was healthy. This was twenty years ago.

Title: Mantica-3.docx

Descriptor Info: Age: Interview Location: Residence: Interviewer: Date of Interview:

Codes Applied: Private/taboo Support

Excerpt Package: 2476

Excerpt End: 3379

HANNAH: Piensa que el cáncer del cuello uterino y el vph o virus de papiloma humano es algo que se puede hablar con sus amigos y familiares o es algo más privado?

MUJER: Yo digo que es algo más privado, no le sabría decir, por lo menos yo ahorita estoy haciéndome exámenes colposcopia y todo lo que mandan a hacer para ver si es bueno o malo si hay células malas, la verdad es que yo no entiendo mucho solo los médicos son los que saben y yo acato lo que ellos me indican hacer

HANNAH: Cuando su madre estaba recibiendo tratamiento ella hablo mucho sobre su enfermedad con su familia y amigos?

MUJER: Fíjese que no, nosotros nos dimos cuenta cuando a ella la operaron hace siete años en el hospital Lenin Fonseca, hasta ese momento supimos porque ella nunca nos dijo nada siempre se lo quedo callada, la operaron por otras razones y ahí le diagnosticaron el cáncer terminal y que no había nada que hacer

**Promiscuity**

Title: Perla-3.docx

Descriptor Info: Age: Interview Location: Residence: Interviewer: Date of Interview:

Codes Applied: Promiscuity

Excerpt Package: 7603

Excerpt End: 8044

HANNAH: Y ha tenido una experiencia con el cáncer de cuello uterino, sabe de una persona que ha sufrido de cáncer?

MUJER: Mi mama.

HANNAH: Su madre? Oh lo siento mucho

MUJER: Si a ella le hicieron una cono biopsia es en el cuello, y después de eso le dieron tratamiento para ver qué pasaba y ella era una ama de casa que no salía a ningún lado pero resulta que mi Papa fue el de la culpa, como el salía y le gustaba andar con muchas mujeres.

Title: Subtiava-1.docx

Descriptor Info: Age: Interview Location: Residence: Interviewer: Date of Interview:

Codes Applied: Promiscuity

Excerpt Package: 3173

Excerpt End: 4591

HANNAH: Como piensa usted que puede mejorar la educación de la salud sexual?

MUJER: Bueno, cuidarse, protegerse, porque si hay problemas no de parte de la mujer sino del varón tienen que cuidarse y protegerse porque si no después salen afectando a la mujer de su casa porque eso es un riesgo, así me paso una vez no a mi sino que a él y entonces yo le dije, mira que te paso y entonces dijo ve donde la doctora por mí, yo le dije yo no voy a ir, vas a ir tu porque tú eres el del problema , entonces como te paso eso, era porque el andaba con otras mujeres vagas.

HANNAH: Porque el hombre estaba con otras mujeres?

MUJER: Si, así es.

HANNAH: Es su amigo o su amiga?

MUJER: Amiga y entonces le digo yo entonces yo voy a ir al médico, nunca he ido a una clínica a chequearme y me dice la doctora no vaya a pelear pero usted está bien sana y si de usted hubiera sido el problema, porque él me acusaba a mí que yo le había pasado esa enfermedad entonces la doctora me dice si usted se lo hubiera pasado es porque usted hubiera tenido relaciones con otro hombre pero en este caso no es usted es el, entonces yo llegue enojada y le pegue y el riéndose después me dijo quien había sido la persona y yo le dije anda donde la doctora y dijo que no iba a ir pero como los hijos de la señora son amigos del entonces el los envió donde la doctora y le mandaron unas inyecciones y pegaba unos gritos cuando le ponían la inyección.

Title: Subtiava-1.docx

Descriptor Info: Age: Interview Location: Residence: Interviewer: Date of Interview:

Codes Applied: Promiscuity

Excerpt Package: 17300

Excerpt End: 17988

HANNAH: Piensa que el cáncer de cuello uterino podría afectarle en el futuro?

MUJER: Podría ser posible porque uno nunca sabe, yo estoy sola, no tengo a nadie pero talvez en un futuro por Dios pueda ser que aparezca alguien y no sé si podría ser infiel o si ya tenga antecedentes de problemas de salud sexual y pueda ser de casualidad me la pase a mi entonces corremos el riesgo pero claro uno tiene que ver que no es con cualquier persona que lo ira a hacer ni tampoco con cualquier persona es que uno va a entregarse.

HANNAH: Bueno y piensa que esta enfermedad es muy común?

MUJER: Yo pienso que si es común pero yo digo que solo sucede si uno anda de promiscuo y no solo con su pareja.

Title: Santa Ana-2.docx

Descriptor Info: Age: Interview Location: Residence: Interviewer: Date of Interview:

Codes Applied: Promiscuity

Excerpt Package: 14

Excerpt End: 524

ALEXANDRA: Entonces para empezar puede decirme un poco de su experiencia con el cuidado de salud en general?

MUJER: bueno, para iniciar yo por lo menos de mi misma me cuido, porque por esas enfermedades uno debe de cuidarse como el cáncer. el SIDA todo eso uno debe de cuidarse en las relaciones y estar solo con su pareja y a pesar de estar solo con la pareja también cuidarse porque uno nunca sabe que hacen a nuestras espaldas pero hasta la vez Gracias a Dios no he tenido ningún problema en ese particular.

Title: Santa Ana-2.docx

Descriptor Info: Age: Interview Location: Residence: Interviewer: Date of Interview:

Codes Applied: Promiscuity

Excerpt Package: 6182

Excerpt End: 6921

ALEXANDRA: Ahm, tiene usted algún familiar o amiga que haya sufrido del cáncer de cuello uterino?

MUJER: Una amiga tuve

ALEXANDRA: Y cómo fue su experiencia?

MUJER: Yo digo que fue doloroso porque al inicio yo no creí porque era una muchacha que se miraba bien y yo la conocía desde chiquita y era mi amiga porque estudio conmigo y nunca pensé que ella fuera a tener esa enfermedad porque yo no la miraba con hombres pero como usted sabe a veces uno nunca sabe pero fue una experiencia muy dura que me paso de tristeza cuando ella se murió de esa enfermedad porque ella no dilató mucho, todo fue que le dijeran que tenía eso, salió embarazada cuando tenía el cáncer y como a ella le estaban haciendo las quimioterapias se le cayo el niño.

Title: Perla-3.docx

Descriptor Info: Age: Interview Location: Residence: Interviewer: Date of Interview:

Codes Applied: Promiscuity

Excerpt Package: 6259

Excerpt End: 7062

MUJER: Mucha gente está informada sobre eso, porque en los centros de salud hay rótulos y hay que leerlos mientras toca la cita en la consulta pero muchas personas no le prestan importancia pero es muy importante porque ya ha habido muchas muertes sobre eso y hay muertes de mujeres que tienen la enfermedad del papiloma humano por lo mismo

HANNAH: Muerte por el virus o de otra cosa?

MUJER: No, de eso por el virus porque yo por ejemplo yo tengo una tía y mi tía tiene eso y ella no se recuperaba, esta delgada porque eso te enferma psicológicamente también.

HANNAH: Usted hablo un poquito de su tía, no sé si recuerda.

MUJER: Si, es decir ella no vive conmigo pero vive cerca de mi casa, pero si ella padece eso y ella es promiscua y entonces que le resulto, que le haya pasado eso porque no se cuida.

Title: Perla-2.docx

Descriptor Info: Age: Interview Location: Residence: Interviewer: Date of Interview:

Codes Applied: Promiscuity

Excerpt Package: 7511

Excerpt End: 7870

ALEXANDRA: Cree que hay una relación entre la prueba y la promiscuidad sexual? Por ejemplo si una chica se hace la prueba de pap será que una chica sea más propensa a tener relaciones sexuales?

MUJER: Pues ellas se sienten seguras que no tienen nada pero deben de cuidarse y con las relaciones sexuales se transmiten eso y no estará segura si no usa el condón

Title: Perla-4.docx

Descriptor Info: Age: Interview Location: Residence: Interviewer: Date of Interview:

Codes Applied: Promiscuity

Excerpt Package: 7252

Excerpt End: 7641

HANNAH: Y tiene algunas preocupaciones acerca de la salud sexual?

MUJER: Bueno, claro que si porque mire, mi pareja no vive aquí, vive fuera del país y usted sabe que uno no sabe si pueden traer una enfermedad porque allá pueden vivir con alguien más y después se lo vienen a transmitir a uno

HANNAH: Y su pareja puede regresar con esa enfermedad? Y por eso tiene miedo?

MUJER: Si, así es

Title: Los Leches-3.docx

Descriptor Info: Age: Interview Location: Residence: Interviewer: Date of Interview:

Codes Applied: Promiscuity

Excerpt Package: 2901

Excerpt End: 3152

SARA: Piensa que hay una relación entre la promiscuidad sexual y las pruebas? Por ejemplo si una joven se hace una prueba de pap y sale bien en ella será que esta muchacha se vuelva sexualmente activa?

MUJER: Uhm la verdad ahí no sé pero pienso que no

Title: Perla-3.docx

Descriptor Info: Age: Interview Location: Residence: Interviewer: Date of Interview:

Codes Applied: Promiscuity

Excerpt Package: 7603

Excerpt End: 8044

HANNAH: Y ha tenido una experiencia con el cáncer de cuello uterino, sabe de una persona que ha sufrido de cáncer?

MUJER: Mi mama.

HANNAH: Su madre? Oh lo siento mucho

MUJER: Si a ella le hicieron una cono biopsia es en el cuello, y después de eso le dieron tratamiento para ver qué pasaba y ella era una ama de casa que no salía a ningún lado pero resulta que mi Papa fue el de la culpa, como el salía y le gustaba andar con muchas mujeres.

Title: Mantica-2.docx

Descriptor Info: Age: Interview Location: Residence: Interviewer: Date of Interview:

Codes Applied: Promiscuity

Excerpt Package: 8283

Excerpt End: 8618

ALEXANDRA: Y cuáles son sus preocupaciones más grandes acerca de su salud en general

MUJER: Más que todo la parte intima porque a como están las cosas hoy en día hay que tener cuidado con sus partes íntimas saber con qué personas se tiene relaciones, hacerse exámenes, cuidarse mucho y encomendarse sobre todo a Dios porque es el único

Title: Santa Ana-3.docx

Descriptor Info: Age: Interview Location: Residence: Interviewer: Date of Interview:

Codes Applied: Promiscuity

Excerpt Package: 1954

Excerpt End: 2460

HANNAH: Cual piensa es la actitud en general de las mujeres acerca de las pruebas de pap?

MUJER: Bueno, yo siempre las invito a que se hagan la prueba de pap para ver si tenemos el cáncer de cuello uterino ya que muchas mujeres estamos padeciendo de esto, a veces hay algunas mujeres que piensan que el pap es para esas mujeres que han tenido muchas parejas entonces decimos que no, que el pap es para toditas para tener un mejor cuido tanto para las jóvenes como para la mujer ya adulta, es muy importante

Title: Los Leches-2.docx

Descriptor Info: Age: Interview Location: Residence: Interviewer: Date of Interview:

Codes Applied: Promiscuity

Excerpt Package: 4449

Excerpt End: 4603

HANNAH: Si piensa que es un virus muy común que puede afectarle en el futuro?

MUJER: Si porque si mi marido anda con muchas mujeres podría ser que me pase

Title: Subtiava-1.docx

Descriptor Info: Age: Interview Location: Residence: Interviewer: Date of Interview:

Codes Applied: Promiscuity

Excerpt Package: 17820

Excerpt End: 17988

HANNAH: Bueno y piensa que esta enfermedad es muy común?

MUJER: Yo pienso que si es común pero yo digo que solo sucede si uno anda de promiscuo y no solo con su pareja.

Title: Perla-5.docx

Descriptor Info: Age: Interview Location: Residence: Interviewer: Date of Interview:

Codes Applied: Promiscuity

Excerpt Package: 13981

Excerpt End: 14460

ALEXA: Cual es el tratamiento para que enfermedad?

MUJER: Es cuando talvez tengo una pequeña infección que usted sabe que a veces adquirimos porque usamos muy apretado el pantalón o el mismo organismo cuando tal vez hay algo que no le parece para protegerse el mismo y así comienza son situaciones pequeñas pero uno nunca sabe porque así puede comenzar, o tal vez mi pareja no es cien por ciento fiel uno nunca sabe pero aun así siempre debe de estar con el cuidado de uno mismo.

Title: Santa Ana-1.docx

Descriptor Info: Age: Interview Location: Residence: Interviewer: Date of Interview:

Codes Applied: Promiscuity

Excerpt Package: 3146

Excerpt End: 3388

SARA: Y piensa que hay una relación entre la promiscuidad sexual y las pruebas de pap? Por ejemplo que si una joven se hace una prueba de pap y ella sale bien en la prueba hará que ella se vuelva sexualmente activa

MUJER: Pues yo diría que no

Title: Poneloya-2.docx

Descriptor Info: Age: Interview Location: Residence: Interviewer: Date of Interview:

Codes Applied: Promiscuity

Excerpt Package: 7300

Excerpt End: 8291

ALEXA: Usted piensa que hay una relación entre la prueba y la promiscuidad sexual? Por ejemplo si una chica se hace la prueba de pap seria más propensa a tener relaciones?

MUJER: A veces ´por eso las enfermeras y los médicos quieren captar todas esas niñas que tienen relaciones sexuales porque como no hay una protección entre ellos, no hay un cuidado entonces ahí está previsto a cualquier enfermedad o infección que pueda transmitir ese niño a esa niña, claro porque esa niña no viene a hacerse ningún chequeo, entonces ella puede estar prevista a cualquier infección vaginal que se le puede presentar aquí se hizo una campaña hace cuatro años yo anduve en eso, trayendo a jóvenes que yo sabía tenían relaciones sexuales y vino un médico de fuera que era de León para que hiciera el pap junto con Aurorita y fue un éxito porque varias muchachas adolescentes tenían relaciones sexuales y eso es bueno porque así se captan las enfermedades porque hoy en día se dan muchas por la sexualidad.

Title: Perla-5.docx

Descriptor Info: Age: Interview Location: Residence: Interviewer: Date of Interview:

Codes Applied: Promiscuity

Excerpt Package: 12028

Excerpt End: 12849

ALEXA: Solo nos faltan unas preguntitas acerca del papiloma, como piensa que es la actitud de la comunidad acerca de esta enfermedad?

MUJER: Bueno eso le sucede más a los adolescentes bastante pero a como le digo es porque hay varones que andan con varias mujeres y talvez le transmiten esa enfermedad a su esposa o compañera y no le gusta que vaya a un centro de salud a tratar la enfermedad porque son personas machistas pero le estoy hablando de lugares talvez todavía del campo o comarcas y que todavía se dan esas situaciones porque es bastante tabú y aquí en la ciudad se da en los adolescentes que comienzan a tener relaciones sexuales y tal vez por pena no quieren ir a los centros de salud tal vez las muchachas y también los varones porque para ellos no es común es más común ver a las mujeres ir a ginecología.

Title: Mantica-1.docx

Descriptor Info: Age: Interview Location: Residence: Interviewer: Date of Interview:

Codes Applied: Promiscuity

Excerpt Package: 3554

Excerpt End: 4911

Sara: Si tiene confianza, piensa usted que hay una relación de la prueba y la promiscuidad sexual, por ejemplo si una chica adolescente tiene la prueba va a ser más propensa a tener relaciones sexuales?

Mujer: Como, que ella se haga la prueba del pap y que tenga relaciones sexuales con su pareja?

Sara: O con algunas personas?

Mujer: Yo digo que no se, creo que si.

Sara: Usted dice si, por que?

Mujer: Es que no le entiendo

Sara: Oh lo siento, otra vez, si una chica tiene la prueba de pap, una chica de diecisiete años, después de tener la prueba de pap va a ser más propensa de tener relaciones sexuales porque está segura que no tiene enfermedades o algo mas?

Mujer: Entonces usted me está diciendo que si una menor de edad va a hacerse el pap y ella miro que salió sin ninguna enfermedad y comienza a tener relaciones sexuales va a sentirse ella que no se va a enfermar de nada?

Sara: Si, verdad, que piensa?

Mujer: Si yo fuera así, yo pienso que no hay que confiarse también, porque usted sabe ahora las enfermedades pero si uno está haciéndose las pruebas yo me sentiría así, en paz si yo me estoy cuidando y hago todo lo que me dicen en el centro de salud y sigo las orientaciones correctas, que me cuide y todo eso, yo digo que si no me preocuparía de las enfermedades porque me estoy cuidando, haciéndome el pap y eso pero yo, no se otra persona

Title: Mantica-2.docx

Descriptor Info: Age: Interview Location: Residence: Interviewer: Date of Interview:

Codes Applied: Promiscuity

Excerpt Package: 4288

Excerpt End: 4569

ALEXANDRA: Ha oído hablar del papiloma

MUJER: Sí, más o menos

ALEXANDRA: Y usted piensa que hay un riesgo de esto para las mujeres

MUJER: Yo digo que sí, porque vi en internet que esto se pasa por las relaciones sexuales entonces digo yo que si hay un riesgo para todas las mujeres

Title: Los Leches-2.docx

Descriptor Info: Age: Interview Location: Residence: Interviewer: Date of Interview:

Codes Applied: Promiscuity

Excerpt Package: 424

Excerpt End: 748

HANNAH: Dígame acerca de su experiencia sobre la salud sexual y la educación sexual?

MUJER: Sobre la sexualidad?

HANNAH: Si, acerca de la educación de la salud sexual?

MUJER: Bueno ahí no sé yo, con lo mío, he estado bien porque no he tenido problemas con mi marido, pero con las demás parejas no se ahí si no puedo explicar

Title: Subtiava-1.docx

Descriptor Info: Age: Interview Location: Residence: Interviewer: Date of Interview:

Codes Applied: Promiscuity

Excerpt Package: 9362

Excerpt End: 10486

HANNAH: Ha sufrido de cáncer de cuello uterino su hermana?

MUJER: Yo digo que sí, porque ella dice que la doctora le ha dicho y le ha mandado tratamiento y todo, y se le ha compuesto, incluso en estos días ella anduvo donde la doctora y le pregunto a otra doctora nueva y ella le dijo que va a vivir con eso toda la vida, además el caso es como que el útero se le ha puesto chiquito y eso debe de ser porque ya le va a venir la menopausia pero ella es menor que yo todavía entonces digo yo que no es posible porque está muy joven todavía.

HANNAH: Y ella recibió tratamiento por el problema?

MUJER: Si, ella está en tratamiento.

HANNAH: Y como fue el tratamiento?

MUJER: Inyecciones, pastillas y óvulos.

HANNAH: Y como encontró esta enfermedad?

MUJER: Ella se sentía humedad, sangrado, y me imagino que ardor y dolor.

HANNAH: Ella recibió una prueba de pap?

MUJER: Si y como ella tenía un marido muy vago entonces yo digo que por esa parte él se lo debe de haber pasado porque el padecía mucho de los riñones, eso y otras cosas y me imagino que ellos lo hacían sin protegerse y como él era vago entonces él se lo paso a ella.

Title: Mantica-2.docx

Descriptor Info: Age: Interview Location: Residence: Interviewer: Date of Interview:

Codes Applied: Promiscuity

Excerpt Package: 3676

Excerpt End: 4287

ALEXANDRA: Ha oído mucho del cáncer cervical l cáncer de cuello uterino

MUJER: No, casi no he oído de eso

ALEXANDRA: Usted piensa que hay un riesgo para todas las mujeres con esa enfermedad

MUJER: Yo digo que si

ALEXANDRA: Y usted piensa que puede afectarle en el futuro+

MUJER: Yo digo que no se solo dios porque uno se cuida tal vez no, porque a veces eso se da por las relaciones sexuales entonces uno solo tiene que cuidarse, por lo menos mi marido no es santo, cualquier momento puede fallar pero yo le digo a mi marido que si hará algo que use condón

ALEXANDRA: De esa manera se protege

MUJER: Si, así es

Title: Perla-3.docx

Descriptor Info: Age: Interview Location: Residence: Interviewer: Date of Interview:

Codes Applied: Promiscuity

Excerpt Package: 6259

Excerpt End: 7062

MUJER: Mucha gente está informada sobre eso, porque en los centros de salud hay rótulos y hay que leerlos mientras toca la cita en la consulta pero muchas personas no le prestan importancia pero es muy importante porque ya ha habido muchas muertes sobre eso y hay muertes de mujeres que tienen la enfermedad del papiloma humano por lo mismo

HANNAH: Muerte por el virus o de otra cosa?

MUJER: No, de eso por el virus porque yo por ejemplo yo tengo una tía y mi tía tiene eso y ella no se recuperaba, esta delgada porque eso te enferma psicológicamente también.

HANNAH: Usted hablo un poquito de su tía, no sé si recuerda.

MUJER: Si, es decir ella no vive conmigo pero vive cerca de mi casa, pero si ella padece eso y ella es promiscua y entonces que le resulto, que le haya pasado eso porque no se cuida.

Title: Perla-4.docx

Descriptor Info: Age: Interview Location: Residence: Interviewer: Date of Interview:

Codes Applied: Promiscuity

Excerpt Package: 6446

Excerpt End: 7251

HANNAH: Y cómo piensa es la actitud de las mujeres en la comunidad acerca del cáncer de cuello uterino?

MUJER: Bueno lo que pasa es que siempre hay mujeres que les da pena andar en el medico y eso se da cuando los maridos andan detrás de las mujeres porque no quieren que ni los médicos las miren siempre se da eso, yo tengo una amiga que apenas fue al médico y recién se dio cuenta que tiene el virus y su marido se molestó con ella porque se dejó ver de un hombre, entonces yo le dije que le dijera que le pague para que vaya donde una doctora

HANNAH: Ella no quiere recibir una prueba de pap de un médico masculino y por eso no quiere venir?

MUJER: Exactamente pero ella ya se lo hizo y ella se lo comento a su marido y parece que se molestó, que locura le dije yo, pero bueno cada quien con sus ideas

Title: Perla-3.docx

Descriptor Info: Age: Interview Location: Residence: Interviewer: Date of Interview:

Codes Applied: Promiscuity HPV

Excerpt Package: 5105

Excerpt End: 5647

HANNAH: Y ha oído sobre el virus de papiloma humano alguna vez?

MUJER: Si, si he oído

HANNAH: Como percibe el riesgo de este virus?

MUJER: Me imagino se da por las relaciones sexuales sin protección, porque me imagino que las personas que reciben esa enfermedad es porque son promiscuas, no importa tanto esas personas sino también las mujeres de hogar porque si los hombres son promiscuos y nosotros estamos en las casas tranquilas y ellos vienen y nos pasan esa enfermedad, por irresponsabilidad de la persona que anda haciendo esos actos.

Title: Perla-3.docx

Descriptor Info: Age: Interview Location: Residence: Interviewer: Date of Interview:

Codes Applied: Promiscuity HPV

Excerpt Package: 5105

Excerpt End: 5647

HANNAH: Y ha oído sobre el virus de papiloma humano alguna vez?

MUJER: Si, si he oído

HANNAH: Como percibe el riesgo de este virus?

MUJER: Me imagino se da por las relaciones sexuales sin protección, porque me imagino que las personas que reciben esa enfermedad es porque son promiscuas, no importa tanto esas personas sino también las mujeres de hogar porque si los hombres son promiscuos y nosotros estamos en las casas tranquilas y ellos vienen y nos pasan esa enfermedad, por irresponsabilidad de la persona que anda haciendo esos actos.

Title: Los Leches-2.docx

Descriptor Info: Age: Interview Location: Residence: Interviewer: Date of Interview:

Codes Applied: Promiscuity HPV

Excerpt Package: 2444

Excerpt End: 2765

HANNAH: Ha oído del virus de papiloma humano o vph alguna vez?

MUJER: Si he oído, decir de todo

HANNAH: Puede hablarme un poco acerca de este virus?

MUJER: Lo que yo he oído acerca de la prueba de esto es que es cuando el varón anda con muchas mujeres y se lo pasa a su esposa oficial eso es lo que he escuchado decir yo

Title: Mantica-3.docx

Descriptor Info: Age: Interview Location: Residence: Interviewer: Date of Interview:

Codes Applied: Promiscuity Pap smear

Excerpt Package: 5692

Excerpt End: 6291

HANNAH: Cuando piensa usted que una chica o una mujer debe iniciar a tener las pruebas de pap?

MUJER: Bueno yo creo que cuando se es una mujer adulta porque una niña no puede ver a otro niño, eso se debe hacer cuando ya ha estudiado y se ha preparado y después los hijos

HANNAH: Piensa usted que una chica o una mujer debe iniciar las pruebas de pap después de empezar las relaciones o piensa que la edad es importante para tener una prueba de pap?

MUJER: Yo digo que sí, la edad es importante, porque imagínese una niña de doce años teniendo un bebe, hoy en día es común pero no debería de ser así.

Title: Poneloya-1.docx

Descriptor Info: Age: Interview Location: Residence: Interviewer: Date of Interview:

Codes Applied: Promiscuity Pap smear Time

Excerpt Package: 4390

Excerpt End: 5204

ALEXANDRA: Por ejemplo si antes de recibir una prueba de Pap usted consultaría con su pareja?

MUJER: Prueba de pap? Es esa de Papanicolaou? Consulta si me la puedo hacer? No, no lo haría porque es algo sobre mi cuerpo y tengo entendido que se debe de hacer una vez al año, y si yo me quiero a mi misma y mi cuerpo debo hacerlo porque tengo entendido que las mujeres de aquí no les gusta hacérselo, porque no tienen tiempo porque deben cuidar sus casas, y eso es algo malo porque deben hacerlo para prevenir muchas enfermedades como cáncer, entre otras muchas cosas como las enfermedades de transmisión sexual, a como yo pienso la mayor parte de las mujeres con enfermedades sexuales son las del hogar porque sus esposos andan con muchas mujeres y las del hogar no se cuidan porque ellas solo están con sus maridos.

**Sexual Education**

Title: Mantica-6.docx

Descriptor Info: Age: Interview Location: Residence: Interviewer: Date of Interview:

Codes Applied: Sexual education

Excerpt Package: 9051

Excerpt End: 9661

ALEXANDRA: Y hay algo más acerca del tema de las mujeres o la salud que quieres discutir o compartir?

MUJER: Solamente que asistamos a los centros de salud por que es donde obtenemos la primera información por que la tecnología también ayuda pero en los centros es donde la información más eficaz porque son ellos los que la están estudiando y aunque la tecnología no se queda atrás pero en los centros es donde uno aprende más con los médicos y uno se siente más tranquilo

ALEXANDRA: Y por esta razón es que es más difícil comunicarse con las mujeres del campo?

MUJER: Sí, es más difícil comunicarse con ellas

Title: Mantica-6.docx

Descriptor Info: Age: Interview Location: Residence: Interviewer: Date of Interview:

Codes Applied: Sexual education

Excerpt Package: 8156

Excerpt End: 8817

ALEXANDRA: Y de donde le gusta a usted recibir información acerca de su salud?

MUJER: En los centros de salud y si hay algo que no entiendo entro a internet e investigo para tener más información actual, así como las que nos dan en las charlas los médicos, cuando andan casa a casa y cuando uno viene aquí a los centros ellos ponen y dan información ellos siempre están informando a la población pero a las que venimos a los centros de salud pero las que no vienen no sé cómo se darán cuenta

ALEXANDRA: Usted cree que tiene suficiente información para tomar decisiones acerca de su salud, por ejemplo si debe tener una prueba de pap o no?

MUJER: Yo digo que si

Title: Mantica-1.docx

Descriptor Info: Age: Interview Location: Residence: Interviewer: Date of Interview:

Codes Applied: Sexual education

Excerpt Package: 7810

Excerpt End: 8236

Mujer: Yo no tengo pareja soy sola si pero cuando voy al centro yo pregunto cosas antes iba a un proyecto que se llama Mery barreda y mucho me decían sobre eso de las relaciones y todo eso, pero era cuando estaba pequeña así que ya no me acuerdo casi de nada pero nos decían que nos cuidáramos y protegiéramos y que visitáramos los centros que cuando tuviéramos nuestra primera pareja preguntáramos pero ya casi no me acuerdo.

Title: Mantica-6.docx

Descriptor Info: Age: Interview Location: Residence: Interviewer: Date of Interview:

Codes Applied: Sexual education

Excerpt Package: 777

Excerpt End: 1575

ALEXANDRA: Y usted también puede recibir la salud sexual, tratamiento y consejos aquí?

MUJER: Si aquí nos ayudan con la salud sexual de la mujer activa ya sea protección por medio del pap, una vez haciéndote el pap si te dan resultado y te sale algo te dan tratamiento y te siguen la secuencia hasta que sales de tu problema

ALEXANDRA: Y cree usted que las chicas adolescentes reciben suficiente información acerca de la salud sexual?

MUJER: Los puestos están en donde están y la gente los sale a buscar a veces hay charlas de estudiantes que siempre los andan monitoreando y las bases son los centros de salud pero la actividad sexual es responsabilidad de cada quien, cada quien cuida su higiene y siempre hay que visitar los centros de salud para estar activo y pendiente de cómo está su cuerpo

Title: Perla-2.docx

Descriptor Info: Age: Interview Location: Residence: Interviewer: Date of Interview:

Codes Applied: Sexual education

Excerpt Package: 8216

Excerpt End: 8622

ALEXANDRA: Y cree usted que ellos deben de recibir la información de algún otro medio aparte de la casa?

MUJER: Aquí

ALEXANDRA: En los centros? O como pueden los jóvenes obtener información si los padres no quieren hablar de estos temas con ellos? Como deben de hacer?

MUJER: Pues si están pequeños aún no hay que hablarles al respecto, pero si ya razonan pues ya hay que explicarles aunque sea poco a poco

Title: Subtiava-2.docx

Descriptor Info: Age: Interview Location: Residence: Interviewer: Date of Interview:

Codes Applied: Sexual education

Excerpt Package: 7121

Excerpt End: 7849

HANNAH: Si, es la verdad, piensa que hay diferencias de conocimientos entre las mujeres urbanas y rurales?

MUJER: Si, considero que si porque tal vez uno tiene más conocimiento si asiste al centro si va a clases y es importante, pero las mujeres del campo no salen de sus casas, solo se ponen a tener varios hijos y no leen ni se informan entonces por eso creo que tienen un poco más de conocimiento los urbanos que lo rural

HANNAH: Tiene algunas preocupaciones acerca de la salud sexual?

MUJER: No, no, porque como tengo mi pareja y tengo mucho tiempo de estar con él y a como le digo, leyendo informándose creo que uno se educa acerca de la sexualidad, pero si le tomo importancia a la salud, las enfermedades a los chequeos

Title: Perla-3.docx

Descriptor Info: Age: Interview Location: Residence: Interviewer: Date of Interview:

Codes Applied: Sexual education

Excerpt Package: 739

Excerpt End: 3137

HANNAH: Perfecto, y dígame un poco acerca de la salud sexual y la educación de la salud sexual?

MUJER: Pues para serle honesta yo digo que aquí hay poca salud sexual y poca educación sexual, porque muchas personas no lo tratan ni con educación ni con respeto la salud sexual sino que solo lo toman como un deporte y creo que la sexualidad es algo que deben de respetar las personas porque además de eso de no respetarlo por eso es que vienen muchas enfermedades y muchos embarazos no deseados y enfermedades no deseadas porque primero no se cuidan, no respetan al sexo opuesto y tercero porque agarran el sexo como un deporte a como le digo, lo toman como por diversión y en eso pienso yo que uno debe de tener cuidado.

HANNAH: De donde recibe la educación sexual?

MUJER: Pienso que los primeros que tienen que hacer la educación sexual son en el hogar por ejemplo los padres tienen que educar a sus hijos decirles realmente que el sexo no es cosa de juego sino que es algo que lo deben de tomar en serio y deben de respetarse ante todo con la persona del sexo opuesto porque al no hacerlo hay enfermedades, embarazos no deseados y enfermedades que no esperan

HANNAH: Y usted aprendió sobre estos temas en el colegio?

MUJER: Correcto pero en los colegios casi no hay es poca

HANNAH: Y porque piensa que es así?

MUJER: Porque como ahora los jóvenes todo lo miran como algo normal entonces ellos dicen van a internet, tienen las redes sociales entonces tienen más información que la que nosotros les podemos dar, entonces para que les vamos a dar esas charlas de educación sexual.

HANNAH: La mayoría de la información la tienen de internet piensa usted?

MUJER: Es correcto, entonces es por eso que me imagino yo que no dan esas clases de educación y salud sexual en los colegios.

HANNAH: Piensa que las chicas adolescentes y las mujeres hoy día tienen suficiente información acerca de la salud sexual?

MUJER: Quizás muchas, porque tienen temor de tener alguna enfermedad, de salir embarazadas pero otras no y por curiosidad les gusta andar.

HANNAH: Y como podría mejorar la información acerca de la salud sexual para las chicas adolescentes hoy día?

MUJER: Como le digo principalmente en el hogar, los padres tienen que decirles a los niños, niñas todos que tienen que tener mucha precaución porque hay muchos riesgos además de eso pienso yo que todo se debe de tomar con responsabilidad y seriedad

Title: SantaAna-4.docx.docx

Descriptor Info: Age: Interview Location: Residence: Interviewer: Date of Interview:

Codes Applied: Sexual education

Excerpt Package: 1533

Excerpt End: 2174

ALEXANDRA: Y hay charlas aquí o donde recibió su educación sexual?

MUJER: Si si hay, siempre venimos aquí a que nos dieran charlas de cosas de cáncer de las mamas y nos dicen que debemos estar pendiente de nuestras cosas en la salud.

ALEXANDRA: Y piensa que las chicas adolescentes tienen suficiente información acerca de su salud sexual?

MUJER: Bueno a veces la juventud uno le dice las cosas y no hacen caso hasta que se ven con las enfermedades, buscan a los médicos y no reflexionan en el instante

ALEXANDRA: Y hay algo que los centros o los médicos pueden mejorar el conocimiento de los jóvenes?

MUJER: Si yo digo que sí, hay que hacer

Title: Subtiava-2.docx

Descriptor Info: Age: Interview Location: Residence: Interviewer: Date of Interview:

Codes Applied: Sexual education

Excerpt Package: 658

Excerpt End: 1223

HANNAH: Dígame acerca de su experiencia con el cuidado de la salud sexual y la educación sexual?

MUJER: Bueno, cada vez que me tocaba venir a planificar para no quedar embarazada, asistía al centro a que me inyectaran y habían muchachos que daban charlas sobre educación sexual sobre las enfermedades de transmisión sexual siempre me atendieron con buena manera, y recibí la mayoría de las charlas aquí en el centro y las muchachas decían que debíamos de hacernos los exámenes año a año para no tener ningún tipo de enfermedad y poder prevenir algún tipo de cáncer.

Title: Subtiava-1.docx

Descriptor Info: Age: Interview Location: Residence: Interviewer: Date of Interview:

Codes Applied: Sexual education

Excerpt Package: 2441

Excerpt End: 3736

HANNAH: Bueno y hábleme un poco acerca de su experiencia con la salud sexual específicamente o la educación sexual?

MUJER: Gracias a Dios pues nunca he tenido problemas con respecto a eso y cuando he pasado con médicos a hacerme chequeos y eso los médicos me felicitan realmente porque no he tenido ningún problema de ese tipo hasta la fecha ,la verdad que me he cuidado mucho en ese particular.

HANNAH: Y ha recibido educación acerca de estos temas?

MUJER: No, hasta ahora.

HANNAH: Ni de los colegios?

MUJER: Bueno en los colegios si enseñan cosas de eso pero Gracias a Dios hasta ahora no he tenido ningún problema.

HANNAH: Piensa que quiere recibir más educación acerca de estos temas?

MUJER: Claro que sí, porque es muy bueno.

HANNAH: Como piensa usted que puede mejorar la educación de la salud sexual?

MUJER: Bueno, cuidarse, protegerse, porque si hay problemas no de parte de la mujer sino del varón tienen que cuidarse y protegerse porque si no después salen afectando a la mujer de su casa porque eso es un riesgo, así me paso una vez no a mi sino que a él y entonces yo le dije, mira que te paso y entonces dijo ve donde la doctora por mí, yo le dije yo no voy a ir, vas a ir tu porque tú eres el del problema , entonces como te paso eso, era porque el andaba con otras mujeres vagas.

Title: Perla-3.docx

Descriptor Info: Age: Interview Location: Residence: Interviewer: Date of Interview:

Codes Applied: Sexual education

Excerpt Package: 13823

Excerpt End: 13992

HANNAH: Que hace usted para mantener la salud sexual?

MUJER: Que hago? Pues venirme a estar chequeando, hacerme mi chequeo médico para ver porque realmente es necesario.

Title: Perla-3.docx

Descriptor Info: Age: Interview Location: Residence: Interviewer: Date of Interview:

Codes Applied: Sexual education

Excerpt Package: 739

Excerpt End: 3137

HANNAH: Perfecto, y dígame un poco acerca de la salud sexual y la educación de la salud sexual?

MUJER: Pues para serle honesta yo digo que aquí hay poca salud sexual y poca educación sexual, porque muchas personas no lo tratan ni con educación ni con respeto la salud sexual sino que solo lo toman como un deporte y creo que la sexualidad es algo que deben de respetar las personas porque además de eso de no respetarlo por eso es que vienen muchas enfermedades y muchos embarazos no deseados y enfermedades no deseadas porque primero no se cuidan, no respetan al sexo opuesto y tercero porque agarran el sexo como un deporte a como le digo, lo toman como por diversión y en eso pienso yo que uno debe de tener cuidado.

HANNAH: De donde recibe la educación sexual?

MUJER: Pienso que los primeros que tienen que hacer la educación sexual son en el hogar por ejemplo los padres tienen que educar a sus hijos decirles realmente que el sexo no es cosa de juego sino que es algo que lo deben de tomar en serio y deben de respetarse ante todo con la persona del sexo opuesto porque al no hacerlo hay enfermedades, embarazos no deseados y enfermedades que no esperan

HANNAH: Y usted aprendió sobre estos temas en el colegio?

MUJER: Correcto pero en los colegios casi no hay es poca

HANNAH: Y porque piensa que es así?

MUJER: Porque como ahora los jóvenes todo lo miran como algo normal entonces ellos dicen van a internet, tienen las redes sociales entonces tienen más información que la que nosotros les podemos dar, entonces para que les vamos a dar esas charlas de educación sexual.

HANNAH: La mayoría de la información la tienen de internet piensa usted?

MUJER: Es correcto, entonces es por eso que me imagino yo que no dan esas clases de educación y salud sexual en los colegios.

HANNAH: Piensa que las chicas adolescentes y las mujeres hoy día tienen suficiente información acerca de la salud sexual?

MUJER: Quizás muchas, porque tienen temor de tener alguna enfermedad, de salir embarazadas pero otras no y por curiosidad les gusta andar.

HANNAH: Y como podría mejorar la información acerca de la salud sexual para las chicas adolescentes hoy día?

MUJER: Como le digo principalmente en el hogar, los padres tienen que decirles a los niños, niñas todos que tienen que tener mucha precaución porque hay muchos riesgos además de eso pienso yo que todo se debe de tomar con responsabilidad y seriedad

Title: Mantica-1.docx

Descriptor Info: Age: Interview Location: Residence: Interviewer: Date of Interview:

Codes Applied: Sexual education

Excerpt Package: 1079

Excerpt End: 2161

Sara: Nunca, digame su experiencia acerca de la salud sexual y la educación sexual, ha recibido educación sexual?

Mujer: Si, he recibido

Sara: Donde ha recibido?

Mujer: Aquí en el centro de salud donde yo visito, si he recibido, siempre que voy a pasar consulta que llevo al chiquito me dicen las cosas de lo que hay que cuidarse, que hay que protegerse mas de las enfermedades que están ahora, siempre te dan charlas.

Sara: Ha recibido educación del colegio?

Mujer: Si, en el colegio también.

Sara: Y de familia?

Mujer: También de familia.

Sara: Donde pueden las mujeres recibir educación sobre su salud sexual

Mujer: Yo digo que en los centros de salud

Sara: Piensa que las chicas adolescentes tienen suficiente información acerca de su salud sexual y educación

Mujer: Que hay que recibirla las menores de edad?

Sara: Si

Mujer: Si hay que recibir porque ahora a cómo está la vida, esta complicada, pero como ahora hay muchas personas que dan charlas todo eso, ahora saben más, antes tal vez no sabían pero ahora saben bastante, Gracias a Dios a mí me han atendido bien en todo eso

Title: Santa Ana-2.docx

Descriptor Info: Age: Interview Location: Residence: Interviewer: Date of Interview:

Codes Applied: Sexual education

Excerpt Package: 7430

Excerpt End: 7654

ALEXANDRA: Y cuales precauciones toma usted para prevenir estas enfermedades para mantenerse segura?

MUJER: El condón, tiene que usarlo siempre uno para poder rechazar todo tipo de enfermedades es lo más importante el condón

Title: Perla-2.docx

Descriptor Info: Age: Interview Location: Residence: Interviewer: Date of Interview:

Codes Applied: Sexual education

Excerpt Package: 1932

Excerpt End: 2611

ALEXANDRA: Y a donde ha recibido la educación sobre su salud sexual?

MUJER: Allá en la Casona, es que allá en la Casona dan también consultas pero el examen que le harán a mi hija es acá entonces teníamos que venir aquí

ALEXANDRA: Y cree usted que las chicas adolescentes y los jóvenes reciben suficiente información acerca de su salud?

MUJER: No porque al menos ahorita mi hija está preguntándome que es eso que le harán, porque es una biopsia entonces yo le digo que ni idea, que no sé, pero es para no asustarla pero mi mama murió de cáncer y yo no quiero asustarla a ella entonces yo le digo que no sé qué le harán pero es solo para no asustarla aunque yo ya más o menos se.

Title: Los Leches-3.docx

Descriptor Info: Age: Interview Location: Residence: Interviewer: Date of Interview:

Codes Applied: Sexual education

Excerpt Package: 1354

Excerpt End: 1878

SARA: Donde pueden las mujeres recibir educación sexual?

MUJER: No se la verdad, creo que aquí

SARA: En charlas?

MUJER: Si cuando dan las enfermeras la Brenda o cualquier otra de aquí del centro y en esos momentos he recibido

SARA: Piensa que las chicas adolescentes tienen suficiente información acerca de su educación salud sexual?

MUJER: Yo digo que depende el colegio algunos lo dan otros no y a veces llegan a dar a los colegios otras personas, en mi opinión yo pienso que es bueno que les den charlas a los estudiantes

Title: Santa Ana-2.docx

Descriptor Info: Age: Interview Location: Residence: Interviewer: Date of Interview:

Codes Applied: Sexual education

Excerpt Package: 1784

Excerpt End: 2931

ALEXANDRA: Y hay charlas aquí, acerca de la salud sexual?

MUJER: Si hacen charlas

ALEXANDRA: Y piensa usted que hay otros medios en donde los jóvenes puedan recibir educación sexual?

MUJER: Si, porque por lo menos yo tengo tres hijos, dos ya se me casaron pero tengo uno aun conmigo que tiene diecinueve años y recibe charlas en el colegio porque está en tercer año de secundaria y ahí les dan, además yo hablo con él porque yo no quiero que el día de mañana le vaya a suceder algo, no solo a nosotras las mujeres nos pasa sino también a los varones, por eso yo lo aconsejo a mi hijo en ese particular.

ALEXANDRA: Y piensa que los jóvenes reciben suficiente información acerca de la salud sexual?

MUJER: Yo digo de que si tanto nosotros como ellos, porque el cuándo llega a casa él me cuenta fíjese mama que nos dijeron varias cosas como esto y demás, en los colegios los orientan bastante, por lo menos por ejemplo el domingo nosotros tenemos una actividad aquí en la bocana de Poneloya que es acerca del SIDA, yo ya he recibido capacitaciones acerca del cáncer de mama porque siempre que invitan a eso voy porque es importante estar informados.

Title: Los Leches-3.docx

Descriptor Info: Age: Interview Location: Residence: Interviewer: Date of Interview:

Codes Applied: Sexual education

Excerpt Package: 5349

Excerpt End: 5971

SARA: Que hace usted para mantener la salud y seguridad contra enfermedades de transmisión sexual?

MUJER: Bueno primeramente me cuido, y no tener relaciones sexuales con cualquiera porque primero hay que conocerlo y evitar lo más que se puede las infecciones a veces las infecciones en la parte de uno vienen por el cuido de la ropa porque no se baña bien uno o porque no lava bien el calzón porque a veces uno piensa que solo vienen de sexo pero no a veces algunas infecciones vienen de eso porque por lo menos mi niña padece mucho de eso y ella no es una niña que ande con esas cosas pero yo supongo que es por el calzón

Title: Poneloya-2.docx

Descriptor Info: Age: Interview Location: Residence: Interviewer: Date of Interview:

Codes Applied: Sexual education

Excerpt Package: 2895

Excerpt End: 4384

ALEXA: Y puede decirme su experiencia con el cuidado de la salud sexual? De donde ha recibido información?

MUJER: Como? A dar o como mi persona? Es que yo soy mujer soltera no tengo.

ALEXA: Recibió información sobre esos temas en el colegio u otro lugar?

MUJER: Antes yo iba con la doctora a dar esos temas en el colegio, respecto de la sexualidad, porque aquí hay muchas adolescentes embarazadas yo iba y acompañaba a la doctora pero ya últimamente no he ido con ella pero es así porque yo soy brigadista de salud y siempre y cuando iba acompañarla, incluso ayer me toco vacunaciones de canino con otra señora que está aquí.

ALEXA: Y piensa usted que las chicas adolescentes reciben suficiente información?

MUJER: Yo digo que se requiere más principalmente en los centros de estudios porque es donde se agrupan más los adolescentes ahí es bonito porque aquí a veces no le prestan atención cuando están hablando las enfermeras o los médicos otros están hablando y no escuchan lo que el medico está diciendo y a veces uno lo toma como que no vale aquello pero eso es bueno porque tener hijos a temprana edad no es bueno en mi concepto como mujer

ALEXA: Y hay algo que los médicos o los centros pueden hacer para mejorar el conocimiento de las chicas adolescentes?

MUJER: Para mí sería bueno impartir charlas y las que ya son mujeres sexualmente activas estar pendientes de la planificación, usar preservativos porque de otra manera no se puede detener los embarazos de jóvenes adolescentes.

Title: Santa Ana-1.docx

Descriptor Info: Age: Interview Location: Residence: Interviewer: Date of Interview:

Codes Applied: Sexual education

Excerpt Package: 5611

Excerpt End: 5918

SARA: Que hace para mantener la salud y seguridad de la salud contra enfermedades de transmisión sexual

MUJER: Pues a como le digo tomando las medidas preventivas y siguiendo los consejos de los doctores, usando protección y todas esas cosas.

SARA: Y que métodos de prevención utiliza

MUJER: Condones claro

Title: Perla-5.docx

Descriptor Info: Age: Interview Location: Residence: Interviewer: Date of Interview:

Codes Applied: Sexual education

Excerpt Package: 3715

Excerpt End: 5624

ALEXA: Y de dónde ha recibido usted la educación sexual o sobre la salud sexual?

MUJER: Bueno, primero en la casa de mis padres y después la escuela y después en la universidad ya que a uno lo van capacitando.

ALEXA: Usted piensa que las jóvenes reciben suficiente información?

MUJER: Todavía hay tabú sobre eso, bastante tabú, usted sabe que aquí en Nicaragua todavía es una sociedad que es bastante tradicional, bastante cultural, entonces hay bastante tabú sobre eso todavía, sobre la información acerca de la sexualidad tal vez en lugares más rurales, como en comarcas tal vez ellos son más cerrados, hay bastante machismo entonces se considera que aunque estamos en un siglo XXI es bastante cerrado considero yo sobre eso, porque ahorita se ha aumentado la tasa de mortalidad sobre las mujeres, se escucha en las noticias entonces tal vez se necesita bastante educación porque es eso tanto en la casa que es donde uno comienza, entonces ahí ya se va pasando de generación en generación de que las mujeres tienen que atender al esposo, el marido es todo, entonces así vamos y ya el niño y la niña también vienen adoptando esa postura de que no somos iguales, de que él o ella puede hacer una cosa mientras el otro hace otra, que si yo estoy enferma un día, talvez tú me atiendes, algo así reciproco, entonces falta esa educación, yo creo que todavía nos falta como un cincuenta por ciento porque a como le digo hay bastante tabú nuestra sociedad es bastante tradicional y también la tecnología afecta bastante.

ALEXA: Para mejorar la salud?

MUJER; Es que así como mejora también perjudica porque hay páginas que no se deberían de abrir y usted sabe para los jóvenes es bastante accesible, una Tablet, un Smartphone ahora es accesible para que ellos accedan a cosas que no deben, no solo a información que nos enriquezca sino que ya a niños ya están con ese conocimiento muy explícito que no debería de ser

Title: Santa Ana-1.docx

Descriptor Info: Age: Interview Location: Residence: Interviewer: Date of Interview:

Codes Applied: Sexual education

Excerpt Package: 700

Excerpt End: 2075

SARA; Dígame acerca de su experiencia con la salud sexual y educación sexual? Ha recibido educación sexual

MUJER: Pues la verdad si porque nos han enseñado como prevenir muchas enfermedades sexuales y los tipos de anticonceptivos todas esas cosas que lo protegen a uno

SARA: Y donde ha recibido educación sexual

MUJER: Bueno eso lo hemos aprendido en el colegio

SARA: Colegio? Y en los centros, en charlas

MUJER: Si

SARA: Y de su familia

MUJER: También

SARA: Desde pequeño, adulto

MUJER: Desde adolescente nos vienen enseñado eso para que tengamos mucho cuidado

SARA: Donde pueden las mujeres recibir educación sobre la salud sexual

MUJER: Bueno a como le acabo de comentar en los centros de salud en los colegios en folleto

SARA: En los mismo lugares?

MUJER: Si, en los mismos

SARA: Cree que las chicas adolescentes, tienen suficiente información sobre la salud sexual?

MUJER: Pues la verdad yo digo que sí, podría ser, pero muchas jóvenes salen embarazadas a mi corta edad y dejan todo en si vida como sus estudios y se preocupan más por tener una familia nada más.

SARA: Y como podría mejorar el conocimiento de las chicas y mujeres acerca de la salud sexual?

MUJER: Bueno yo creo que sería bueno que impartieran más charlas que convocaran a las jóvenes que hicieran un comité e informaran a las muchachas lo que puede sucederles y si ya ellas no acatan las informaciones

Title: Mantica-4.docx

Descriptor Info: Age: Interview Location: Residence: Interviewer: Date of Interview:

Codes Applied: Sexual education

Excerpt Package: 3921

Excerpt End: 4484

HANNAH: Y piensa que antes de su diagnostico ha recibido suficiente información para hacer decisiones sobre su salud sexual?

MUJER: Si, por lo menos por mi situación me dijeron que no podía tener relaciones sin el preservativo, sin usar el método y aun así siempre aunque estuviera con el preservativo el problema no se iba a detener, entonces eso es lo que estoy viviendo una vida muy pésima

HANNAH: Donde ha recibido educación sexual, del colegio, del cetro de salud o de su familia?

MUJER: Del Ministerio de Salud

HANNAH: De aquí del Centro de Salud?

MUJER: Si

Title: Mantica-3.docx

Descriptor Info: Age: Interview Location: Residence: Interviewer: Date of Interview:

Codes Applied: Sexual education

Excerpt Package: 4804

Excerpt End: 5187

HANNAH: Y donde ha recibido educación sexual, en el colegio o dónde?

MUJER: Bueno, antes era en el colegio y en los puestos médicos, cuando venimos a veces hay esas orientaciones acerca del cáncer y más ahora que se habla mucho del cáncer en las mujeres, cuando se viene a los puestos médicos hay bastante información sobre eso y aquí he escuchado además cuando estudie en la escuela

Title: Mantica-4.docx

Descriptor Info: Age: Interview Location: Residence: Interviewer: Date of Interview:

Codes Applied: Sexual education

Excerpt Package: 3921

Excerpt End: 4484

HANNAH: Y piensa que antes de su diagnostico ha recibido suficiente información para hacer decisiones sobre su salud sexual?

MUJER: Si, por lo menos por mi situación me dijeron que no podía tener relaciones sin el preservativo, sin usar el método y aun así siempre aunque estuviera con el preservativo el problema no se iba a detener, entonces eso es lo que estoy viviendo una vida muy pésima

HANNAH: Donde ha recibido educación sexual, del colegio, del cetro de salud o de su familia?

MUJER: Del Ministerio de Salud

HANNAH: De aquí del Centro de Salud?

MUJER: Si

Title: Santa Ana-3.docx

Descriptor Info: Age: Interview Location: Residence: Interviewer: Date of Interview:

Codes Applied: Sexual education

Excerpt Package: 700

Excerpt End: 1625

HANNAH: Si, bueno, gracias, dígame un poco acerca de su experiencia con la educación sexual?

MUJER: Bueno la educación de salud sexual está en que por ejemplo que uno se debe de proteger porque hay muchas enfermedades como el virus de papiloma humano y el VIH por eso hay que protegerse y cuidarse porque además no solamente las pueden transmitir por el sexo, también se puede transmitir si una persona que está infectada con VIH se corta y hacen contacto de sangre con sangre se pueden transmitir o una aguja.

HANNAH: Y de dónde aprendió sobre estos temas?

MUJER: Bueno, en los colegios aprendemos, las charlas que va haciendo el centro de salud que las dan constantemente sobre las enfermedades virales como las podemos evitar y que debemos hacer para tener un mejor cuidado

HANNAH: Y la información sobre le vph y el cáncer de cuello uterino lo ha tenido del colegio?

MUJER: No, del centro de salud de las charlas que dan

Title: Poneloya-1.docx

Descriptor Info: Age: Interview Location: Residence: Interviewer: Date of Interview:

Codes Applied: Sexual education

Excerpt Package: 5205

Excerpt End: 5938

ALEXANDRA: Y la religión influye en sus decisiones acerca de su salud?

MUJER: Bueno, yo no sé, pero creo que no porque si a una cristiana le gusta alguien ella lo hace y ya, pero no sé, yo creo que lo que más influye es la crianza de los padres, porque en mi caso yo tengo seis hermanas mujeres y dos varones y nuestra mama siempre nos aconseja, ella trabaja mucho y casi no se mantiene en casa pero cuando está con nosotros nos aconseja y nos dice que debemos cuidarnos mucho de eso, porque los muchachos de hoy en día tienen sexo con muchas y no se cuidan y las que sufren las consecuencias en muchas ocasiones son las mujeres pero también hay mujeres que les gusta andar así con varios hombres y viceversa contagian a los varones.

Title: Los Leches-1 (1).docx

Descriptor Info: Age: Interview Location: Residence: Interviewer: Date of Interview:

Codes Applied: Sexual education

Excerpt Package: 1501

Excerpt End: 2280

Sara: Where woman’s can receive sex education? Do you know?

(The woman was asking about information to her son)

Woman: I think that here.

Sara: Here, here in the center, do you have sons and daughters?

Woman: Yes, I have five, three men and two women, one still live with me at the house, and the others live with their husbands.

Sara: Do you think that the teenagers have enough information about sex education and general health?

Woman: I think that now they know because before they don’t.

Sara: What change that they have information now and not before?

Woman: (Can you give me an idea) that before we didn’t have any method like now, one had the children one after another each year, now no, now they just have one or two, because they have methods to not keep having kids.

Title: Poneloya-1.docx

Descriptor Info: Age: Interview Location: Residence: Interviewer: Date of Interview:

Codes Applied: Sexual education

Excerpt Package: 3775

Excerpt End: 4268

ALEXANDRA: Y cuales son sus preocupaciones más grandes acerca de su salud?

MUJER: Mías? Actualmente? Pues l verdad no tengo, porque yo no tengo novio, marido con quien tenga relaciones y no he tenido relaciones, además yo pienso que yo misma debo cuidar mi cuerpo porque si voy a estar con una persona no sé si el está solo conmigo o alguien más tengo que cuidarme y para eso hay muchos métodos y que debo de querer primero mi cuerpo y cuidarlo para cuando yo piense tener relaciones sexuales.

Title: Poneloya-1.docx

Descriptor Info: Age: Interview Location: Residence: Interviewer: Date of Interview:

Codes Applied: Sexual education

Excerpt Package: 978

Excerpt End: 2080

ALEXANDRA: Puede decirme acerca de su educación en salud sexual aquí en la comunidad o en los centros de salud?

MUJER: Bueno, aquí no sé, porque a como le digo casi no visito los centros de salud, pero donde si dan bastante información es en los colegios ahí nos dan información, en la secundaria nos dicen sobre cómo cuidarnos cuando se tiene relaciones sexuales y demás

ALEXANDRA: Y piensa que las chicas adolescentes reciben suficiente información acerca de su salud?

MUJER: Sexual? Yo pienso que sí, porque cuando llegan a dar charlas se tiene la opción de preguntar todo lo que quieras y si te da pena preguntar lo puedes investigar en internet, actualmente hay muchos sitios donde investigar

ALEXANDRA: En su opinión, hay algo que los médicos y los centros de salud puedan hacer para mejorar el conocimiento de los jóvenes?

MUJER: Es que normalmente lo que yo sé es que aquí dan charlas sobre la sexualidad, pero a veces es uno mismo el que no quiere venir a escuchar porque creemos que eso es algo loco, porque uno se informa mucho y es aún mejor cuando una chica no tiene marido podría decirse.

Title: Mantica-5.docx

Descriptor Info: Age: Interview Location: Residence: Interviewer: Date of Interview:

Codes Applied: Sexual education

Excerpt Package: 529

Excerpt End: 1031

HANNAH: Y puede hablar un poco sobre sus experiencias acerca de la salud sexual y la educación sexual?

MUJER: Bueno, educación sexual aquí nos han atendido bien y nos dan charlas

HANNAH: La mayoría de su educación sexual, ha sido de charlas aquí o del colegio?

MUJER: Del colegio también y de aquí

HANNAH: Hay cosas en la educación de las que piensa que necesita más información o hay temas que siente le falta información?

MUJER: Pues no

HANNAH: Siente que tiene suficiente información?

MUJER: Ohm, si

Title: Los Leches-1 (1).docx

Descriptor Info: Age: Interview Location: Residence: Interviewer: Date of Interview:

Codes Applied: Sexual education

Excerpt Package: 1190

Excerpt End: 1500

Sara: Tell me your experience about sex education and sexual health? Have you received sex education?

Woman: No, I haven’t.

Sara: None of the clinics guys or in the school gave you that type of education?

Woman: Neither. I was not at school my parents did not put me to school I grew up working in the orchard.

Title: Santa Ana-3.docx

Descriptor Info: Age: Interview Location: Residence: Interviewer: Date of Interview:

Codes Applied: Sexual education

Excerpt Package: 2901

Excerpt End: 4008

MUJER: Bueno cuando mi tía sufrió esa enfermedad del cáncer de cuello uterino, ella era tan hermosa y después se puso tan delgadita, le hicieron quimioterapias, pero ella ya se lo detectaron muy avanzado ya que ella no acostumbraba a hacerse el pap entonces se lo diagnosticaron ya muy avanzado, las quimioterapias ya no le ayudaban porque el cáncer ya estaba en un término entonces se combatió pero no se curó y son muy dolorosos los tratamientos.

HANNAH: Recibió solo quimioterapia o también una estiractomia o solo las quimioterapias?

MUJER: Solo las quimioterapias.

HANNAH: Oh lo siento, cuantos años tenía ella cuando paso por esto?

MUJER: Ella tenía treinta años

HANNAH: Oh lo siento mucho, y hace cuantos años fue su muerte?

MUJER: Tiene once años de muerta

HANNAH: Después de su muerte hablan más acerca de esta enfermedad con su familia? Como le afecta esto a su familia?

MUJER: Bueno, nos afecta mucho porque nunca pensamos que alguna de esas enfermedades existía hasta que ya nos pasó y sufrimos esa experiencia fue que supimos que si era cierto lo de esa enfermedad del cáncer de cuello uterino

Title: Perla-4.docx

Descriptor Info: Age: Interview Location: Residence: Interviewer: Date of Interview:

Codes Applied: Sexual education

Excerpt Package: 2104

Excerpt End: 3003

HANNAH: Donde reciben la educación sexual las mujeres?

MUJER: Donde he recibido?

HANNAH: Si

MUJER: Pues yo creo que aquí

HANNAH: Aquí en los centros de salud?

MUJER: Sí, porque a veces dan charlas o el mismo medico orienta al paciente

HANNAH: Piensa que usted tiene suficiente información acerca de estos temas?

MUJER: Si yo pienso que sí, ya con tanto tiempo ya ni quiero venir

HANNAH: Y si quiere recibir más información donde puede recibirla?

MUJER: Pues la verdad que no, pero como siempre estoy en constante consulta con el ginecólogo a él es quien le hago las preguntas o con los doctores del centro hablo de cualquier inconveniente que se presente en mi

HANNAH: Cree que las chicas adolescentes tienen suficiente información acerca de su salud sexual?

MUJER: Pues si yo pienso que ahora en los colegios se maneja mas eso

HANNAH: La educación en los colegios es suficiente?

MUJER: Si, así es

Title: Santa Ana-3.docx

Descriptor Info: Age: Interview Location: Residence: Interviewer: Date of Interview:

Codes Applied: Sexual education

Excerpt Package: 5548

Excerpt End: 6128

HANNAH: Y cómo piensa usted que es la actitud y conocimiento de las mujeres en la comunidad acerca de esta enfermedad?

MUJER: Bueno hoy en día veo que son más dóciles porque ya se están yendo a hacer más las revisiones sobre el cáncer de mama, uterino y otras enfermedades más, ahora es más abierto porque ponen unos rótulos y eso es muy bueno porque así leen y así ellas están prestando atención cuando están las charlas o cuando están en consulta la doctora les pregunto o ellas a la doctora y eso es lo bueno, aunque ellas no anden eso pero la doctora siempre está insistiendo.

Title: Perla-3.docx

Descriptor Info: Age: Interview Location: Residence: Interviewer: Date of Interview:

Codes Applied: Sexual education

Excerpt Package: 13379

Excerpt End: 13822

HANNAH: Y tiene hijas usted?

MUJER: Si tengo una niña de tres años.

HANNAH: Oh bueno y piensa que usted va a hablar con su hija acerca de estos temas se sentiría bien?

MUJER: Ah claro, igual con mis hijos varones que yo tengo, uno de diecisiete años y uno de diez y yo así les digo, que tienen que evitar las enfermedades porque además de que el medicamento es caro también la vida propia corre riesgo y que tienen que tener responsabilidades.

Title: Perla-3.docx

Descriptor Info: Age: Interview Location: Residence: Interviewer: Date of Interview:

Codes Applied: Sexual education

Excerpt Package: 13823

Excerpt End: 13992

HANNAH: Que hace usted para mantener la salud sexual?

MUJER: Que hago? Pues venirme a estar chequeando, hacerme mi chequeo médico para ver porque realmente es necesario.

Title: Perla-3.docx

Descriptor Info: Age: Interview Location: Residence: Interviewer: Date of Interview:

Codes Applied: Sexual education

Excerpt Package: 13379

Excerpt End: 13822

HANNAH: Y tiene hijas usted?

MUJER: Si tengo una niña de tres años.

HANNAH: Oh bueno y piensa que usted va a hablar con su hija acerca de estos temas se sentiría bien?

MUJER: Ah claro, igual con mis hijos varones que yo tengo, uno de diecisiete años y uno de diez y yo así les digo, que tienen que evitar las enfermedades porque además de que el medicamento es caro también la vida propia corre riesgo y que tienen que tener responsabilidades.

Title: Mantica-2.docx

Descriptor Info: Age: Interview Location: Residence: Interviewer: Date of Interview:

Codes Applied: Sexual education

Excerpt Package: 1023

Excerpt End: 1880

ALEXANDRA: Y también pueden recibir educación sexual aquí en el centro de salud

MUJER: Si porque también te dan charlas

ALEXANDRA: También ha recibido educación sexual en su colegio

MUJER: En los colegios no mejor dicho no sé por qué yo soy originario de Estelí

ALEXANDRA: Originaria de dónde?

MUJER: De Estelí soy nueva aquí tengo no mas como dos años de vivir aquí pero creo que si porque me han contado que si se da esa educación

ALEXANDRA: Cree que hay suficiente información para las chicas adolescentes

MUJER: Aah!

ALEXANDRA: Cree que las chicas adolescentes son bastante educadas para tomar decisiones de su salud sexual

MUJER: Yo digo que sí al menos que ellos no entiendan porque ahora en los colegios hablan den todos eso de cómo cuidarse, se habla de la salud de la higiene y todo eso

ALEXANDRA: Es más abierto

MUJER: Si es más abierto de hablar

Title: Mantica-2.docx

Descriptor Info: Age: Interview Location: Residence: Interviewer: Date of Interview:

Codes Applied: Sexual education

Excerpt Package: 9031

Excerpt End: 9595

ALEXANDRA: Tiene algo más que decir acerca de la salud de las mujeres o acerca del cuidado de salud

MUJER: Solo que me gustaría saber de esas enfermedades porque son importantes y me gustaría saber mas

ALEXANDRA: Usted piensa que tiene suficiente información para tomar decisiones acerca de su salud del porque hacerse un pap o realizarse otros estudios

MUJER: Verdaderamente no, no tengo la suficiente información de que me ayuda o no por eso le digo que me gustaría tener más información porque es importante ms información como del papiloma u otras enfermedades

Title: Mantica-2.docx

Descriptor Info: Age: Interview Location: Residence: Interviewer: Date of Interview:

Codes Applied: Sexual education

Excerpt Package: 7018

Excerpt End: 8017

ALEXANDRA: Y de donde prefiere recibir información acerca de su salud de los médicos o de cuales medios

MUJER: De los médicos porque son los que saben y son puestos por Dios y si alguien más sabe que de información

ALEXANDRA: Y tiene mucha confianza en lo que digan los médicos

MUJER: En algunos si porque a veces los doctores no se, por ejemplo en mi caso yo tengo cinco meses de que perdí un bebe y me confié porque el doctor me decía que estaba bien él bebe, porque yo le decía que no se movía y él me dijo que todo estaba bien pero a los ocho días otra doctora me miro y me dijo que él bebe estaba muerto entonces algunos saben y lo dicen todo pero otros no, pero eso me paso

ALEXANDRA: Y con otros exámenes ´por ejemplo la prueba de pap tiene confianza en que da información precisa acerca de su salud

MUJER: Yo dos veces me he hecho el pap y me sale lo que debe de ser, algunas dicen que en privado es hacerse mejor la prueba de pap pero yo digo que es igual porque todos pasan por el hospital

Title: Perla-5.docx

Descriptor Info: Age: Interview Location: Residence: Interviewer: Date of Interview:

Codes Applied: Sexual education

Excerpt Package: 13269

Excerpt End: 13980

ALEXA: Cuales son sus preocupaciones más grandes acerca de su salud y salud sexual?

MUJER: Bueno, siempre hay que tener cuidado, preocupaciones siempre van a haber porque no sabemos a lo largo del tiempo y mañana no se sabe, entonces considero que siempre debo estarme chequeando porque uno nunca sabe que un quiste y puede ser un cáncer, entonces uno nunca sabe y por eso siempre debe estar pendiente con la situación.

ALEXA: Y para protegerse así mismo que precauciones toma con su salud?

MUJER: Bueno cuando estoy en tratamiento no tengo relaciones sexuales, dos cuando salgo del tratamiento lo que me recomiendan es usar condón con mi pareja pero por lo general nosotros no usamos por lo que estamos juntos.

Title: Los Leches-2.docx

Descriptor Info: Age: Interview Location: Residence: Interviewer: Date of Interview:

Codes Applied: Sexual education

Excerpt Package: 424

Excerpt End: 1251

HANNAH: Dígame acerca de su experiencia sobre la salud sexual y la educación sexual?

MUJER: Sobre la sexualidad?

HANNAH: Si, acerca de la educación de la salud sexual?

MUJER: Bueno ahí no sé yo, con lo mío, he estado bien porque no he tenido problemas con mi marido, pero con las demás parejas no se ahí si no puedo explicar

HANNAH: Y ha recibido educación sexual de un lugar?

MUJER: Lo he recibido del centro de salud las enfermedades como se transmites y todo

HANNAH: Piensa que tiene suficiente información acerca de estos temas o quiere recibir más?

MUJER: No, creo que no porque ya me han explicado bastante

HANNAH: Y como podría mejorar el conocimiento de las chicas adolescentes e las chicas acerca de las salud sexual?

MUJER: Pues les diría que se cuiden por que como están las enfermedades actualmente es algo duro eso

Title: Santa Ana-2.docx

Descriptor Info: Age: Interview Location: Residence: Interviewer: Date of Interview:

Codes Applied: Sexual education Pap smear

Excerpt Package: 8059

Excerpt End: 8480

ALEXANDRA: Y por cuales medios prefiere recibir información acerca de su salud?

MUJER: Bueno yo digo que por lo menos en los centros de salud

ALEXANDRA: Y hay algo mas que quiera compartir acerca de la salud de las mujeres?

MUJER: Bueno pues solamente que nos debemos cuidar todas que nos hagamos todos nuestros exámenes como el Papanicolaou porque así nos podemos dar cuenta de todas las enfermedades que podemos tener.

Title: Perla-2.docx

Descriptor Info: Age: Interview Location: Residence: Interviewer: Date of Interview:

Codes Applied: Sexual education Pap smear

Excerpt Package: 7289

Excerpt End: 7870

ALEXANDRA: Cree usted que todas las chicas adolescentes deben tener una prueba de pap si tienen relaciones sexuales?

MUJER: Si

ALEXANDRA: Hay otras cosas que ellas deben de hacer para protegerse?

MUJER: Si, usar el condón

ALEXANDRA: Cree que hay una relación entre la prueba y la promiscuidad sexual? Por ejemplo si una chica se hace la prueba de pap será que una chica sea más propensa a tener relaciones sexuales?

MUJER: Pues ellas se sienten seguras que no tienen nada pero deben de cuidarse y con las relaciones sexuales se transmiten eso y no estará segura si no usa el condón

Title: Poneloya-1.docx

Descriptor Info: Age: Interview Location: Residence: Interviewer: Date of Interview:

Codes Applied: Sexual education Pap smear Promiscuity

Excerpt Package: 6459

Excerpt End: 7147

ALEXANDRA: Y que preocupaciones toma usted para prevenir estas enfermedades?

MUJER: Bueno lo que yo pienso es que si llego a tener relaciones sexuales con una persona que es solo mi novio y no mi esposo, usaría el condón, pero ya cuando una persona vive con ese alguien o es su esposo, es ilógico usar métodos pero a la vez es bueno hacerlo para prevenir porque uno no sabe si el hombre anda con otras y por eso es bueno el Papanicolaou el pap que le llaman para prevenir alguna enfermedad sexual, por ejemplo yo si me hiciera eso y tuviera una enfermedad sexual y solo estoy con mi marido, analizaría por qué y de donde trajo esa enfermedad, entonces ahí uno tiene que pensar al respecto

Title: Poneloya-1.docx

Descriptor Info: Age: Interview Location: Residence: Interviewer: Date of Interview:

Codes Applied: Sexual education Private/taboo

Excerpt Package: 5939

Excerpt End: 6458

ALEXANDRA: Y de dónde prefiere usted recibir información acerca de su salud?

MUJER: De mi salud? Como? Charlas sobre la sexualidad? Bueno es que en realidad yo sé que uno no siempre está bien informado de todo, pero ya sabemos cosas, y normalmente nuestros padres nos hablan al respecto, nos aconsejan pero no nos dicen todo por lo mismo ya que les da vergüenza, en las charlas que nos dan no nos dicen las cosas directamente, por ejemplo yo no hablaría de eso con una amiga sino que mejor me informo o aplico lo que se

Title: Los Leches-1 (1).docx

Descriptor Info: Age: Interview Location: Residence: Interviewer: Date of Interview:

Codes Applied: Sexual education Private/taboo

Excerpt Package: 7152

Excerpt End: 7587

Sara: How do you take decisions?...Ups, Sorry, I mean, are you going to talk about this with your sons and daughters about these health issues?

Woman: What do you mean? If I ever talk to them about it or if I will talk to them later?

Sara: If you talk about sexual diseases

Woman: No, no, no, I do not think so.

Sara: But you will talk about problems with the pressure, heart or something like that?

Woman: Yes, about that absolutely.

Title: Mantica-4.docx

Descriptor Info: Age: Interview Location: Residence: Interviewer: Date of Interview:

Codes Applied: Sexual education Private/taboo

Excerpt Package: 4793

Excerpt End: 5071

HANNAH: Piensa que en un futuro va a hablar con sus hijos sobre su experiencia y sobre la salud sexual?

MUJER: Si, por lo menos para que ellos sepan lo que en el futuro les espera y así tener confianza con los hijos y poder platicar abiertamente con ellos para que sepan de todo

**Support**

Title: Perla-3.docx

Descriptor Info: Age: Interview Location: Residence: Interviewer: Date of Interview:

Codes Applied: Support

Excerpt Package: 11039

Excerpt End: 11376

HANNAH: Y con quien hablo acerca de su enfermedad? Es decir ella hablo mucho con su familia o con amigos?

MUJER: No, solo con nosotros sus hijas

HANNAH: Solo sus hijas?

MUJER: Si solamente sus hijas con nadie más, después de eso entonces se sintió en el deber y derecho de comentárselo a una hermana de ella que trabajaba en el hospital.

Title: Mantica-1.docx

Descriptor Info: Age: Interview Location: Residence: Interviewer: Date of Interview:

Codes Applied: Support

Excerpt Package: 7721

Excerpt End: 8684

Sara: Como toma decisiones con respecto a su salud habla con su pareja médica o familia?

Mujer: Yo no tengo pareja soy sola si pero cuando voy al centro yo pregunto cosas antes iba a un proyecto que se llama Mery barreda y mucho me decían sobre eso de las relaciones y todo eso, pero era cuando estaba pequeña así que ya no me acuerdo casi de nada pero nos decían que nos cuidáramos y protegiéramos y que visitáramos los centros que cuando tuviéramos nuestra primera pareja preguntáramos pero ya casi no me acuerdo.

Sara: Que hace para mantener la salud y seguridad contra enfermedades de transmisión sexual?

Mujer: Que hago yo? Pues voy al centro de salud y me hago el pap, pero como actualmente no tengo pareja y así estoy mejor y a como son los hombres ahora con quien estuvieron o no y yo tengo como que miedo, y todo eso es lo único que se, y como ya no visito ese proyecto al que iba ya solo se lo que dicen en el centro de salud y veo los carteles que hay

Title: Subtiava-1.docx

Descriptor Info: Age: Interview Location: Residence: Interviewer: Date of Interview:

Codes Applied: Support

Excerpt Package: 16691

Excerpt End: 17200

HANNAH: Hay algunas fundaciones u organizaciones que apoyan a las mujeres con esas enfermedades?

MUJER: La verdad yo creo que si hay, pienso que sí, yo he oído comentarios de que si hay pero a veces uno no tiene comunicación para contactarse y poder recibir ese apoyo.

HANNAH: Y hay grupos de apoyo de otras mujeres con cáncer?

MUJER: Si, también

HANNAH: En el hospital?

MUJER: Si, en el hospital y los centros de salud.

HANNAH: Y su amiga o su hermana han recibido apoyo de estos grupos?

MUJER: No, ellas no.

Title: Perla-3.docx

Descriptor Info: Age: Interview Location: Residence: Interviewer: Date of Interview:

Codes Applied: Support

Excerpt Package: 13993

Excerpt End: 14730

HANNAH: Y como toma decisiones usted acerca de su salud?

MUJER: Como tomo decisiones? Bueno la decisión que yo tomo es que siempre y cuando sea conveniente a mi salud porque todo tiene que ser positivo para uno poder cuidarse, poder tener una buena salud debe de ser positiva y no decir, no iré al centro de salud porque ahí no me dan nada que solo dan suero y acetaminofén, muchas veces he pensado así yo pero en realidad es necesario porque quizás los médicos tienen un poco más de experiencia que nosotros los que estamos en casa que no sabemos nada de medicina.

HANNAH: Y consulta con alguna persona antes de tomar decisiones o no?

MUJER: Pues en salud no le pido ni decisión ni opinión a nadie ni en mi salud ni en la de mis hijos.

Title: Mantica-2.docx

Descriptor Info: Age: Interview Location: Residence: Interviewer: Date of Interview:

Codes Applied: Support

Excerpt Package: 5890

Excerpt End: 7017

ALEXANDRA: Como toma decisiones usted acerca de su salud en general y su salud sexual

MUJER: Como en que partes

ALEXANDRA: Hace decisiones en conjunto con otras personas familiares o su pareja

MUJER: Por lo menos a mí me apoya mi familia y mi marido, por ejemplo en las situaciones en las que una persona se enferma mucho si uno tiene confianza con su familia uno le dice que está pasando y ellos te apoyan, entonces seria con la familia y el esposo

ALEXANDRA: Consultaría con su marido antes de hacerse una prueba de pap

MUJER: Si

ALEXANDRA: Pero su pareja no tendría ningún problema con si se hace la prueba

MUJER: Como no le entiendo

ALEXANDRA: Es que si tendría algún problema con si usted se realiza una prueba de pap

MUJER: Y él no se da cuenta

ALEXANDRA: Si se da cuenta

MUJER: Pero se da cuenta después

ALEXANDRA: Si, algo así

MUJER: No, no se pone mal, porque eso es algo que uno como mujer se debe de hacer incluso él ha venido aquí a hacerse exámenes y cosas así

ALEXANDRA: Se toman en cuenta sus creencias religiosas con respecto al tomar decisiones acerca de su salud?

MUJER: Uhm, no por que eso es algo personal

Title: Mantica-4.docx

Descriptor Info: Age: Interview Location: Residence: Interviewer: Date of Interview:

Codes Applied: Support

Excerpt Package: 3204

Excerpt End: 3920

HANNAH: Y sabe usted de otras enfermedades que han sufrido del cáncer de cuello uterino?

MUJER: Pues en el hospital me he encontrado con personas que también están sufriendo mi enfermedad y así como ellas lo han superado espero yo también porque mi Dios me esta ayudando muchísimo

HANNAH: Es en forma de grupos e apoyo o es solo un grupo en el hospital de otros pacientes?

MUJER: Sí, es un grupo en el hospital de otros con quienes he hecho amistad porque están viviendo la misma situación

HANNAH: Y piensa que ellos también tienen su situación de la enfermedad como algo privado o tienen maneras diferentes de compartir su experiencia?

MUJER: Bueno cada quien tiene su forma de ser y sabe a quien contarle sus cosas

Title: Perla-3.docx

Descriptor Info: Age: Interview Location: Residence: Interviewer: Date of Interview:

Codes Applied: Support

Excerpt Package: 12604

Excerpt End: 12799

HANNAH: Y su madre recibió apoyo de algún grupo del hospital de mujeres cáncer o cómo?

MUJER: Si de todo eso, porque ella anda buscando ayuda pero solo de ayuda médica pero con otras mujeres no.

Title: Mantica-4.docx

Descriptor Info: Age: Interview Location: Residence: Interviewer: Date of Interview:

Codes Applied: Support

Excerpt Package: 6775

Excerpt End: 6952

HANNAH: Recibe información de cada parte que necesita? Un buen apoyo?

MUJER: Si muy buena información y estoy agradecida con el hospital porque está haciendo muy bien su trabajo

Title: Mantica-6.docx

Descriptor Info: Age: Interview Location: Residence: Interviewer: Date of Interview:

Codes Applied: Support

Excerpt Package: 7027

Excerpt End: 7807

ALEXANDRA: Y como toma decisiones usted acerca de su salud y salud sexual junto con alguien o solo con los médicos?

MUJER: Junto con mi pareja y con los médicos principalmente

ALEXANDRA: Y consultaría con su pareja antes de hacerse una prueba de pap?

MUJER: No, porque es algo individual esa es mi preocupación mi salud y mi cuerpo pero que si sepa que lo estoy haciendo porque si el anda para que no me haga el daño y que si yo me estoy chequeando él también tiene que chequearse si estamos juntos sino pues igual debo hacerme mis chequeos de mama como igual que toda mujer porque todo eso nos atrae enfermedades y nos puede llevar a la muerte

ALEXANDRA: Y las razones religiosas influyen en alguna parte de su vida o en su vida sexual o de salud?

MUJER: No, no influye en nada

Title: Mantica-2.docx

Descriptor Info: Age: Interview Location: Residence: Interviewer: Date of Interview:

Codes Applied: Support

Excerpt Package: 3515

Excerpt End: 3675

ALEXANDRA: Se sentiría cómoda de hablar del cáncer cervical o algo como eso con un médico varón

MUJER: No tanto, sería mejor con una doctora, familia y mi madre

Title: Perla-5.docx

Descriptor Info: Age: Interview Location: Residence: Interviewer: Date of Interview:

Codes Applied: Support

Excerpt Package: 10575

Excerpt End: 11862

ALEXA: Y las creencias religiosas influyen en la toma de decisiones de su salud?

MUJER: Mi religión? Bueno yo soy católica pero eso no impide que yo me haga mis exámenes porque tenemos que pasar por ciertos regímenes de salud y no es algo que me lo restrinja mi religión es algo personal, pues son cosas de salud, salud y religión son cosas importantes, pero no me pasa que tal vez mi religión me diga que no me puedo hacer una transfusión de sangre porque mi religión no me lo permita eso es malo, eso es pecado, es de otra persona, pero eso es malo porque si el ya necesitaba la transfusión de sangre, porque si el necesita la sangre es de urgencia y emergencia creo que eso mi religión no debería tomar la decisión por mí, pero a veces no lo hacen porque se toman la religión muy a pecho.

ALEXA: No hacen las transfusiones de sangre?

MUJER: No, es que hay una religión que hace eso, lo vivió mi mama porque ella trabajo en el hospital en la sala de UCI, CUIDADOS INTENSIVOS NEONATO, en ocasiones los niños necesitan transfusión de sangre pero no lo hacen porque la religión no se lo permite, pero una vida que acaba de nacer y dejarlo morir ahí solo, son decisiones fuertes y a veces tal vez ella no se hubiera quedado callada y no hubiera dejado que tomaran las decisiones por ellos.

Title: Mantica-3.docx

Descriptor Info: Age: Interview Location: Residence: Interviewer: Date of Interview:

Codes Applied: Support

Excerpt Package: 5188

Excerpt End: 5572

HANNAH: Con quien se sentiría cómoda hablar acerca del cáncer de cuello uterino y virus de papiloma humano con amigos, familia?

MUJER: Bueno, con mi compañero porque es con quien tengo más confianza ya que no tengo ni papa ni mama, es con el único que converso sobre esas cosas

HANNAH: Y los médicos?

MUJER: Aquí también cunado vengo a pasar consulta les pregunto para que me indiquen

Title: Los Leches-2.docx

Descriptor Info: Age: Interview Location: Residence: Interviewer: Date of Interview:

Codes Applied: Support

Excerpt Package: 4742

Excerpt End: 4911

HANNAH: Como hace para tomar decisiones acerca de su salud? Le consulta a alguien un médico, habla con su pareja?

MUJER: Yo hablo con mi mama, con mi pareja y la doctora

Title: Santa Ana-1.docx

Descriptor Info: Age: Interview Location: Residence: Interviewer: Date of Interview:

Codes Applied: Support

Excerpt Package: 5491

Excerpt End: 5853

SARA: Como toma decisiones acerca de su salud, consulta con su familia, medica, pareja?

MUJER: Si con todos ellos igual

SARA: Que hace para mantener la salud y seguridad de la salud contra enfermedades de transmisión sexual

MUJER: Pues a como le digo tomando las medidas preventivas y siguiendo los consejos de los doctores, usando protección y todas esas cosas.

Title: Perla-2.docx

Descriptor Info: Age: Interview Location: Residence: Interviewer: Date of Interview:

Codes Applied: Support

Excerpt Package: 5441

Excerpt End: 6147

ALEXANDRA: Oh wow, entonces no había otra opción para ella, entonces si usted contrae el cáncer cervical que decisiones tomaría?

MUJER: Pues que me saquen la matriz para vivir un poco más por mis hijos

ALEXANDRA: Y eso usted cree que su pareja tendría un problema con eso?

MUJER: No eso es decisión mía porque es mi salud yo soy la que quiero vivir un poco mas

ALEXANDRA: Y con las pruebas de pap? Consultaría con su pareja o es algo que decidiría por si misma?

MUJER: Si, consulto con el también

ALEXANDRA: Pero tendría un problema o no?

MUJER: No.

ALEXANDRA: Como influyen sus creencias religiosas en sus decisiones de salud? O como es su influencia?

MUJER: Si, solo rogándole a Dios que todo salga bien

Title: Santa Ana-2.docx

Descriptor Info: Age: Interview Location: Residence: Interviewer: Date of Interview:

Codes Applied: Support

Excerpt Package: 7871

Excerpt End: 8058

ALEXANDRA: Que si algunas razones religiosas influyen en salud?

MUJER: Que si hay alguna religión? Sí, soy evangélica.

ALEXANDRA: Y cómo influye en su salud?

MUJER: Pues Gracias a Dios no

Title: SantaAna-4.docx.docx

Descriptor Info: Age: Interview Location: Residence: Interviewer: Date of Interview:

Codes Applied: Support

Excerpt Package: 7078

Excerpt End: 7553

ALEXANDRA: Si usted contrae el cáncer de cuello uterino por ejemplo con quien se sentiría cómoda hablar acerca de su enfermedad?

MUJER: Bueno yo hablaría y tendría que someterme a la cosa de Dios porque solamente él puede curar de cualquier enfermedad

ALEXANDRA: Entonces le informaría algo a sus hijos?

MUJER: Si se lo diría a mis hijos, a mi marido y a mi familia

ALEXANDRA: Como toma decisiones usted acerca de su salud?

MUJER: Bueno yo diría con mi familia las tomaría

Title: Los Leches-3.docx

Descriptor Info: Age: Interview Location: Residence: Interviewer: Date of Interview:

Codes Applied: Support

Excerpt Package: 3945

Excerpt End: 4425

SARA: Ha oído de cáncer de cuello uterino sabe de alguien que tiene esa enfermedad?

MUJER: Mi prima pero es mujer

SARA: Oh y cuál fue su experiencia que ocurrió?

MUJER: Pues la verdad aquí fue la doctora la que se lo descubrió, con el examen del pap que le miraron algo raro y hasta la fecha ella se está chequeando pero ella se ha visto bien mal

SARA: Fue al hospital por tratamiento?

MUJER: Si, si fue

SARA: Y ella habla con su familia y su pareja sobre la enfermedad?

MUJER: Si

Title: Santa Ana-2.docx

Descriptor Info: Age: Interview Location: Residence: Interviewer: Date of Interview:

Codes Applied: Support

Excerpt Package: 7655

Excerpt End: 7775

ALEXANDRA: Y como toma decisiones acerca de su salud

MUJER: Bueno, yo las tomo junto con mi marido, junto con mi pareja

Title: Poneloya-2.docx

Descriptor Info: Age: Interview Location: Residence: Interviewer: Date of Interview:

Codes Applied: Support

Excerpt Package: 9457

Excerpt End: 12238

ALEXA: Y cuando piensa en cáncer de cuello uterino en que piensa usted?

MUJER: Lo mismo que vuelvo y repito que somos nosotros los que debemos cuidarnos y detectarnos eso que tenemos porque si yo ya tengo un poco que me va a salir entonces tendría posibilidades para que el cáncer no siga por eso es que ahí se inicia detectar con el pap, es que el pap es lo más esencial para nosotras las mujeres porque ahí donde nos detectan las enfermedades que tenemos, yo puedo tener cuatro o cinco años de no tener relaciones sexuales pero yo siempre tengo que hacerme el pap porque es algo que uno anda dentro oculto si tuve alguna inflamación, o si la menstruación me dilataba bastante en bajar, nueve días el sangrado uno tiene que verse el porqué de todas esas situaciones, así le dije yo hace cinco años a una que era mi patrona, ella tuvo cuatro hijos, cuando su primer embarazo cuando ella estaba joven no había tenido hijos ni nada a ella le venía su menstruación demasiado fuerte y tal vez ella estaba trabajando en la oficina y cuando se miraba estaba manchada atrás de la abundancia de sangre que a ella le salía, después ella salió con su primer embarazo y fueron gemelos, tuvo los niños y aquella abundancia de sangrado que ella tuvo, yo le decía a ella porque era mi patrona yo la estimaba mucho y teníamos confianza yo le dije que se fuera a ver dónde una ginecóloga y que le dijera su problema porque ese sangrado es malo, por lo general nosotras las mujeres nuestra menstruación nos puede durar cuatro o cinco días pero ya es demasiado y es malo, ella fue y después volvió a salir embarazada y ya ahí el cabello se le caía lo dejaba en la almohada entonces después tuvo el otro parto y yo le decía que me hiciera caso y fuera, al tiempo yo deje de trabajar ahí y lo que ella tenía era cáncer y al poco tiempo se estaba muriendo, le sacaron todo, y era una mujer joven, después yo fui porque me mandaron a llamar para que la curara por la herida que le hicieron y yo le dije ve que era algo lo que tenía, ahora que está un poco mejor pero ya sabe que lo que tiene cáncer, se le cayó el cabello y esta delgada.

ALEXA: Ella sobrevivió?

MUJER: Si, ahí está, delgada pero ahí está, pero le sacaron todo.

ALEXA: Y ella le dijo a sus hijos que tenía cáncer?

MUJER: Si, ella le dijo a sus niños y a su esposo, ya ahora sus niños son adolescentes y ella ahí está pero ella ya sabe lo que tiene y yo se lo decía a ella que tuviera cuidado por eso mismo.

ALEXA: Porque piensa que ella espero tanto tiempo?

MUJER: Porque tal vez ella pensó que era mentira lo que le decía, porque como yo no soy médico y yo le decía porque a nosotros nos daban charlas los médicos y ellos nos dicen de donde proviene el cáncer y cuáles son sus síntomas, entonces por eso yo le decía que fuera al médico.

Title: Poneloya-2.docx

Descriptor Info: Age: Interview Location: Residence: Interviewer: Date of Interview:

Codes Applied: Support

Excerpt Package: 12239

Excerpt End: 14022

ALEXA: Como toma decisiones usted acerca de su salud?

MUJER: Yo de mi salud vivo pendiente, por ejemplo ayer me dijeron venga a hacerse el pap y yo me decidí a venir, para ellas no es importante, ellas se lo hacen pero yo soy la que tengo que ver por mi salud no las enfermeras ni los médicos porque ellos se chequean con sus médicos, hacen mucho con recordarle a uno lo que debe de hacer por ejemplo recordar a que se vengan a hacer el pap o si alguien padece de la presión decirle que venga a chequeársela y llevarse sus medicamentos porque le que debe de preocuparse por su salud es uno, no el medico porque soy yo la que tengo la enfermedad pero aquí hacemos muchas personas que no somos así que en mi concepto cada quien debe revisarse a como le digo con los médicos y si es fin de semana voy donde otra doctora aparte que también le tengo confianza .

ALEXA: Y toma decisiones con alguien acerca de su salud con su pareja?

MUJER: No tengo pareja, solo con mis hijas, y si ellas me ven con algo ya ellas me dicen que vaya donde el médico, mírate tu salud, mira que ya vas mayor todo se viene disparando la presión, el ácido úrico, etc., porque a todos tantos mujeres como varones todo se nos dispara pero soy yo la que me tengo que preocupar y mis hijos porque si ellos me quieren seguir teniendo ahí deben hacerlo, yo tengo a mis hijos que me apoyan, mi hijo varón me dice, anda madre, anda donde el médico, yo te miro la piel extraña, anda a revisarte, soy yo la que tengo que ver mi salud no nadie más.

ALEXA: Y la religión influye en sus decisiones de salud?

MUJER: En mi concepto si yo me siento algo yo le pido a mi Dios, sola en mi cama en mi cuarto y eso es todo y si yo quiero visitar una iglesia pero solo si yo quiero, sino no voy, pero que alguien me va a obligar no.

Title: Subtiava-1.docx

Descriptor Info: Age: Interview Location: Residence: Interviewer: Date of Interview:

Codes Applied: Support

Excerpt Package: 15304

Excerpt End: 15580

HANNAH: Piensa que es más difícil para su hermana y su amiga luchar contra las enfermedades sin el apoyo de muchas personas?

MUJER: A veces uno necesita apoyo también comunicación porque eso ayuda, claro también necesita del médico, también del Señor pero también de la ayuda.

Title: Perla-3.docx

Descriptor Info: Age: Interview Location: Residence: Interviewer: Date of Interview:

Codes Applied: Support

Excerpt Package: 13993

Excerpt End: 14730

HANNAH: Y como toma decisiones usted acerca de su salud?

MUJER: Como tomo decisiones? Bueno la decisión que yo tomo es que siempre y cuando sea conveniente a mi salud porque todo tiene que ser positivo para uno poder cuidarse, poder tener una buena salud debe de ser positiva y no decir, no iré al centro de salud porque ahí no me dan nada que solo dan suero y acetaminofén, muchas veces he pensado así yo pero en realidad es necesario porque quizás los médicos tienen un poco más de experiencia que nosotros los que estamos en casa que no sabemos nada de medicina.

HANNAH: Y consulta con alguna persona antes de tomar decisiones o no?

MUJER: Pues en salud no le pido ni decisión ni opinión a nadie ni en mi salud ni en la de mis hijos.

Title: Perla-3.docx

Descriptor Info: Age: Interview Location: Residence: Interviewer: Date of Interview:

Codes Applied: Support

Excerpt Package: 12604

Excerpt End: 12799

HANNAH: Y su madre recibió apoyo de algún grupo del hospital de mujeres cáncer o cómo?

MUJER: Si de todo eso, porque ella anda buscando ayuda pero solo de ayuda médica pero con otras mujeres no.

Title: Perla-3.docx

Descriptor Info: Age: Interview Location: Residence: Interviewer: Date of Interview:

Codes Applied: Support

Excerpt Package: 11039

Excerpt End: 11376

HANNAH: Y con quien hablo acerca de su enfermedad? Es decir ella hablo mucho con su familia o con amigos?

MUJER: No, solo con nosotros sus hijas

HANNAH: Solo sus hijas?

MUJER: Si solamente sus hijas con nadie más, después de eso entonces se sintió en el deber y derecho de comentárselo a una hermana de ella que trabajaba en el hospital.

Title: Santa Ana-3.docx

Descriptor Info: Age: Interview Location: Residence: Interviewer: Date of Interview:

Codes Applied: Support

Excerpt Package: 5205

Excerpt End: 5547

HANNAH: Y usted sabe de algunos grupos de apoyo para las mujeres con cáncer de cuello uterino o no es común eso?

MUJER: Hay porque hay fundaciones, hay bastantes grupos confirman un grupo las enfermeras y ellas salen a buscar con los resultados a las mujeres y les explican y la hacen llegar para que reciban el tratamiento y se ´puedan curar

Title: Perla-4.docx

Descriptor Info: Age: Interview Location: Residence: Interviewer: Date of Interview:

Codes Applied: Support

Excerpt Package: 7642

Excerpt End: 7814

HANNAH: Como toma decisiones acerca de la salud sexual? Habla con una persona acerca de eso?

MUJER: Pues no, casi no

HANNAH: Es una cosa independiente para usted?

MUJER: Si

Title: Los Leches-3.docx

Descriptor Info: Age: Interview Location: Residence: Interviewer: Date of Interview:

Codes Applied: Support

Excerpt Package: 5188

Excerpt End: 5348

SARA: Como toma decisiones acerca de su salud, consulta con su pareja o familia?

MUJER: Bueno, si estoy con mi mama, con ella, si estoy con mi pareja es con el

Title: Perla-4.docx

Descriptor Info: Age: Interview Location: Residence: Interviewer: Date of Interview:

Codes Applied: Support

Excerpt Package: 6032

Excerpt End: 6445

HANNAH: Como le afecta a su vida?

MUJER: La verdad que a veces yo no quiero porque me van a decir algo y tal vez me van a acelerar el poco tiempo que tengo de vida cuando uno se da cuenta de una enfermedad como que se aflige mas

HANNAH: Ahora usted viene cada seis meses?

MUJER: Si tengo que estar viniendo porque el doctor dice que aún no me puede dar de baja que debo de seguir en chequeos así que aquí estamos.

Title: Mantica-5.docx

Descriptor Info: Age: Interview Location: Residence: Interviewer: Date of Interview:

Codes Applied: Support

Excerpt Package: 4942

Excerpt End: 5445

HANNAH: Como toma decisiones sobre su salud, habla con su pareja, con su familia o es algo independiente?

MUJER: Bueno yo pienso que es cosa de cada quien porque si yo le digo, mira iré hoy al centro de salud y él dice no, no vayas hoy ve mañana eso es algo que cada quien lo siente, y eso es algo de cada uno porque debe ir y es su decisión porque es su cuerpo es su salud individual.

HANNAH: Se siente cómoda hablando con las enfermeras y médicos sobre el VPH y el cáncer de cuello uterino?

MUJER: Si

Title: Los Leches-1 (1).docx

Descriptor Info: Age: Interview Location: Residence: Interviewer: Date of Interview:

Codes Applied: Support

Excerpt Package: 8278

Excerpt End: 8696

Sara: What do you do to maintain the health and safety for your health?

Woman: Well, have patience, have faith in the Lord that is the only one who can help us and heal us, for example now there is a health problem of my son, he does not wants help because he did not want, because we see the things really impossible, but we always ask for help and force to him because if we do not have it we can not go where he is.

Title: Mantica-5.docx

Descriptor Info: Age: Interview Location: Residence: Interviewer: Date of Interview:

Codes Applied: Support

Excerpt Package: 1940

Excerpt End: 2903

HANNAH: usted ha tenido una experiencia con el cáncer de cuello uterino, alguien de su familia o algún amigo que lo haya sufrido?

MUJER: Un conocido, en mi familia Gracias a Dios no, nadie lo ha tenido

HANNAH: Conocido en su familia

MUJER: No, era alguien del Reparto

HANNAH: Ha oído las experiencias de su amiga que ha sufrido de cáncer

MUJER: SI

HANNAH: Como fue su experiencia?

MUJER: Pues fue muy triste porque ella murió a causa de eso, ya que era muy tarde cuando se lo descubrieron, en la Iglesia también a una muchacha le quitaron un pecho y le sacaron y Gracias a Dios ella sobrevivió, pero la que estaba más cerca de mi casa, murió.

HANNAH: Pero ella estaba cómoda hablando acerca de su tratamiento?

MUJER: No porque cuando a ella se lo descubrieron ya no había nada que hacer, todo fue muy tarde.

HANNAH: Y porque piensa que es una cosa muy privada?

MUJER: No sé, pero yo digo que tenemos que hablar para que así estos temas no afecten a otras mujeres

Title: Los Leches-1 (1).docx

Descriptor Info: Age: Interview Location: Residence: Interviewer: Date of Interview:

Codes Applied: Support

Excerpt Package: 7588

Excerpt End: 8277

Sara: And, how do you take decisions about your health? Are you married or do you a have a couple?

Woman: Who? Me? Yes, I have a couple.

Sara: And do you consult with your couple and your family too?

Woman: Yes, of course.

Sara: And, he supports you?

Woman: They supports me and I them, one with another, we are there looking at each other, helping us, because if is not like that the patient will depressed more, looking that is there and that no one turns to see, that’s why we are always looking each other bringing us value and asking force to the Lord, because is the only one who can help us, after him, is the doctor and the treatment, all that is together but first of all is God.

Title: Perla-4.docx

Descriptor Info: Age: Interview Location: Residence: Interviewer: Date of Interview:

Codes Applied: Support

Excerpt Package: 4030

Excerpt End: 4479

HANNAH: Como fue el tratamiento de su amiga?

MUJER: Bueno fue muy duro porque tuvo que viajar hasta Costa Rica y boto el cabello y todo fue muy duro

HANNAH: Ella fue a Costa Rica para recibir tratamiento?

MUJER: Si, unos padres de la Iglesia Católica donde ella trabajaba, ellos le ayudaron de esa manera porque como ella no tenía muchos recursos para viajar entonces a través de una organización que creo es la Ortiz Guardián así ella pudo viajar

Title: Perla-4.docx

Descriptor Info: Age: Interview Location: Residence: Interviewer: Date of Interview:

Codes Applied: Support

Excerpt Package: 2485

Excerpt End: 2766

HANNAH: Y si quiere recibir más información donde puede recibirla?

MUJER: Pues la verdad que no, pero como siempre estoy en constante consulta con el ginecólogo a él es quien le hago las preguntas o con los doctores del centro hablo de cualquier inconveniente que se presente en mi

Title: Subtiava-2.docx

Descriptor Info: Age: Interview Location: Residence: Interviewer: Date of Interview:

Codes Applied: Support

Excerpt Package: 5476

Excerpt End: 5676

HANNAH: Cuál piensa es la actitud acerca del cáncer de cuello uterino?

MUJER: Bueno la verdad es que no se, como no lo he tenido pero creo que si uno le pide ayuda a Dios le ayudara a sanar pero no se

Title: Perla-5.docx

Descriptor Info: Age: Interview Location: Residence: Interviewer: Date of Interview:

Codes Applied: Support

Excerpt Package: 9979

Excerpt End: 10574

ALEXA: Consultaría con su pareja antes de realizarse el pap?

MUJER: Si, yo consulto y esta bien porque es algo que nosotras tenemos interno no externo como ellos, nosotras somos más delicadas en ese aspecto.

ALEXA: Y como toma decisiones acerca de su salud personal? Como por cuales medios reciben información y en conjunto de quien las toma?

MUJER: Bueno me informo cuando tal vez tengo alguna enfermedad, o cuando me siento mal le comunico tanto a él, como a mi mama, a él porque es mi pareja y mi mama porque es mi mama, pero es más con ella porque ella me va atender ya que es mujer también.

Title: Santa Ana-3.docx

Descriptor Info: Age: Interview Location: Residence: Interviewer: Date of Interview:

Codes Applied: Support

Excerpt Package: 2901

Excerpt End: 4440

MUJER: Bueno cuando mi tía sufrió esa enfermedad del cáncer de cuello uterino, ella era tan hermosa y después se puso tan delgadita, le hicieron quimioterapias, pero ella ya se lo detectaron muy avanzado ya que ella no acostumbraba a hacerse el pap entonces se lo diagnosticaron ya muy avanzado, las quimioterapias ya no le ayudaban porque el cáncer ya estaba en un término entonces se combatió pero no se curó y son muy dolorosos los tratamientos.

HANNAH: Recibió solo quimioterapia o también una estiractomia o solo las quimioterapias?

MUJER: Solo las quimioterapias.

HANNAH: Oh lo siento, cuantos años tenía ella cuando paso por esto?

MUJER: Ella tenía treinta años

HANNAH: Oh lo siento mucho, y hace cuantos años fue su muerte?

MUJER: Tiene once años de muerta

HANNAH: Después de su muerte hablan más acerca de esta enfermedad con su familia? Como le afecta esto a su familia?

MUJER: Bueno, nos afecta mucho porque nunca pensamos que alguna de esas enfermedades existía hasta que ya nos pasó y sufrimos esa experiencia fue que supimos que si era cierto lo de esa enfermedad del cáncer de cuello uterino

HANNAH: Durante el tratamiento de su tía piensa de que ella hablo con miembros de su familia o amigos para apoyo?

MUJER: Bueno, nosotros la familia nos dimos cuenta cinco días antes de que ella muriera

HANNAH: Disculpe, ustedes se dieron cuenta hasta cinco días antes?

MUJER: Si, cinco días antes de fallecer supimos que era cáncer

HANNAH: Porque ella no quería hablar?

MUJER: Si, no quería hablar, la única que sabía era mi mama

Title: Santa Ana-3.docx

Descriptor Info: Age: Interview Location: Residence: Interviewer: Date of Interview:

Codes Applied: Support

Excerpt Package: 7571

Excerpt End: 8412

HANNAH: Como usted toma decisiones acerca de su salud?

MUJER: Bueno, mis decisiones sobre mi salud es que si yo me siento mal voy al médico no me auto medico porque eso es malo cualquier inconveniente que yo tengo o cualquier cosa que me sienta mal o que sienta que me pican mis partes íntimas yo corro siempre donde la ginecóloga, o donde la muchacha que hace el control médico que hacen el pap, planificación entonces me voy porque ahí siempre están las licenciadas y las enfermeras también

HANNAH: Y hay una persona con la que le guste consultar acerca de su experiencia con la salud?

MUJER: Hay alguna persona que me guste consultar?

HANNAH: Si como una persona en su vida personal con quien le guste consultar, una familia su pareja?

MUJER: Si, tengo una amiga que me gusta consultarle y ella a mí, cualquier cosa intercambiamos opinión

Title: Subtiava-2.docx

Descriptor Info: Age: Interview Location: Residence: Interviewer: Date of Interview:

Codes Applied: Support

Excerpt Package: 7850

Excerpt End: 8291

HANNAH: Y como toma usted decisiones acerca de su salud?

MUJER: Bueno por lo menos cada vez que me toca hacerme el examen, mi pareja me alienta a chequearme y pienso que me quiere porque si no no lo hiciera y pues ambos a veces juntos nos acompañamos y le tomamos a la vida importancia, el mismo me acompaña a hacerme el pap o cuando traigo a los niños que están enfermos

HANNAH: Consulta con su pareja o su familia?

MUJER: Si, con mi pareja

Title: Mantica-3.docx

Descriptor Info: Age: Interview Location: Residence: Interviewer: Date of Interview:

Codes Applied: Support Physical comfort Confidence

Excerpt Package: 6292

Excerpt End: 6836

HANNAH: Usted consulta con su pareja antes de realizarse una prueba de pap?

MUJER: Eso es una cosa independiente porque es una revisión médica que debe hacerse toda mujer porque ahí te das cuenta si estas enferma si tienes alguna infección para prevenir a tiempo porque si no después son las preocupaciones del porque no se dieron cuenta a tiempo de las enfermedades que tenían porque hay mujeres que no les gusta hacerse eso pero por mi parte yo siempre me hago todos mis exámenes aunque sean dolorosos algunos pero para esta bien debo hacerlo

Title: Perla-3.docx

Descriptor Info: Age: Interview Location: Residence: Interviewer: Date of Interview:

Codes Applied: Support Private/taboo

Excerpt Package: 6644

Excerpt End: 7276

MUJER: No, de eso por el virus porque yo por ejemplo yo tengo una tía y mi tía tiene eso y ella no se recuperaba, esta delgada porque eso te enferma psicológicamente también.

HANNAH: Usted hablo un poquito de su tía, no sé si recuerda.

MUJER: Si, es decir ella no vive conmigo pero vive cerca de mi casa, pero si ella padece eso y ella es promiscua y entonces que le resulto, que le haya pasado eso porque no se cuida.

HANNAH: Y habla con usted acerca de su virus o es una cosa privada que ella no le gusta hablar?

MUJER: Uhm, pues no, no le gusta hablar pero parece que ella se lo comento a otra tía mía y ella nos dijo a nosotros.

Title: Mantica-4.docx

Descriptor Info: Age: Interview Location: Residence: Interviewer: Date of Interview:

Codes Applied: Support Private/taboo

Excerpt Package: 2060

Excerpt End: 3085

HANNAH: Y durante este proceso se sentía cómoda hablando de su enfermedad con su familia, amigos o es algo más privado para usted?

MUJER: Si es algo más privado

HANNAH: Con quien se sentiría más cómoda hablando sobre el cáncer?

MUJER: Disculpe no le entendí bien

HANNAH: Bueno, me refiero que con quien puede hablar sobre su enfermedad?

MUJER: Bueno, solamente con mi medico

HANNAH: Tiene una pareja?

MUJER: Bueno, si, a él también tengo que decirle porque no puedo ocultarle anda de esto

HANNAH: Pero su pareja sabe de su enfermedad que ha tenido cáncer y su tratamiento?

MUJER: Si, si lo sabe

HANNAH: Pero prefiere hablar con un médico o una enfermera sobre su enfermedad más que con otros?

MUJER: Si

HANNAH: Y sus padres saben también?

MUJER: Bueno mi papa que es el único que está vivo, si está al tanto de mis problemas

HANNAH: Y puede hablar de eso con sus amigos o ellos no saben?

MUJER: No, solamente primero Dios que es el único que nos protege, mi médico que lo descubrió, mi pareja y mi papa saben de mis problemas

Title: Mantica-4.docx

Descriptor Info: Age: Interview Location: Residence: Interviewer: Date of Interview:

Codes Applied: Support Private/taboo

Excerpt Package: 3204

Excerpt End: 3920

HANNAH: Y sabe usted de otras enfermedades que han sufrido del cáncer de cuello uterino?

MUJER: Pues en el hospital me he encontrado con personas que también están sufriendo mi enfermedad y así como ellas lo han superado espero yo también porque mi Dios me esta ayudando muchísimo

HANNAH: Es en forma de grupos e apoyo o es solo un grupo en el hospital de otros pacientes?

MUJER: Sí, es un grupo en el hospital de otros con quienes he hecho amistad porque están viviendo la misma situación

HANNAH: Y piensa que ellos también tienen su situación de la enfermedad como algo privado o tienen maneras diferentes de compartir su experiencia?

MUJER: Bueno cada quien tiene su forma de ser y sabe a quien contarle sus cosas

Title: Poneloya-1.docx

Descriptor Info: Age: Interview Location: Residence: Interviewer: Date of Interview:

Codes Applied: Support Promiscuity

Excerpt Package: 4390

Excerpt End: 5938

ALEXANDRA: Por ejemplo si antes de recibir una prueba de Pap usted consultaría con su pareja?

MUJER: Prueba de pap? Es esa de Papanicolaou? Consulta si me la puedo hacer? No, no lo haría porque es algo sobre mi cuerpo y tengo entendido que se debe de hacer una vez al año, y si yo me quiero a mi misma y mi cuerpo debo hacerlo porque tengo entendido que las mujeres de aquí no les gusta hacérselo, porque no tienen tiempo porque deben cuidar sus casas, y eso es algo malo porque deben hacerlo para prevenir muchas enfermedades como cáncer, entre otras muchas cosas como las enfermedades de transmisión sexual, a como yo pienso la mayor parte de las mujeres con enfermedades sexuales son las del hogar porque sus esposos andan con muchas mujeres y las del hogar no se cuidan porque ellas solo están con sus maridos.

ALEXANDRA: Y la religión influye en sus decisiones acerca de su salud?

MUJER: Bueno, yo no sé, pero creo que no porque si a una cristiana le gusta alguien ella lo hace y ya, pero no sé, yo creo que lo que más influye es la crianza de los padres, porque en mi caso yo tengo seis hermanas mujeres y dos varones y nuestra mama siempre nos aconseja, ella trabaja mucho y casi no se mantiene en casa pero cuando está con nosotros nos aconseja y nos dice que debemos cuidarnos mucho de eso, porque los muchachos de hoy en día tienen sexo con muchas y no se cuidan y las que sufren las consecuencias en muchas ocasiones son las mujeres pero también hay mujeres que les gusta andar así con varios hombres y viceversa contagian a los varones.

**Time**

Title: Mantica-6.docx

Descriptor Info: Age: Interview Location: Residence: Interviewer: Date of Interview:

Codes Applied: Time

Excerpt Package: 2198

Excerpt End: 2491

ALEXANDRA: Se tardan muchos días en darte los resultados?

MUJER: No, dependiendo, siempre me han dicho aquí que eso lo mandan al HEODRA y siempre dilata una semana

ALEXANDRA: Es más rápido si vas al hospital o es lo mismo?

MUJER: Es lo mismo al final porque siempre hay acumulación de personas

Title: Los Leches-1 (1).docx

Descriptor Info: Age: Interview Location: Residence: Interviewer: Date of Interview:

Codes Applied: Time

Excerpt Package: 9976

Excerpt End: 10062

Sara: And do you live far from here?

Woman: I live about five hundred yards from here.

Title: Los Leches-1 (1).docx

Descriptor Info: Age: Interview Location: Residence: Interviewer: Date of Interview:

Codes Applied: Time

Excerpt Package: 1050

Excerpt End: 1188

Sara: And do you live in a rural or urban area?

Woman: I live here, in Los Leches very close to the Health Post, in the Sector Hermitage.

Title: Perla-3.docx

Descriptor Info: Age: Interview Location: Residence: Interviewer: Date of Interview:

Codes Applied: Time

Excerpt Package: 83

Excerpt End: 372

HANNAH: Para empezar puede decirme su experiencia con el cuidado de salud?

MUJER: Pues la experiencia no ha sido tan mal pero lo que hay que ver es que cuando estamos esperando aquí en el centro ya que somos bastante gente hay pocos médicos eso es lo que pasa y aquí la gente se desespera

Title: Los Leches-1 (1).docx

Descriptor Info: Age: Interview Location: Residence: Interviewer: Date of Interview:

Codes Applied: Time

Excerpt Package: 10150

Excerpt End: 10381

Sara: There are some accessibility problems to the health post?

Woman: Sorry I do not understand

Sara: Maybe like if there’s people who say that they can not go to the center or if is a problem for the community?

Woman: Not at all.

Title: Subtiava-2.docx

Descriptor Info: Age: Interview Location: Residence: Interviewer: Date of Interview:

Codes Applied: Time

Excerpt Package: 2158

Excerpt End: 2787

HANNAH: Y ha tenido alguna mala experiencia con el centro de salud?

MUJER: Pues a veces lo único es que te llaman muy tardadamente y lo único es que uno debe de tener paciencia porque a veces nos queremos ir rápido y no entendemos que deben atender demasiados pacientes y hay que tener paciencia para que te llamen, pero en general la atención es muy buena, yo solo este centro he visitado y está bien

HANNAH: Por cuantas horas debe de esperar para hacerse una prueba de Pap?

MUJER: A uno lo atienden rápido, a veces de cuantas personas hay esperando pero puede ser de veinte a treinta minutos pero es rápido cuando yo he venido

Title: Poneloya-2.docx

Descriptor Info: Age: Interview Location: Residence: Interviewer: Date of Interview:

Codes Applied: Time

Excerpt Package: 5795

Excerpt End: 6366

ALEXA: Y cuanta confianza tiene de que las pruebas dan información precisa acerca de su salud?

MUJER: Por lo menos a veces se tarda un mes esperando los resultados, dos meses entonces ya viene me lo dan el sobre y yo voy donde el médico y entonces me dice lo que tengo y reacciono al respecto y me dan mi tratamiento y yo lo inicio porque no hay nada más que se pueda hacer, porque a veces la pienso de que quien sabe que me va a salir porque a veces yo digo que estoy sana pero puede que no sea así porque uno nunca sabe lo que saldrá en los exámenes que le harán a uno.

Title: Subtiava-2.docx

Descriptor Info: Age: Interview Location: Residence: Interviewer: Date of Interview:

Codes Applied: Time

Excerpt Package: 67

Excerpt End: 657

HANNAH: Y para empezar cuénteme su experiencia con el cuidado de salud?

MUJER: Bueno, la atención es muy buena y de calidad porque cuando alguien está enfermo y viene lo atienden y si alguien está aún más enfermo las emergencias son atendidas más rápido y siempre que yo he venido me han atendido con calidad y con amabilidad

HANNAH: Bueno, y usualmente viene a este centro de salud?

MUJER: Si, siempre que me toca venir a mi o mi bebe aquí venimos al centro de salud de sutiava.

HANNAH: Usted vive cerca de aquí en una región urbana?

MUJER: Si, es urbano, del Calasanz dos y media al norte

Title: Santa Ana-2.docx

Descriptor Info: Age: Interview Location: Residence: Interviewer: Date of Interview:

Codes Applied: Time

Excerpt Package: 5130

Excerpt End: 5267

ALEXANDRA: Tarda mucho para recibir los resultados?

MUJER: Si dilata porque por lo menos yo me hice el mío en Junio y salió hasta ahorita

Title: Mantica-2.docx

Descriptor Info: Age: Interview Location: Residence: Interviewer: Date of Interview:

Codes Applied: Time

Excerpt Package: 2745

Excerpt End: 3152

ALEXANDRA: Cuando se ha hecho una prueba de pap tiene que esperar mucho en los centros de salud

MUJER: Bueno en algunos casos si, se debe esperar hasta un mes por los resultados pero a mí me han hecho excepciones y me los dan en ochos días

ALEXANDRA: Y por ejemplo si vas al hospital puedes recibir los resultados más rápidos

MUJER: Sí, es más rápido porque igual a los ochos días te mandan los resultados

Title: Perla-4.docx

Descriptor Info: Age: Interview Location: Residence: Interviewer: Date of Interview:

Codes Applied: Time

Excerpt Package: 9956

Excerpt End: 10673

HANNAH: Y piensa usted que cada médico pasa suficiente tiempo con el paciente para explicar cosas acerca de su salud?

MUJER: Pues no muchos, son raros y ocasionales, por ejemplo hoy he venido dos veces la primera vez dilate dos horas nunca pude pasar porque se da el caso de que el medico atiende a las embarazadas y están dilatan más que la atiendan que la mide, la pesan y eso hace que el médico en ocasiones atienda apresuradamente pero por decir ahorita solo hay una ósea faltaron dos no sé si están de vacaciones o que y eso en parte afecta en manera general al paciente porque uno se desespera y la gente enojada si uno quiere entrar a preguntar algo y no dejan porque se preocupan ya que todos nos queremos ir.

Title: Mantica-5.docx

Descriptor Info: Age: Interview Location: Residence: Interviewer: Date of Interview:

Codes Applied: Time

Excerpt Package: 1443

Excerpt End: 1616

HANNAH: Y cuantas semanas pasan usualmente para recibir los resultados?

MUJER: Bueno si ellas lo van a dejar al laboratorio, es un mes, pero si lo llevo yo, son quince días

Title: Perla-2.docx

Descriptor Info: Age: Interview Location: Residence: Interviewer: Date of Interview:

Codes Applied: Time

Excerpt Package: 6998

Excerpt End: 7288

ALEXANDRA: Y tarda mucho tiempo para esperar los resultados en los centros de salud?

MUJER: Si porque mire como discutí con la muchacha no ha querido llevar los papeles, ahí los tiene

ALEXANDRA: Y para recibir los resultados también toma mucho tiempo?

MUJER: No solo viene a recibirlos y ya

Title: Subtiava-1.docx

Descriptor Info: Age: Interview Location: Residence: Interviewer: Date of Interview:

Codes Applied: Time

Excerpt Package: 5085

Excerpt End: 5468

HANNAH: Oh usted, solo dos veces en su vida ha recibido una prueba de pap?

MUJER: Si

HANNAH: Y piensa que no necesita más porque?

MUJER: No, si es necesario pero no he tenido tiempo por el trabajo pero si voy a dedicar un dia para ir donde la doctora ver los resultados pero yo pienso y siento que estoy bien.

HANNAH: Pero si se sintiera mal?

MUJER: Yo fuera rápidamente de una vez.

Title: Perla-5.docx

Descriptor Info: Age: Interview Location: Residence: Interviewer: Date of Interview:

Codes Applied: Time

Excerpt Package: 350

Excerpt End: 807

ALEXA: Aquí como es su experiencia en los centros de salud?

MUJER: Bueno las pocas veces que he venido la atención ha sido un poco lenta porque a veces los doctores tienen que hacer otras cosas antes de atender a los pacientes pero si me he sentido atendida con ellos solo que a veces necesitamos ampliar mas las salas porque en el momento en que los pacientes están con los médicos generales tal vez ellos necesitan su sala de ginecología algo más cerrado.

Title: Perla-3.docx

Descriptor Info: Age: Interview Location: Residence: Interviewer: Date of Interview:

Codes Applied: Time

Excerpt Package: 83

Excerpt End: 372

HANNAH: Para empezar puede decirme su experiencia con el cuidado de salud?

MUJER: Pues la experiencia no ha sido tan mal pero lo que hay que ver es que cuando estamos esperando aquí en el centro ya que somos bastante gente hay pocos médicos eso es lo que pasa y aquí la gente se desespera

Title: Mantica-1.docx

Descriptor Info: Age: Interview Location: Residence: Interviewer: Date of Interview:

Codes Applied: Time Attendance

Excerpt Package: 365

Excerpt End: 833

Sara: Pero usted no va a los centros?

Mujer: No casi no, no me he enfermado solo él es el que se enferma, yo no, casi no lo visito pero antes es que venía aquí, venia y me atendían muy bien, cuando estaba pequeña venia, era muy bueno el atendimiento, te daban el medicamento porque a veces hay personas que no tienen para comprar el medicamento, aquí en la farmacia te lo dan

Sara: Cuanto tiempo le toma venir a este centro desde su casa?

Mujer: Como unos diez minutos

**Vaccine**

Title: Mantica-4.docx

Descriptor Info: Age: Interview Location: Residence: Interviewer: Date of Interview:

Codes Applied: Vaccine

Excerpt Package: 5700

Excerpt End: 6316

HANNAH: Sí, es verdad eso, sabe que existe una vacuna contra el vph?

MUJER: Si

HANNAH: Sabe si es disponible o gratis en Nicaragua?

MUJER: Pues la información que yo recibí es que aún no está en Nicaragua y que tiene un costo muy alto

HANNAH: Si fuera disponible y gratis es algo que quisiera para sus hijas?

MUJER: Si, lo haría

HANNAH: Sin duda alguna o es algo que tendría que pensar más?

MUJER: Pues la verdad en estos momentos a como me siento tan agobiada hay momentos que me siento sin palabras por el mismo nervios por ejemplo ahorita y después al esperar el resultado será peor ya que no sé cómo saldrá todo

Title: Perla-2.docx

Descriptor Info: Age: Interview Location: Residence: Interviewer: Date of Interview:

Codes Applied: Vaccine

Excerpt Package: 6398

Excerpt End: 6478

ALEXANDRA: Y ha escuchado de la acuna del virus y el cáncer cervical?

MUJER: No.

Title: Mantica-2.docx

Descriptor Info: Age: Interview Location: Residence: Interviewer: Date of Interview:

Codes Applied: Vaccine

Excerpt Package: 8018

Excerpt End: 8282

ALEXANDRA: Ha oído alguna vez de la vacuna del vph y del cáncer cervical

MUJER: No. De eso no he escuchado

ALEXANDRA: Y si habría una vacuna se la pondría a su hija

MUJER: Si hubiera una vacuna contra eso, si, o al menos para prevenir esa enfermedad yo digo que si

Title: Subtiava-2.docx

Descriptor Info: Age: Interview Location: Residence: Interviewer: Date of Interview:

Codes Applied: Vaccine

Excerpt Package: 8440

Excerpt End: 9301

HANNAH: Y mi pregunta final es si ha oído hablar sobre la vacuna del virus de papiloma humano? Hay una vacuna?

MUJER: Vacuna? No lo sé, no tengo conocimiento sobre eso.

HANNAH: Bueno, hay una vacuna para el virus y el cáncer cervical y es recomendada para las niñas de once y doce años de edad y es un poco complicado porque requiere tres inyecciones en un periodo de seis meses por eso también es muy costosa y no es disponible en Nicaragua por ahora pero ojala que si en el futuro, en una situación hipotética si en un futuro fuera gratis y disponible en el país aceptaría ponérsela a su hija?

MUJER: Si claro que si, en un futuro si porque sería bueno para su salud y casualmente tengo una niña de diez años va a cumplir once ojala que cuando la aprueben ella aun participar de la vacunación.

HANNAH: Entonces está interesada en la vacuna?

MUJER: Si, así es

Title: Perla-5.docx

Descriptor Info: Age: Interview Location: Residence: Interviewer: Date of Interview:

Codes Applied: Vaccine

Excerpt Package: 14884

Excerpt End: 14980

ALEXA: Alguna vez ha escuchado acerca de la vacuna del papiloma y el cáncer cervical?

MUJER: No.

Title: Mantica-5.docx

Descriptor Info: Age: Interview Location: Residence: Interviewer: Date of Interview:

Codes Applied: Vaccine

Excerpt Package: 6146

Excerpt End: 6855

HANNAH: Ha oído hablar de la vacuna contra el VPH?

MUJER: Si pero a mí no me la han puesto.

HANNAH: Oh pero sabe si esta vacuna es disponible o gratis aquí en Nicaragua? Sabe eso?

MUJER: No, no lo sé.

HANNAH: La vacuna es muy eficaz y recomendada para prevenir el VPH y es para las niñas de once y doce años de edad, pero por ahora no es disponible ni gratis en Nicaragua porque es muy costosa y son tres inyecciones en seis meses, pero si la inyección fuera disponible y gratuita pensaría usted en ponérsela a sus hijas?

MUJER: Si hubiera disponible aquí, aunque hubiera que comprarla y sean caras yo se las pondría a mi hija

HANNAH: Si no fuera cara?

MUJER: No aunque fuera cara, porque primero es la salud.

Title: Los Leches-2.docx

Descriptor Info: Age: Interview Location: Residence: Interviewer: Date of Interview:

Codes Applied: Vaccine

Excerpt Package: 4912

Excerpt End: 5411

HANNAH: Ha oído de la vacuna contra el vph y el cáncer cervical alguna vez?

MUJER: No

HANNAH: Bueno esta vacuna no está disponible aun en Nicaragua pero es recomendada para las niñas de once y doce años de edad para proteger contra el vph y cáncer cervical, si la vacuna fuera disponible y gratis aquí se la pondría a sus hijas?

MUJER: Sí, claro que sí, porque si hubiera les diría que se la pongan

HANNAH: Y tiene algunas preocupaciones acerca de las vacunas?

MUJER: No, todo bien todas las vacunas

Title: Mantica-4.docx

Descriptor Info: Age: Interview Location: Residence: Interviewer: Date of Interview:

Codes Applied: Vaccine

Excerpt Package: 5700

Excerpt End: 6316

HANNAH: Sí, es verdad eso, sabe que existe una vacuna contra el vph?

MUJER: Si

HANNAH: Sabe si es disponible o gratis en Nicaragua?

MUJER: Pues la información que yo recibí es que aún no está en Nicaragua y que tiene un costo muy alto

HANNAH: Si fuera disponible y gratis es algo que quisiera para sus hijas?

MUJER: Si, lo haría

HANNAH: Sin duda alguna o es algo que tendría que pensar más?

MUJER: Pues la verdad en estos momentos a como me siento tan agobiada hay momentos que me siento sin palabras por el mismo nervios por ejemplo ahorita y después al esperar el resultado será peor ya que no sé cómo saldrá todo

Title: Mantica-1.docx

Descriptor Info: Age: Interview Location: Residence: Interviewer: Date of Interview:

Codes Applied: Vaccine

Excerpt Package: 8685

Excerpt End: 9178

Sara: Ha oído hablar de la vacuna contra el virus del papiloma humano?

Mujer: Sí, eso le iba a decir, dicen que se la debe poner una muchacha virgen

Sara: Si, la vacuna es para las chicas adolescentes entre once y doce años y protege contra el vph y otras enfermedades, pero si fuera disponible y gratis que probabilidades hay de que se la ponga a su hija si tiene una?

Mujer: Yo le diría que se la ponga para evitar esas enfermedades

Sara: Usted confía en las vacunas?

Mujer: Si, si la tengo.

Title: Santa Ana-3.docx

Descriptor Info: Age: Interview Location: Residence: Interviewer: Date of Interview:

Codes Applied: Vaccine

Excerpt Package: 8413

Excerpt End: 9173

HANNAH: Ha oído de la vacuna contra del vph y el cáncer cervical alguna vez?

MUJER: Si he escuchado pero casi no tengo la información completa, y no sé si será cierto o no es cierto

HANNAH: Hay una vacuna contra el vph y el cáncer cervical, pero requiere tres inyecciones en un periodo de seis meses y es recomendado para las niñas de once y doce años de edad pero es algo muy complicado porque requiere tres inyecciones y por eso también es muy costosa y aún no está siendo usada en Nicaragua pero hay pilotajes en Perú pero espero venga muy pronto, pero si fuera disponible y gratis piensa que habrían personas que estarían interesada en esta vacuna?

MUJER: Si, claro que sí y sería muy bueno porque estamos combatiendo y así estaríamos vacunadas contra eso.

Title: Perla-3.docx

Descriptor Info: Age: Interview Location: Residence: Interviewer: Date of Interview:

Codes Applied: Vaccine

Excerpt Package: 14731

Excerpt End: 15691

HANNAH: Ha oído de la vacuna en contra del VPH y el cáncer de cuello uterino alguna vez?

MUJER: No, hasta ahora que usted me está diciendo.

HANNAH: La vacuna es recomendada para chicas adolescentes de once y doce años de edad, pero no es ofrecido en Nicaragua ahora, pero en una situación hipotética en un futuro si la vacuna fuera completamente gratuita y disponible.

MUJER: Ah es que es cara?

HANNAH: Si.

MUJER: Me imagino porque como es un medicamento que no lo requiere toda la población es lógico que debe de tener un precio.

HANNAH: Si es una vacuna que requiere tres inyecciones en un periodo de seis meses y por eso es un poco complicado para administrar y también es un poco caro pero si fuera gratis y disponible piensa que usted se la pondría a su hija?

MUJER: Claro, a mi hija si de aquí a que tenga once años tal vez ya este ese medicamento para prevenir el cáncer y se lo compro sino pues ni modo a orientarla mentalmente acerca de la sexualidad.

Title: Los Leches-3.docx

Descriptor Info: Age: Interview Location: Residence: Interviewer: Date of Interview:

Codes Applied: Vaccine

Excerpt Package: 6087

Excerpt End: 6857

SARA: Ha oído hablar de la vacuna contra el virus del papiloma humano?

MUJER: He oído poco pero dicen que es algo que no la quita sino que solo protege

SARA: Si, la vacuna es para las niñas de once y doce años de edad y es para prevenir el virus papiloma, si fuera disponible y gratis que probabilidades hay de que cuando tenga esta de edad se la ponga?

MUJER: Sí, yo diría que si

SARA: Y por qué?

MUJER: Para que en un futuro no tenga eso pero una pregunta esa vacuna hasta que edad protege

SARA: Protege del cáncer de cuello uterino, el virus de papiloma humano y verrugas genitales y otros.

MUJER: Pero solo a las niñas de esa edad?

SARA: Porque es para antes de que inician las relaciones sexuales

MUJER: Y si uno ya las ha iniciado?

SARA: Entonces no es efectiva.

Title: Santa Ana-1.docx

Descriptor Info: Age: Interview Location: Residence: Interviewer: Date of Interview:

Codes Applied: Vaccine

Excerpt Package: 6022

Excerpt End: 6521

SARA: Ha oído hablar de la vacuna del virus de papiloma humano

MUJER: Si, la he escuchado pero no he obtenido información acerca de ella

SARA: Donde la ha escuchado

MUJER: La escuche en mi familia, pero específicamente no detallaron de que se trata

SARA: Bueno la vacuna es para chicas adolescentes de once y doce años de edad para prevenir el virus papiloma si fuera disponible y gratis que probabilidad existe de ponérsela a su hija cuando tenga esta edad?

MUJER: Pues mucha porque así la salvaría

Title: Perla-4.docx

Descriptor Info: Age: Interview Location: Residence: Interviewer: Date of Interview:

Codes Applied: Vaccine

Excerpt Package: 7815

Excerpt End: 8580

HANNAH: Ha oído de la vacuna del vph y el cáncer de cuello uterino?

MUJER: Si pero pienso que a estas alturas yo no me puedo poner esa vacuna, por mi edad

HANNAH: Si, así es, ahora la vacuna no es ofrecida en Nicaragua, pero en un futuro espero de que sí, porque es muy eficaz previniendo el cáncer de cuello uterino y es recomendada para niñas de once y doce años de edad porque es para las personas que aún no han iniciado las relaciones sexuales

MUJER: Desde los quince años es?

HANNAH: No, desde los diez a doce, porque es necesario antes de empezar las relaciones sexuales pero si la vacuna fuera gratis y disponible aquí en Nicaragua piensa que usted le pondría la vacuna a sus hijas o amigas menores?

MUJER: Si, así es, con la esperanza de que no nos afecte

Title: Perla-3.docx

Descriptor Info: Age: Interview Location: Residence: Interviewer: Date of Interview:

Codes Applied: Vaccine

Excerpt Package: 14731

Excerpt End: 15691

HANNAH: Ha oído de la vacuna en contra del VPH y el cáncer de cuello uterino alguna vez?

MUJER: No, hasta ahora que usted me está diciendo.

HANNAH: La vacuna es recomendada para chicas adolescentes de once y doce años de edad, pero no es ofrecido en Nicaragua ahora, pero en una situación hipotética en un futuro si la vacuna fuera completamente gratuita y disponible.

MUJER: Ah es que es cara?

HANNAH: Si.

MUJER: Me imagino porque como es un medicamento que no lo requiere toda la población es lógico que debe de tener un precio.

HANNAH: Si es una vacuna que requiere tres inyecciones en un periodo de seis meses y por eso es un poco complicado para administrar y también es un poco caro pero si fuera gratis y disponible piensa que usted se la pondría a su hija?

MUJER: Claro, a mi hija si de aquí a que tenga once años tal vez ya este ese medicamento para prevenir el cáncer y se lo compro sino pues ni modo a orientarla mentalmente acerca de la sexualidad.

Title: Los Leches-1 (1).docx

Descriptor Info: Age: Interview Location: Residence: Interviewer: Date of Interview:

Codes Applied: Vaccine

Excerpt Package: 8697

Excerpt End: 9975

Sara: My final question is have heard of the vaccine human papillomavirus?

Woman: No, I haven’t, only of others vaccines such as polio, tetanus and pneumococcus.

Sara: But not for the sexual diseases?

Woman: No, already it exists?

Sara: Exist but not in Nicaragua.

Woman: Just the Lord in heaven have it.

Sara: Do you believe in the vaccines?

Woman: Yes, I believe it, because before there was a lot of problems of polio and the vaccines works.

Sara: This vaccine is for girls of eleven and twelve years old and protects versus this human papillomavirus and also genital warts, if is available and free there is a possibility to put the vaccine to your daughter if hypothetically has this age?

Woman: Sorry, I did not heard you.

Sara: Again, if this vaccine was available and free what chance exists to put this vaccine to your daughter?

Woman: What chance exist? Like what? That one come here?

Sara: Yes, came here and the vaccine is available for the girls

Woman: Just bring them here and that way they put them, because if I don’t bring her, how they are going to put her, because everyone must do their part, bringing them to have it applied, because at the house there’s nothing we can do, but here they go out every Wednesday, go to homes, to walk there, burning the poor.

Title: Subtiava-1.docx

Descriptor Info: Age: Interview Location: Residence: Interviewer: Date of Interview:

Codes Applied: Vaccine

Excerpt Package: 17989

Excerpt End: 18499

HANNAH: Y ha oído de la vacuna del VPH y el cáncer cervical alguna vez?

MUJER: No.

HANNAH: Hay una vacuna y es recomendada para las niñas de once y doce años de edad pero requiere tres inyecciones en un periodo de seis meses y por eso es muy costosa y no es disponible en Nicaragua aun pero en el futuro ojala que sí y si fuera disponible y gratis en una situación hipotética le pondría esta vacuna a su hija?

MUJER: Si para que este protegida

HANNAH: Del VPH y del cáncer de cuello uterino también

MUJER: Si.
